# Mining Gene Expression Signature for the Detection of Pre-Malignant Melanocytes and Early Melanomas with Risk for Metastasis

**DOI:** 10.1371/journal.pone.0044800

**Published:** 2012-09-11

**Authors:** Camila Ferreira de Souza, Patrícia Xander, Ana Carolina Monteiro, Amanda Gonçalves dos Santos Silva, Débora Castanheira Pereira da Silva, Sabine Mai, Viviane Bernardo, José Daniel Lopes, Miriam Galvonas Jasiulionis

**Affiliations:** 1 Departamento de Farmacologia, Universidade Federal de São Paulo, São Paulo, São Paulo, Brazil; 2 Departamento de Ciências Biológicas, Universidade Federal de São Paulo, Diadema, São Paulo, Brazil; 3 CIPE, Hospital A.C. Camargo, São Paulo, SP, Brazil; 4 Departamento de Oncologia Cutânea, Hospital A.C. Camargo, São Paulo, São Paulo, Brazil; 5 Manitoba Institute of Cell Biology, The University of Manitoba, Cancer Care Manitoba, Winnipeg, Manitoba, Canada; 6 Departamento de Informática em Saúde, Universidade Federal de São Paulo, São Paulo, São Paulo, Brazil; 7 Departamento de Microbiologia, Imunologia e Parasitologia, Universidade Federal de São Paulo, São Paulo, São Paulo, Brazil; University of Tennessee, United States of America

## Abstract

**Background:**

Metastatic melanoma is a highly aggressive skin cancer and currently resistant to systemic therapy. Melanomas may involve genetic, epigenetic and metabolic abnormalities. Evidence is emerging that epigenetic changes might play a significant role in tumor cell plasticity and metastatic phenotype of melanoma cells.

**Principal findings:**

In this study, we developed a systematic approach to identify genes implicated in melanoma progression. To do this, we used the Affymetrix GeneChip Arrays to screen 34,000 mouse transcripts in melan-a melanocytes, 4C pre-malignant melanocytes, 4C11− non-metastatic and 4C11+ metastatic melanoma cell lines. The genome-wide association studies revealed pathways commonly over-represented in the transition from immortalized to pre-malignant stage, and under-represented in the transition from non-metastatic to metastatic stage. Additionally, the treatment of cells with 10 µM 5-aza-2′-deoxycytidine (5AzaCdR) for 48 hours allowed us to identify genes differentially re-expressed at specific stages of melan-a malignant transformation. Treatment of human primary melanocytes with the demethylating agent 5AzaCdR in combination to the histone deacetylase inhibitor Trichostatin A (TSA) revealed changes on melanocyte morphology and gene expression which could be an indicator of epigenetic flexibility in normal melanocytes. Moreover, changes on gene expression recognized by affecting the melanocyte biology (*NDRG2* and *VDR*), phenotype of metastatic melanoma cells (*HSPB1* and *SERPINE1*) and response to cancer therapy (*CTCF*, *NSD1* and *SRC*) were found when Mel-2 and/or Mel-3-derived patient metastases were exposed to 5AzaCdR plus TSA treatment. Hierarchical clustering and network analyses in a panel of five patient-derived metastatic melanoma cells showed gene interactions that have never been described in melanomas.

**Significance:**

Despite the heterogeneity observed in melanomas, this study demonstrates the utility of our murine melanoma progression model to identify molecular markers commonly perturbed in metastasis. Additionally, the novel gene expression signature identified here may be useful in the future into a model more closely related to translational research.

## Introduction

Metastatic melanoma is a highly aggressive and drug-resistant skin cancer and has a poor prognosis, with a 1-year survival rate ranging from 33% to 62% [1], [2]. The current methodology to diagnose primary melanomas is based on histomorphologic features, however such classification system has remained of limited clinical relevance once metastases have developed. The current prevailing opinion is that melanomas are more variable at the molecular level than can be observed macro/microscopically [3], [4]. For anti-melanoma strategies to be successful, it is important the better understanding about the molecular mechanisms that mediate phenotype-switching in melanomas, since these biomarkers could drive metastatic disease. According to the genetic model to explain the pathways towards metastasis, the acquisition of mutations that promote invasiveness leads to cells migrating away from the primary tumor before arriving in a new site where they divide to form a new metastasis. As the genetic lesions are irreversible, metastases will be genetically distinct from the primary tumor. In contrast of this, there is a hypothesis which suggests that metastases arise because of microenvironment-driven changes to a cell's phenotype, leading to some cells acquiring a slow proliferating, stem cell-like phenotype with invasive potential. Given the appropriate signals, these cells will migrate to find a new niche where a different microenvironment will lead to either a resumption of proliferation and formation of a new metastasis or dormancy [5]. As the epigenetic modifications can be modulated by changes in the environment, leading to a reprogramming of gene expression patterns in mammals [6], [7], it is plausible to hypothesize that there is a biological relationship between epigenetics and microenvironment-driven changes in gene expression, which would lead to a phenotype-switching in melanomas. In fact, it has become clear that a dynamic reprogramming in epigenetic marks has been positively correlated with aberrant gene expression during melanocytic neoplasia, including our model of study [8]–[11]. Epigenetic information is propagated during somatic cell divisions, and is critical for preserving gene expression patterns and cellular identity. Epigenetic regulation of gene expression involves the interplay of DNA methylation, histone modifications, nucleosome occupancy, and microRNAs. DNA methylation patterns are tightly correlated with chromatin architecture. Active regions of the chromatin, which enable gene expression, are associated with hypomethylated DNA, whereas hypermethylated DNA is packaged in inactive chromatin. Histone deacetylation is a global mark of gene silencing. The interrelation between DNA methyltransferases (DNMTs) and histone deacetylases (HDACs) associated with the dynamic reversibility of epigenetic signatures in somatic cells, has been offered opportunities for testing drugs that can re-express critical silenced-genes in cancer [12]–[13]. The main purpose of our research was to identify novel markers in early and later stages of melan-a malignant transformation. The molecular markers deregulated earlier in melan-a malignant transformation could provide insights into the aetiology and pathogenesis of melanomas. In addition, they would be useful to predict a functional phenotype associated with metastasis. Apart from this, the molecular markers specifically deregulated at metastatic stage could confer selective clonal growth advantage. Here, we used the high-throughput Affymetrix microarray approach to conduct class-comparison experiments to identify significantly up- and down-regulated genes in each transition of malignant transformation of melan-a mouse melanocyte lineage: from melan-a to 4C, 4C to 4C11−, and 4C11− to 4C11+ cells. The 4C (pre-malignant melanocytes), 4C11− (non-metastatic melanoma) and 4C11+ (metastatic melanoma) cell lines were established after sequential detachment/re-adhesion cycles of melan-a melanocytes. Next, the class-comparison experiments were done in melan-a, 4C, 4C11− and 4C11+ cells previously exposed to the demethylating agent 5-aza-2′-deoxycytidine (5AzaCdR). Genome-wide association studies allowed us to identify candidate down-regulated genes whose expression could be modulated, directly or indirectly, by 5AzaCdR treatment at several stages of melanoma progression. A mining gene expression signature was found and validated in human specimens untreated and previously treated with 5AzaCdR in combination to the HDAC inhibitor Trichostatin A (TSA). Our data open new avenues for the detection of early melanocytic lesions with high risk for metastasis, meanwhile provide data which might be used in the future for the incorporation of new metastasis-related biomarkers into a model more closely related to translational research.

## Results

### Tumorigenicity and Metastatic Capabilities Were Acquired by melan-a Melanocytes after Detachment/Re-adhesion Cycles

**Figure 1:**
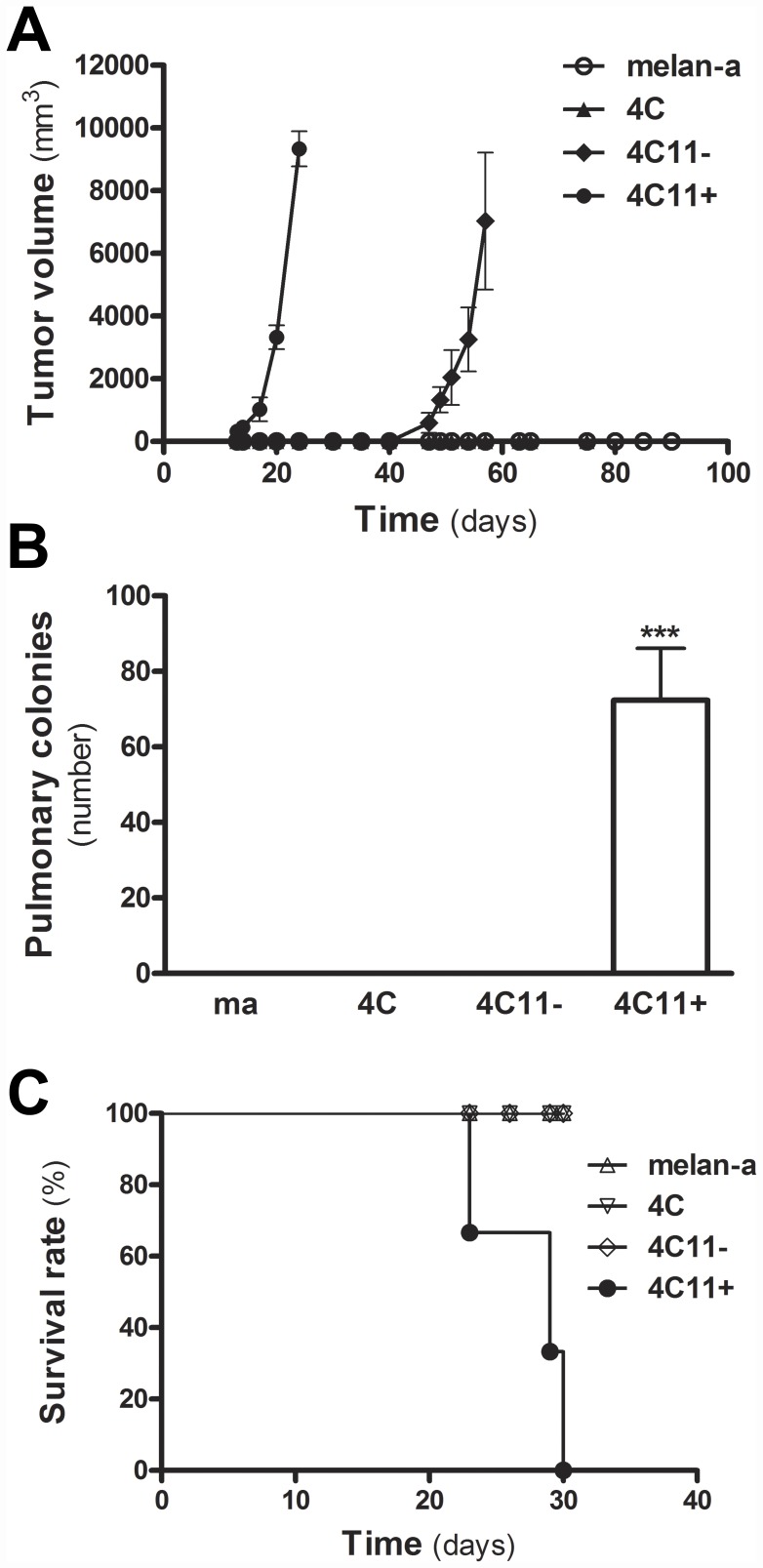
Tumorigenicity and Metastatic Capabilities Were Acquired by melan-a Melanocytes after Detachment/Re-adhesion Cycles. (**A**) Tumorigenicity assay *in vivo*. Mice were subcutaneously injected with melan-a melanocyte lineage and its derived cell lines 4C, 4C11− and 4C11+ (2.5x10^5^ cells per mouse). Tumor volume was measured during 100 days (*n* = 5 animals for each group, *P*<0.01 by One-Way ANOVA test for significance). (**B**) Metastasis capacity *in vivo*. Lungs of mice were analyzed after tail vein injection of 4C11− and 4C11+ melanoma cell lines (2.5x10^5^ cells per mouse). Melan-a and 4C non-tumorigenic melanocyte lineages were used as negative controls. Tumor multiplicity is indicated by the number of lung tumor nodules formed by 4C11+ melanoma cell line (*n* = 5 animals for each group, *P*<0.0001 by One-Way ANOVA test for significance). (**C**) Survival curves of mice injected intravenously with 4C11− and 4C11+ melanoma cell lines (2.5x10^5^ cells per mouse) (*n* = 5 animals for each group, *P*<0.0001 by Log-rank test for significance). Poor clinical outcomes were observed for all animals injected with 4C11+ metastatic melanoma cell line.

The metastatic capacity of a melanoma cell subpopulation almost always results in the successful of this neoplastic disease, as evaluated by growing mortality rates worldwide in patients with stage IV melanomas (American Joint Committee on Cancer) [2]. Based on this, we first investigated whether cell lines established from melan-a melanocytes after detachment/re-adhesion cycles showed tumorigenicity property. As previously described, melan-a and 4C melanocyte lineages were not able to grow as tumors when inoculated subcutaneously into C57Bl6 syngeneic mice, whereas 4C11− and 4C11+ cell lines showed tumorigenic potential [14], [15]. Based on the *in vivo* growth profile, we characterized the 4C11− and 4C11+ cell lines as slow- and fast-growing melanomas, respectively, as evaluated by differences in latency times for tumor appearance (*P*<0.01) (**Figure 1A**). We next evaluated whether 4C11− and 4C11+ melanoma cell lines presented metastatic capacity. Using C57Bl6 syngeneic mice, 4C11+ melanoma cell line was shown to be able to grow as macrometastatic pulmonary foci, as evaluated by tumor multiplicity (*P*<0.0001) (**Figure 1B**). Importantly, animals inoculated intravenously with 4C11+ melanoma cell line (2.5x10^5^ cells per mouse) displayed overall survival rates of 30 days (*P*<0.0001) (**Figure 1C**).

### Karyotype Evolution

The karyotypic instability can act as an indicator of cancer aggressiveness [16]. As such, we next analyzed karyotypic aberrations that accompanied the tumorigenic conversion of melan-a melanocyte lineage. It is worth mentioning that melan-a is a cell line that was first described as having a normal diploid karyotype [14]. In addition, melan-a cell line displayed no structural chromosomal abnormalities [17]. In this article, evaluation of chromosomal abnormalities of well-established melanoma cell lines was performed using spectral karyotyping (SKY). Twenty metaphases per cell line examined were analyzed. In general, the results of our chromosome analyses combined with the findings previously reported by Silva *et al* [17] revealed specific non-random structural abnormalities in chromosomes 6, 8, 11, 14, 15, and X in pre-malignant melanocytes and early melanomas (4C and 4C11− cell lines, respectively). Importantly, here we are showing that the tumorigenic phenotype was marked by the appearance of a unique translocation of T[7;X], which was seen in 6 of 20 metaphases examined. Besides that, the 4C11+ metastatic melanoma cell line displayed a complex pattern of numerical and structural aberrations that may contain potential pathological consequences. Such metastasis-related aberrations could readily be distinguished by imbalances in the number of chromosomes, Robertsonian fusions involving the chromosomes 8 and 12, and less centromere fragments than 4C11− non-metastatic melanoma cell line as well (*P*<0.0001) (**Table 1**, **Figure S1**).

**Table 1:** An overview of chromosomal aberrations during melanoma progression.

Cell Line	Chromosome Number	Centromere Fragments	Recurrent Structural Chromosomal Changes
melan-a^*^	54	21	None
4C^*^	51	31	T[8]; [14] {19}/T[14]; [8] {21}/T[15;X] {18}/F[8]; [6] {4}/Del[11] {18}
4C11−	51	31	T[8]; [14] {13}/T[14]; [8] {14}/ T[7;X] {6}/T[15;X] {11}/F[8]; [6] {19}/Del[11] {16}
4C11+	70	5	T[1]; [16] {19}/T[8]; [1] {20}/T[1]; [12] {10}/T[3]; [13] {19}/T[13]; [3] {15}/T[X;15] {15}/Rb[8.12] {14}/Rb[12.12] {30}/Del[1] {18}/Del[11] {16}

The karyotype descriptions indicated by '*'are derivatives from analyses previously performed by Silva *et al* [17].

The number of chromosomes and centromere fragments in the cell line panel are derivatives from the analyses of 20 metaphases per cell line examined.

The braces indicate how often each rearrangement was seen in 20 methaphases.

Del: deletion; F: fusion; Rb: Robertsonian fusion; T: translocation.

### Gene Expression Profiles of melan-a Melanocytes and melan-a-Derived Cell Lines Revealed Pathways Commonly Perturbed in Pre-Malignant Melanocytes and Metastatic Melanomas

**Figure 2:**
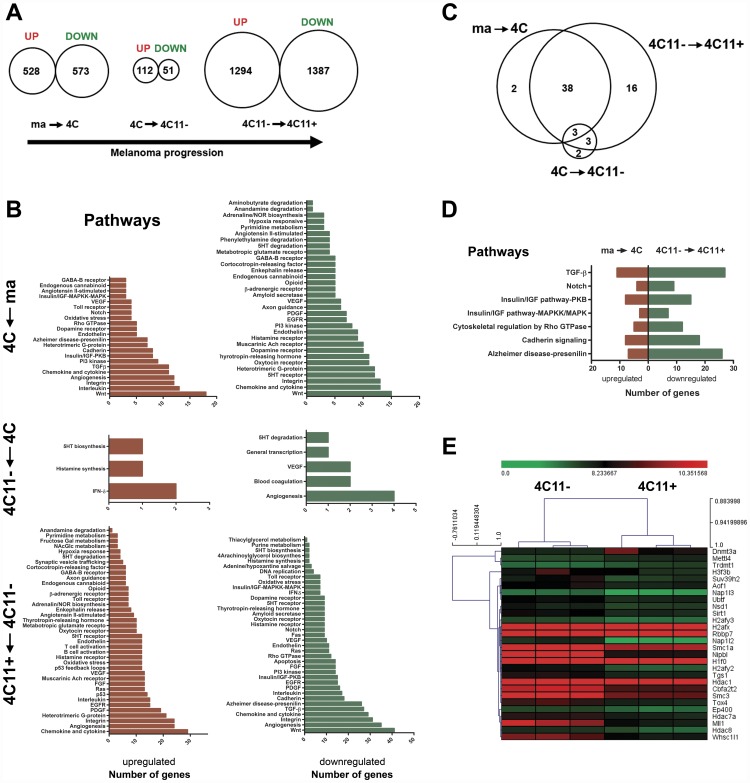
Pre-Malignant Melanocytes and Metastatic Melanomas Shared Deregulated Pathways. (**A**) Gene expression profiles of melan-a, 4C, 4C11– and 4C11+ cell lines revealed differentially up- and down-regulated genes in the transitions associated with malignant transformation of melan-a melanocytes (from melan-a to 4C, 4C to 4C11−, and 4C11− to 4C11+ cells). Transcripts were selected as statistically significant by the unpairwise two-class SAM analysis (FDR and *Q*-values<0.05). (**B**) PANTHER software was used to determine pathway profiles deregulated in the course of melanoma progression. (**C**) Venn diagram pointed out pathways deregulated at specific stages of melan-a malignant transformation. (**D**) Identification of pathways commonly over-represented in 4C pre-malignant melanocytes in relation to melan-a cells, and under-represented in 4C11+ metastatic melanoma cell line in relation to 4C11− non-metastatic melanoma cells. (**E**) Epigenetic signature as a candidate marker associated with the transition from non-metastatic to metastatic phenotype. ma: immortalized, but non-tumorigenic, melan-a melanocyte lineage; 4C: pre-malignant melanocyte lineage; 4C11−: non-metastatic melanoma cell line, and 4C11+: metastatic melanoma cell line.

As the first step in identifying up- and down-regulated genes associated with melan-a malignant transformation and melanoma progression, we identified significantly differentially expressed genes with mRNA extracted from melan-a, 4C, 4C11–– and 4C11+ cell monolayers. The high-throughput Affymetrix microarray approach was used as a platform screening (GeneChip Mouse Genome 430_2.0, format 49; Affymetrix Inc., Santa Clara, CA). In the chosen arrays, there are 45,101 probe sets designed to investigate approximately 34,000 mouse transcripts (the whole genome from *Mus musculus*). The experiments were carried out in three biological replicates. Class-comparison experiments were performed, and transcripts were selected as statistically significant by the unpairwise two-class SAM analysis (FDR and *Q*-values<0.05) by using 700 random permutations as follows: I) gene expression profile of the parental melan-a melanocyte lineage versus its 4C pre-malignant melanocyte counterpart: 528 up- and 573 down-regulated genes in 4C pre-malignant melanocytes when compared with melan-a melanocyte lineage (stage I of melan-a malignant transformation); II) gene expression profile of 4C pre-malignant melanocyte lineage versus its 4C11− non-metastatic melanoma cell counterpart: 112 up- and 51 down-regulated genes in 4C11− non-metastatic melanoma cell line when compared with 4C pre-malignant melanocyte lineage (stage II of melan-a malignant transformation); III) gene expression profile of 4C11− non-metastatic melanoma cell line versus its 4C11+ metastatic melanoma cell counterpart: 1294 up- and 1387 down-regulated genes in 4C11+ metastatic melanoma cell line when compared with 4C11− non-metastatic melanoma cell (stage III of melan-a malignant transformation) (**Figure 2A**). PANTHER software was used to determine the pathway profiles deregulated in the course of melan-a malignant transformation. Therefore, we investigated whether the proteins encoded by the genes identified as differentially expressed during the establishment of a melanoma-like phenotype were connected in known protein interaction signaling pathways. Following detailed manual annotation, the resulting pathways were ranked according to the number of genes involved in each pathway. Within the group corresponding to the transition from melan-a to 4C, representative genes from 22 and 32 pathways had undergone up- and down-regulation, respectively. Besides that, within the group corresponding to 4C to 4C11− transition, we observed that representative genes from 3 and 5 pathways had undergone up- and down-regulation, respectively. In addition, representative genes from 39 and 37 pathways had undergone up- and down-regulation, respectively, within the group corresponding to 4C11− to 4C11+ transition (**Figure 2B**). Additionally, since we have observed no intersection between up- and down-regulated genes within each group (**Figure 2A**) the presence of a switch of genes was found for pathways within the groups ma versus 4C, and 4C11− versus 4C11+ as follows: I) ma versus 4C: *Angiotensin II-stimulated*, *Inflammation mediated by chemokine and cytokine*, *Dopamine receptor*, *Endogenous cannabinoid*, *Endothelin*, *GABA-B receptor*, *Heterotrimeric G-protein*, *Integrin*, *PI3 kinase*, *VEGF*, and *Wnt*. II) 4C11− versus 4C11+: *5HT receptor*, *Angiogenesis*, *Inflammation mediated by chemokine and cytokine*, *EGFR*, *Endothelin*, *FGF*, *Histamine receptor*, *Integrin*, *Interleukin*, *Oxidative stress*, *Oxytocin receptor*, *PDGF*, *Ras*, *Thyrotropin-releasing hormone*, *Toll receptor*, and *VEGF*. (**Figure 2B**). Moreover, genes that significantly contributed to stage-specific pathways were also identified. Thus, deregulation of pathways *Aminobutyrate degradation* and *Phenylethylamine degradation* characterized the transition from melan-a melanocytes to 4C pre-malignant melanocyte lineage. Besides that, the pathways *Blood coagulation* and *General transcription by RNA polymerase I* marked the transition from 4C pre-malignant melanocytes to 4C11− non-metastatic melanoma cell line. Furthermore, sixteen signaling pathways marked the acquisition of a metastatic phenotype as follows: *2 Arachidonoylglycerol biosynthesis*, *Adenine/hypoxanthine salvage*, *Apoptosis*, *B cell activation*, *DNA replication*, *Fas*, *FGF*, *Fructose galactose metabolism*, *N-acetylglucosamine metabolism*, *P53*, *P53 feedback loops 2*, *Purine metabolism*, *Ras*, *Synaptic vesicle trafficking*, *T cell activation*, and *Triacylglycerol metabolism* (**Figure 2B-2C**). Finally, we investigated whether the genes from different class-comparison experiments shared one or more cellular pathways. As a result, we found pathways commonly perturbed between the transitions from melan-a melanocytes to 4C pre-malignant melanocytes and 4C11− non-metastatic to 4C11+ metastatic melanoma cells. Therefore, we noted that genes from the pathways *TGF-β*, *Notch*, *Insulin/IGF pathway-PKB*, *Insulin/IGF pathway MAPKK/MAPK*, *Cytoskeletal regulation by Rho GTPase*, *Cadherin signaling*, and *Alzheimer disease-presenilin signaling* were over-represented in 4C pre-malignant melanocytes in relation to melan-a cells, and under-represented in 4C11+ metastatic melanoma cell line in relation to 4C11− non-metastatic melanoma cells. Furthermore, we also observed neuroendocrine pathways commonly down-regulated in the transition from melan-a melanocytes to 4C pre-malignant melanocytes, and up-regulated in the transition from 4C11− non-metastatic to 4C11+ metastatic melanoma cell lines, as follows: *5HT degradation*, *Adrenaline/NOR biosynthesis*, *Cortocotropin-releasing factor*, *Muscarinic acetylcholine receptor*, *Opioid* and *β-adrenergic receptor* as well. Interestingly, the transition from 4C pre-malignant melanocytes to 4C11− non-metastatic melanoma cells was marked by the over-representation of genes from the *Histamine synthesis*-related pathway. Nonetheless, such pathway was over-represented in the transition from 4C11− non-metastatic melanoma to 4C11+ metastatic melanoma cells. Therefore, we provide evidence that stress-driven changes in the environment can impair the neuroendocrine activity of the melanocytes in the initial stages associated with malignant transformation of melanocytes. Together, these results provide insights into the aetiology and pathogenesis of malignant melanomas by suggesting that early melanocytic lesions would be useful to predict a functional phenotype associated with metastasis (**Figure 2B-2D**). Importantly, perturbations on the expression of genes associated with epigenetic machinery were identified as molecular markers of tumor progression in our melanoma model (**Figure 2E**) [11].

### Demethylating Agent 5AzaCdR Differentially Modulated Gene Expression at Specific Stages of Melanoma Progression

**Figure 3:**
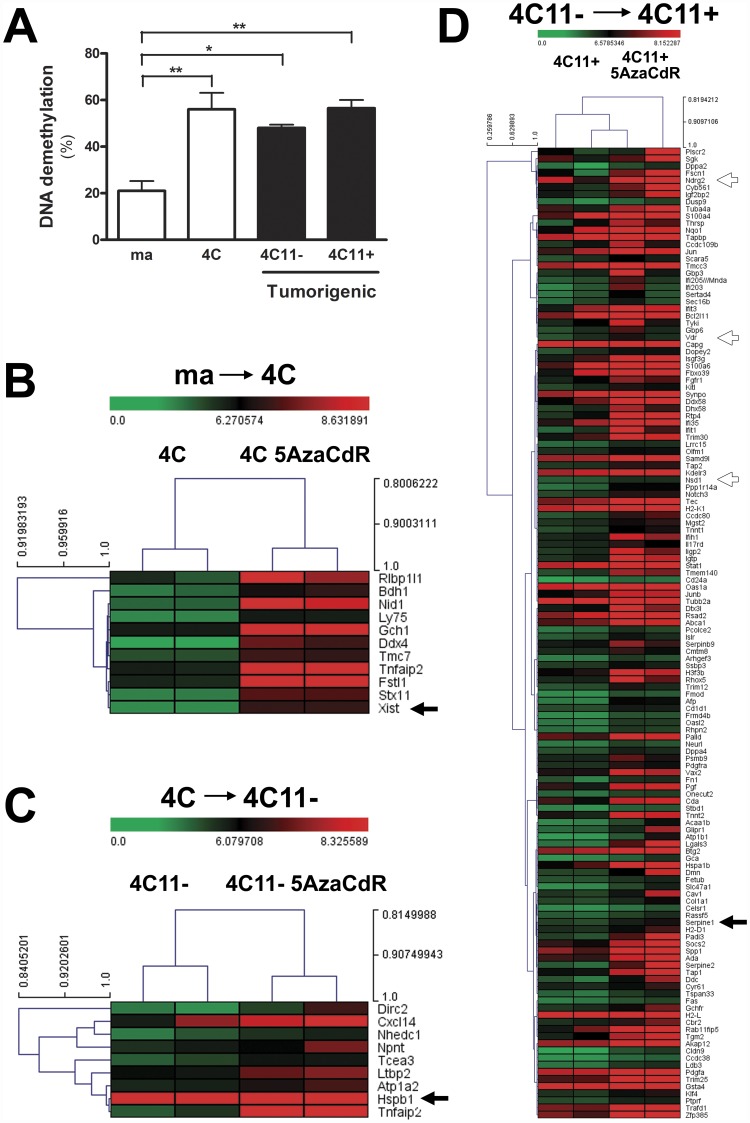
Demethylating Agent 5-aza-2′-deoxycytidine Differentially Modulated Gene Expression at Specific Stages of Melanoma Progression. (A) The 4C pre-malignant melanocytes, 4C11− non-metastatic and 4C11+ metastatic melanoma cell lines displayed the same percent of global genomic DNA demethylation upon 5AzaCdR treatment. (B-D) Genome-wide association studies of melan-a, 4C, 4C11− and 4C11+ cell lines untreated and previously exposed to 5AzaCdR revealed re-expressed genes at a given transition associated with melan-a malignant transformation. Heatmaps were generated by unsupervised hierarchical clustering using the Pearson correlation as metric distance and average-linkage as algorithm as well. The saturation of either color (scale from green to red) reflects the magnitude of the difference on gene expression level. ma: immortalized, but non-tumorigenic, melan-a melanocyte lineage; 4C: pre-malignant melanocyte lineage; 4C11−: non-metastatic melanoma cell line, and 4C11+: metastatic melanoma cell line. 5AzaCdR: 5-aza-2′-deoxycytidine.

Previously, we reported that the differential expression of Dnmt1 and Dnmt3a at protein level were associated with melan-a malignant transformation. Additionally, we demonstrated that the treatment with 10 µM 5-aza-2′-deoxycytidine (5AzaCdR) for 48 hours was able to inhibit the tumorigenicity capacity of the 4C11+ metastatic melanoma cell line *in vivo* [11]. Besides that, a growing body of data has shown that epigenetic modifier drugs can affect a number of tumor cell phenotypes, including the proliferation of melanoma cells [18], [19]. As the first step in characterizing the global demethylation responses induced by DNA hypomethylating agents, melan-a, 4C, 4C11− and 4C11+ cell lines were treated with 10 µM 5AzaCdR for 48 hours. The establishment of this treatment condition for 5AzaCdR was justified since we aimed to maximize demethylation efficiency and to minimize drug toxicity as well. Cell toxicity and viability were monitored by using a standard methyl thiazol tetrazolium assay (MTT), as previously described [11]. Following treatment of cells, there was a substantial decrease of genomic DNA methylation, since all treated cells showed significant percent of demethylation when compared with untreated cells. The decrease in global DNA methylation was observed at the same extent in 4C pre-malignant melanocytes, 4C11− non-metastatic, and 4C11+ metastatic melanoma cells as well (**Figure 3A**). In order to identify candidate genes targeted, either directly or indirectly, by aberrant DNA methylation at specific stages of melanoma progression, a genome-wide screening was performed in melan-a, 4C, 4C11− and 4C11+ cell lines after their treatment with 5AzaCdR at the time and concentration indicated. The high-throughput Affymetrix microarray approach was used as a platform screening, as described above. The experiments were carried out in two biological replicates. Class-comparison experiments were performed, and transcripts were selected as statistically significant by the pairwise two-class SAM analysis (FDR and *Q*-values<0.05) by using 500 random permutations as follows: I) gene expression profile of the parental melan-a melanocyte lineage versus its 5AzaCdR-treated counterpart cell: 250 up-regulated genes upon 5AzaCdR treatment; II) gene expression profile of 4C pre-malignant melanocyte lineage versus its 5AzaCdR-treated counterpart cell: 81 up-regulated genes upon 5AzaCdR treatment; III) gene expression profile of 4C11− non-metastatic melanoma cell line versus its 5AzaCdR-treated counterpart cell: 569 up-regulated genes upon 5AzaCdR treatment; IV) gene expression profile of 4C11+ metastatic melanoma cell line versus its 5AzaCdR-treated counterpart cell: 359 up-regulated genes upon 5AzaCdR treatment (**Figure S2, Tables S1–S4).** To identify candidate tumor suppressor genes whose expression could be modulated by epigenetic agents in distinct phases of melan-a malignant transformation, we performed genome-wide association studies for melan-a versus 4C, 4C versus 4C11−, and 4C11− versus 4C11+ cell lines as follows: I) intersection between genes found significantly down-regulation in 4C pre-malignant melanocytes in comparison to melan-a melanocyte lineage, and our list of over-expressed genes upon 5AzaCdR treatment in 4C melanocytes; II) intersection between genes found significantly down-regulation in 4C11− non-metastatic melanoma cell line in comparison to 4C pre-malignant melanocyte lineage, and our list of over-expressed genes upon 5AzaCdR treatment in 4C11− melanoma cell; III) intersection between genes found significantly down-regulation in 4C11+ metastatic melanoma cell line in comparison to 4C11− non-metastatic melanoma cell, and our list of over-expressed genes upon 5AzaCdR treatment in 4C11+ melanoma cell (**Figure 2A**, **Tables S1**–**S4**). Finally, the resulting genes were plotted in heatmaps (Pearson correlation metric distance; average linkage algorithm) organized according to the sequential stages of melan-a malignant transformation (**Figure 3B**–**3D**). In total, 11 genes showed responsiveness to 5AzaCdR treatment at stage I (**Figure 3B**), 9 genes at stage II (**Figure 3C**), and 136 genes at stage III (**Figure 3D**) of melanoma progression. Three genes were selected for further validation of gene expression (*Xist*, *Hspb1* and *Serpine1*) (**Figure 3B**–**3D**). These genes, except for *Hspb1* [18], were not previously reported to be modulated by 5AzaCdR treatment in melanoma cells. Despite the fact that the genes *Xist* and *Hspb1* were found deregulated earlier in the process of malignant transformation of melan-a melanocytes, studies have shown they are likely to have distinct functional roles in the tumor progression [20]–[22].

### Candidate Markers for melan-a Malignant Transformation

**Figure 4:**
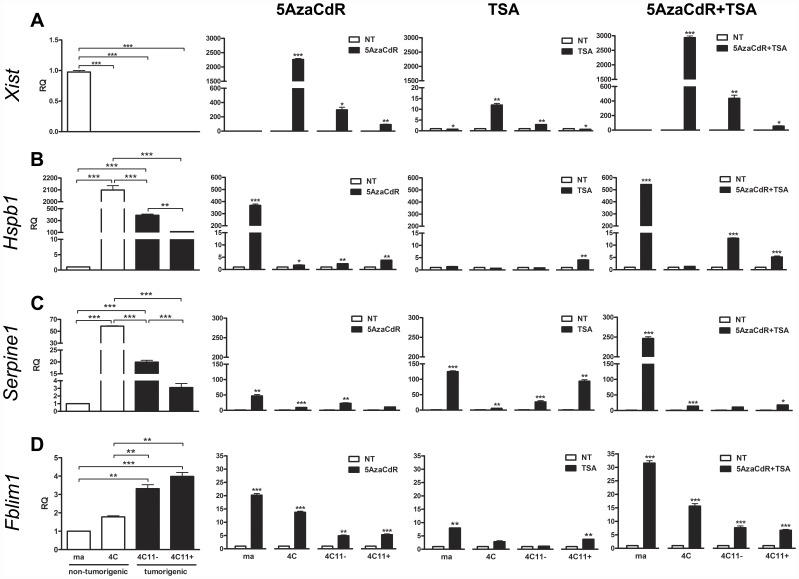
Candidate Markers for melan-a Malignant Transformation. (**A**) *Xist*, (**B**) *Hspb1*, (**C**) *Serpine1* and (**D**) *Fblim1* genes were assessed for relative quantification (RQ) in mouse cell lines across the melanoma progression spectrum (melan-a, 4C, 4C11− and 4C11+) non-treated (NT) or previously treated with 40 nM TSA for 16 hours, 10 µM 5AzaCdR for 48 hours, and 10 µM 5AzaCdR for 48 hours plus 40 nM TSA for 16 hours. Relative gene expression was calculated according to *2^-ΔΔCq^* method, using cellular Actb mRNA levels as endogenous reference control. Error bars in all cases represent SEM and *P* values were based on Students' *t* test or One-Way ANOVA test followed by the post-hoc Tukey. *P<0.05, **P<0.01, ***P<0.001. ma: immortalized, but non-tumorigenic, melan-a melanocyte lineage; 4C: pre-malignant melanocyte lineage; 4C11−: non-metastatic melanoma cell line, and 4C11+: metastatic melanoma cell line. TSA: Trichostatin A; 5AzaCdR: 5-aza-2′-deoxycytidine.

In recent years, it became evident that HDAC inhibitors such as Trichostatin A (TSA) in combination to DNA demethylation agents such as 5AzaCdR are attractive epigenetic agents to synergistically increase the expression of tumor silenced-genes in cancer [13], [23]–[25]. Along this line of evidence, the differential expression of selected genes were validated using quantitative real-time approach (RT-qPCR) in melan-a, 4C, 4C11− and 4C11+ cell lines previously exposed to three protocols based on epigenetic drug therapy: 40 nM TSA for 16 hours, 10 µM 5AzaCdR for 48 hours, and 10 µM 5AzaCdR for 48 hours plus 40 nM TSA for 16 hours (**Figure 4**). The rationale for the establishment of these treatment conditions for TSA and 5AzaCdR combined with TSA was based on the same explanation as those for 5AzaCdR alone, but also exploring, directly or indirectly, the acetylated state of genes. Cytotoxicity and cell viability were monitored by MTT assays, as previously described [11]. RT-qPCR revealed that the expression of *Xist* was negative in all melan-a-derived cell lines compared to melan-a melanocytes. The treatment of the 4C pre-malignant melanocyte as well as 4C11− non-metastatic melanoma cell line with 5AzaCdR plus TSA resulted in the highest up-regulation of *Xist* expression. On the other hand, we observed a more robust up-regulation of *Xist* gene expression when 5AzaCdR alone was given to 4C11+ metastatic melanoma cells. Furthermore, a slight decrease in the relative intensity of *Xist* gene expression was noted when TSA alone was given to melan-a melanocytes as well as 4C11+ metastatic melanoma cell line (**Figure 4A**). The dynamic of *Hspb1* gene expression was characterized by a significant enrichment found at pre-malignant and non-metastatic stages (4C melanocyte lineage and 4C11− melanoma cell line, respectively) in relation to melan-a melanocytes, followed by a progressive loss in *Hspb1* expression in 4C11+ metastatic melanoma cell line when compared with 4C pre-malignant melanocyte and 4C11− non-metastatic melanoma cell. The treatment of the 4C11− non-metastatic melanoma cell line with TSA resulted in no significant change of Hspb1 mRNA expression. Despite this fact, the highest up-regulation of *Hspb1* expression was induced by the combination of 5AzaCdR and TSA (> 12 times increase). At metastatic stage (4C11+ melanoma cell line), treatment with either TSA or 5AzaCdR was able to enhance *Hspb1* expression, but when TSA and 5AzaCdR were combined, a slight increase in the relative intensity of *Hspb1* gene expression was noted above individual drugs. As a whole, there was no significant change in *Hspb1* expression at pre-malignant stage (4C melanocyte lineage), except a slight increase when 5AzaCdR treatment was done. At immortalized, but non-tumorigenic stage (melan-a melanocytes), we observed a more robust up-regulation of *Hspb1* gene expression by 5AzaCdR and TSA combination than by 5AzaCdR alone (**Figure 4B**). *Serpine1* gene expression was significantly higher in the initial phases of melanoma genesis (4C pre-malignant melanocyte and 4C11− non-metastatic melanoma cell line) than immortalized melanocyte lineage (melan-a). On the other side, a progressive loss of *Serpine1* expression was found in 4C11+ metastatic melanoma cell line when compared with 4C pre-malignant melanocyte lineage and 4C11− non-metastatic melanoma cells. Although *Serpine1* expression was significantly higher in melan-a melanocyte lineage treated with 5AzaCdR and TSA, a meticulous dynamic in its expression was observed across the therapeutic spectrum. The 4C pre-malignant melanocyte lineage showed a progressive enhancement of *Serpine1* gene expression as follows: TSA alone (> 5), 5AzaCdR alone (> 9), and 5AzaCdR plus TSA (> 14 times increase) treatment. These results showed a synergistic effect of epigenetic agents to increase the expression of *Serpine1* gene at pre-malignant stage. There was no significant difference in Serpine1 mRNA levels in previously untreated 4C11− non-metastatic melanoma cell line relative to its 5AzaCdR+TSA-treated counterpart cell. When 4C11− melanoma cell was treated with TSA alone, the increase in *Serpine1* gene expression was higher than 5AzaCdR alone (at least 22 times increase). Besides that, 4C11+ metastatic melanoma cell line showed a striking enhancement of *Serpine1* gene expression (> 93 fold enrichment) when TSA alone was given, indicating TSA as the most effective epigenetic compound for *Serpine1* up-regulation at metastatic stage. Noteworthy, such enhancement of *Serpine1* at mRNA level returned close to basal level observed in previously untreated 4C11+ metastatic melanoma cell line when 5AzaCdR alone or in combination to TSA was given (> 10 and > 17 fold enrichment, respectively) (**Figure 4C**). We also found a set of differentially up-regulated genes in 4C11+ metastatic melanoma cell line in relation to its 4C11− non-metastatic counterpart cell (**Figure** **2A**). Curiously, some of them displayed significantly up-regulation upon 5AzaCdR treatment in 4C11+ melanoma cell such as *Fblim1* (**Table S4**), a cytoskeleton regulator that can control integrin activation and cancer progression [26]. Along this line, an additional transcript was validated by RT-qPCR as a candidate oncogene aberrantly over-expressed in 4C11+ metastatic melanoma cell line. As a result, we found a significant enrichment in *Fblim1* gene expression in 4C11− and 4C11+ melanoma cell lines when compared with melan-a melanocyte lineage. The 4C11− non-metastatic melanoma cell line showed no significant change in *Fblim1* expression when treated with TSA alone. On the other hand, approximately five times increase in *Fblim1* expression was observed after 5AzaCdR treatment, and > 7.5 times increase when 5AzaCdR combined with TSA was given. Besides that, the 4C11+ metastatic melanoma cell line showed a progressive enhancement of *Fblim1* gene expression as follows: TSA alone (> 3.5), 5AzaCdR alone (> 5), and 5AzaCdR plus TSA (> 6.5 times increase) treatment. The highest up-regulation of *Fblim1* expression was induced by the combination of 5AzaCdR and TSA in melan-a melanocytes. At pre-malignant stage (4C melanocyte lineage) we observed no significant change in *Fblim1* expression when TSA treatment was done. On the other side, when 5AzaCdR treatment was given alone or in combination to TSA, a significant up-regulation of *Fblim1* expression was found. Therefore, the treatment with 5AzaCdR alone or in combination to TSA showed a great potential to up-regulated *Fblim1* gene in cell lines with the lowest *Fblim1* expression (melan-a and 4C melanocytes) (**Figure 4D**). Despite the fact that RT-qPCR approach was more sensitive than microarray-based gene expression studies, there was a good correlation between mRNA profiles detected by microarrays and RT-qPCR techniques for the novel candidate markers implicated in malignant transformation of melan-a melanocytes.

### Demethylating Agent 5AzaCdR Combined with the HDAC Inhibitor TSA Modulate Cell Morphology and Gene Expression in Human Primary Melanocytes

**Figure 5:**
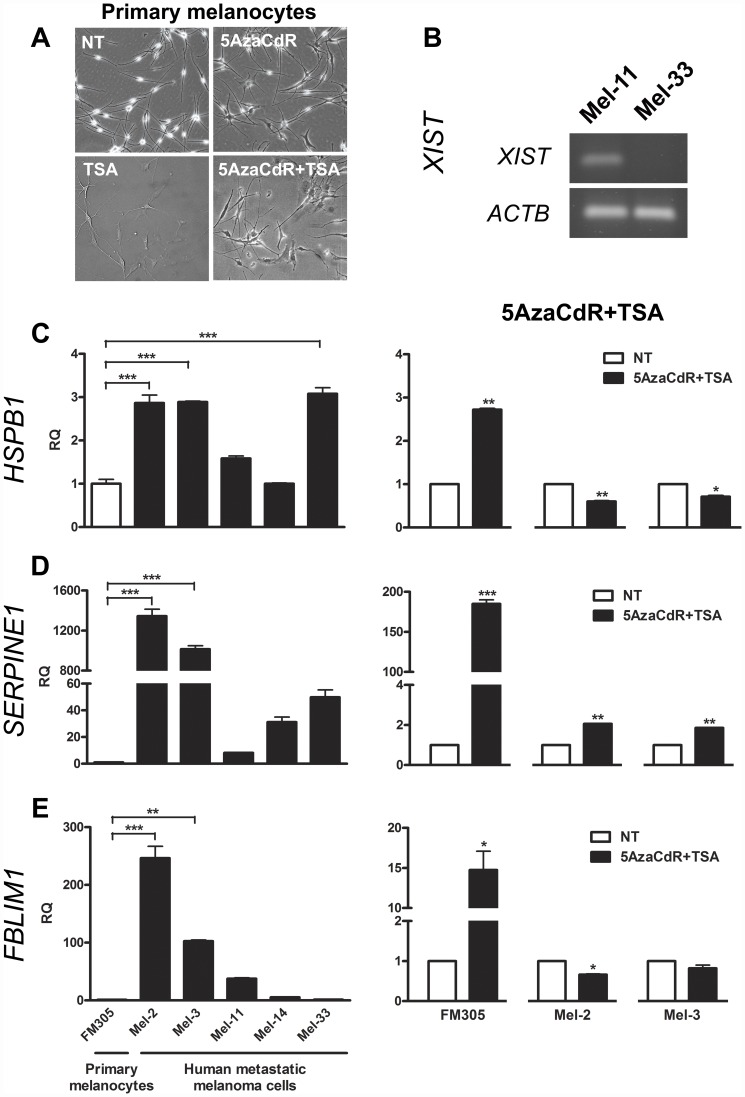
Epigenetic Flexibility in Human Specimens. (**A**) Changes on cell morphology of human primary melanocytes after their exposure to epigenetic modifier drugs, specially the combined therapy between 5AzaCdR and TSA. (**B**) Differential expression of *XIST* gene between two female patient-derived metastatic melanoma cell lines. (**C-E**) Expression of genes *HSPB1*, *SERPINE1* and *FBLIM1* was modulated in response to epigenetic compounds in human primary melanocytes and metastatic melanoma cells. Differential expression in a panel of five early-passage patient-derived metastases was also determined by RT-qPCR. Relative gene expression was calculated according to *2^-ΔΔCq^* method, using cellular ACTB mRNA levels as endogenous reference control. Error bars in all cases represent SEM and *P* values were based on Students' *t* test or One-Way ANOVA test followed by the post-hoc Tukey. *P<0.05, **P<0.01, ***P<0.001. FM305: human primary melanocytes from neonatal foreskin; Mel-2: right supraclavicular lymph node metastasis; Mel-3: counterpart of Mel-2 after the re-occurrence of the disease; Mel-11: brain metastasis; Mel-14: axillary lymph node metastasis, and Mel-33: inguinal lymph node metastasis. TSA: Trichostatin A; 5AzaCdR: 5-aza-2′-deoxycytidine.

To better follow up the steps of melanoma progression, we first examined the effect of TSA, 5AzaCdR, and 5AzaCdR plus TSA treatments on morphology of FM305 human primary melanocytes. The primary melanocytes exhibited the highest change on cell morphology when treated with 5AzaCdR in combination to TSA (**Figure 5A**). Based on this, we next investigated whether the differentially expressed genes validated as candidate markers for melan-a malignant transformation could respond to epigenetic compounds in human specimens. To do this, *HSPB1*, *SERPINE1* and *FBLIM1* genes were further validated in two patient-derived right supraclavicular lymph node metastases (Mel-2 and Mel-3 cell lines) previously exposed to 10 µM 5AzaCdR for 48 hours followed by 40 nM TSA for 16 hours. Interestingly, we observed the same profile of drug responsiveness for FM305 human primary melanocytes (**Figure 5C-5E**) and melan-a mouse melanocyte lineage (**Figure 4B-4D**) in relation to the expression of genes *HSPB1*/*Hspb1*, *SERPINE1*/*Serpine1*, and *FBLIM1*/*Fblim1*. In spite of this, only the *SERPINE1*/*Serpine1* gene showed the same treatment responsiveness when 5AzaCdR combined with TSA was given to 4C11+ mouse metastatic melanoma cell line as well as Mel-2 and Mel-3 human metastatic melanoma cells. The treatment conditions for all human cells were the same as those described for mouse cell lines, and cell viability was monitored by the Trypan Blue assays (data not shown). Furthermore, we investigated the differential expression of genes *HSPB1*, *SERPINE1* and *FBLIM1* in a panel of five patient-derived metastatic melanoma cell lines in comparison to FM305 primary melanocytes. Based on the clinical staging criterion measured by the time from post treatment of primary tumors to re-occurrence of the diseases, we have distinguished two groups of metastases: M1 (Mel-2 and Mel-3: better prognosis), and M2 (Mel-11, Mel-14 and Mel-33: worst prognosis). RT-qPCR approach revealed a unifying profile of gene expression in which *FBLIM1*, *HSPB1* and *SERPINE1* up-regulation significantly distinguishes less aggressive metastases (Mel-2 and Mel-3) from those displaying more aggressiveness (Mel-11 and Mel-14) (**Figure 5C-5E**). Curiously, the expression profile exhibited for the genes *HSPB1* and *SERPINE1* in Mel-2 and Mel-3 human metastases was noted for mouse cell lines representing pre-malignant melanocytes and non-metastatic melanoma cells (4C and 4C11− cell lines, respectively) in comparison to melan-a melanocytes (**Figure 4B-4C**, **Figure 5C-5D**). Additionally, we investigated by RT-PCR the expression of *XIST* in two female patient-derived metastatic melanoma cell lines with high degree of aggressiveness (Mel-11 and Mel-33). The positive correlation on expression of *Xist*/*XIST* was seen for 4C11+ mouse metastatic melanoma cell line and Mel-33 (inguinal lymph node metastasis), but not for Mel-11 (brain metastasis) (**Figure 4A** **,** **Figure 5B**).

### Treatment of Human Melanocytic Cells with Epigenetic Compounds Modulate the Expression of Genes Recognized by Affecting Melanocyte Biology and Response to Cancer Therapy

**Figure 6:**
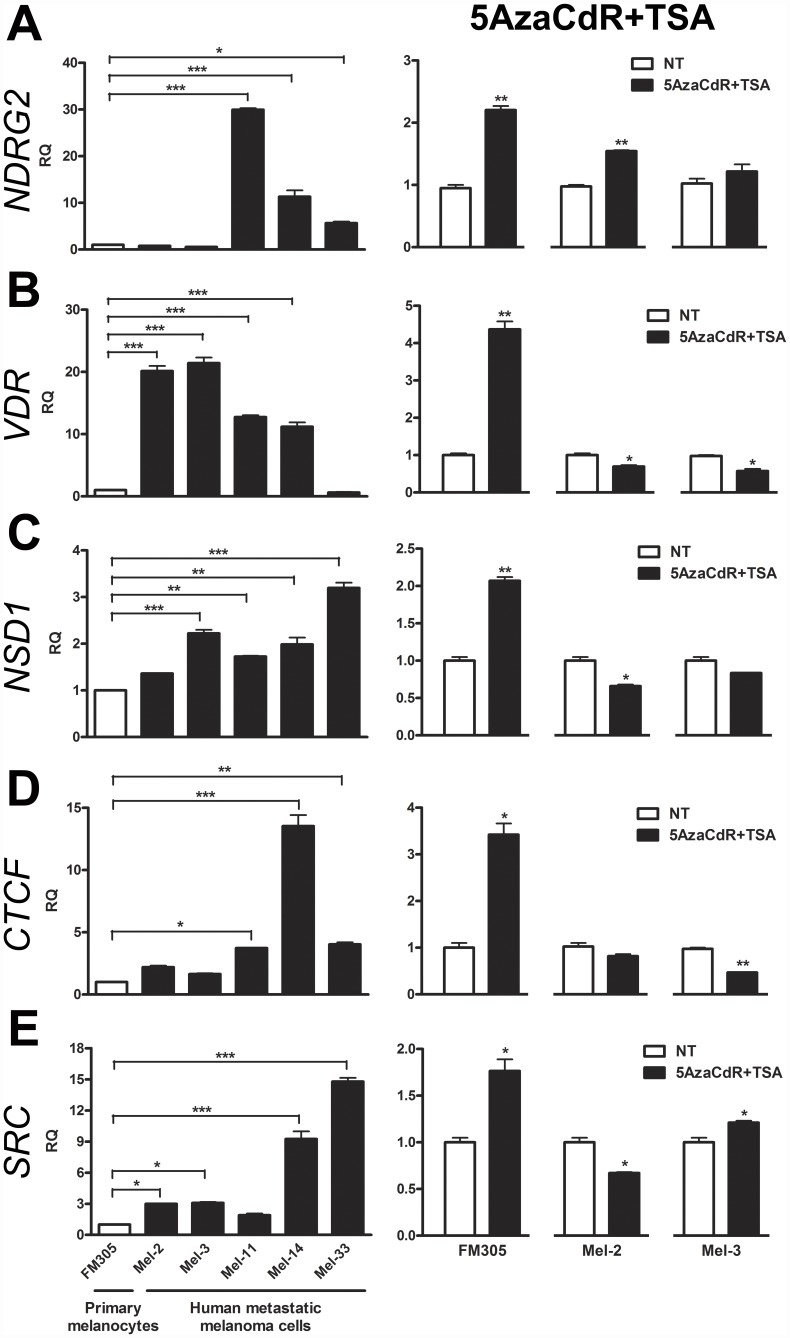
Epigenetic Drugs Modulated the Expression of Genes Recognized by Affecting Melanocyte Biology and Response to Therapy. Expression of genes recognized by affecting (A-B) melanocyte biology, and (C-E) response to cancer therapy was deregulated in a panel of five patient-derived metastatic melanoma cell lines in comparison to FM305 primary melanocytes, as assessed by RT-qPCR approach. (A-E) Epigenetic compounds 5AzaCdR and TSA modulated the expression of genes *NDRG2*, *VDR*, *NSD1*, *CTCF* and *SRC* in normal melanocytes and metastatic melanoma cells. Relative gene expression and Statistics were calculated as described in figure 5. FM305: human primary melanocytes from neonatal foreskin; Mel-2: right supraclavicular lymph node metastasis; Mel-3: counterpart of Mel-2 after the re-occurrence of the disease; Mel-11: brain metastasis; Mel-14: axillary lymph node metastasis, and Mel-33: inguinal lymph node metastasis. TSA: Trichostatin A; 5AzaCdR: 5-aza-2′-deoxycytidine.

As the first step in identifying genes implicated in melanocyte biology, whose expression were deregulated in the progression of melanomas, we performed a manual annotation of the 136 genes significantly down-regulated in the transition from 4C11− to 4C11+, and up-regulated in 4C11+ metastatic melanoma cells upon 5AzaCdR treatment. Two genes were selected for validation of gene expression (*NDRG2* and *VDR*) (**Figure 3D**) [27]–[30]. Moreover, we were also interested in identifying genes potentially implicated in response to cancer therapy. Along this line, we selected the gene *NSD1* (**Figure 3D**) [31] as well as two additional genes based on the literature searched for further validation (*CTCF* and *SRC*) [32], [33]. Therefore, the expression pattern of human *NDRG2*, *VDR*, *NSD1*, *CTCF* and *SRC* genes in several human metastatic melanoma cell lines in comparison to primary melanocytes were analyzed at mRNA level by RT-qPCR methodology. The highest expression level of *NDRG2* gene was found in the brain (Mel-11), axillary lymph node (Mel-14), and inguinal lymph node (Mel-33) metastases. On the other hand, *NDRG2* mRNA was nearly undetectable in the two right supraclavicular lymph node metastases derivatives from the same patient before and after the re-occurrence of the disease (Mel-2 and Mel-3 cell lines, respectively). Interestingly, when 5AzaCdR treatment was given in combination to TSA, a significant up-regulation of *NDRG2* expression was found in Mel-2 metastatic melanoma cells (**Figure 6A**). *VDR* gene expression was highly positive in the metastases examined, except for Mel-33 cell line, which was established from inguinal lymph node metastasis. A slight decrease on *VDR* gene expression was observed when 5AzaCdR plus TSA treatment was given to Mel-2 and Mel-3 melanoma cells (**Figure 6B**). The significant up-regulation of human *NSD1* gene was found in all metastases evaluated, except Mel-2, in comparison to primary melanocytes. The treatment based on 5AzaCdR plus TSA caused a slight decrease on *NSD1* gene expression in Mel-2 melanoma cells (**Figure 6C**). An enhancement of *CTCF* gene expression was specifically found in the aggressive metastases examined (Mel-11, Mel-14 and Mel-33) in comparison to primary melanocytes. Mel-3 melanoma cells showed a reduced expression on *CTCF* gene upon 5AzaCdR combined with TSA treatment (**Figure 6D**). The *SRC* gene expression was higher in the more aggressive metastases (Mel-14 and Mel-33) when compared to those presenting less aggressiveness (Mel-2 and Mel-3). Interestingly, the expression of *SRC* gene had undergone down- and up-regulation, respectively, when Mel-2 and Mel-3 melanoma cells were treated with 5AzaCdR combined with TSA (**Figure 6E**). All genes evaluated had undergone up-regulation in FM305 primary melanocytes upon 5AzaCdR+TSA treatment (**Figure 6A-6E**).

### Hierarchical Clustering and Network Analyses Revealed Novel Gene Interactions in Melanoma Progression

**Figure 7:**
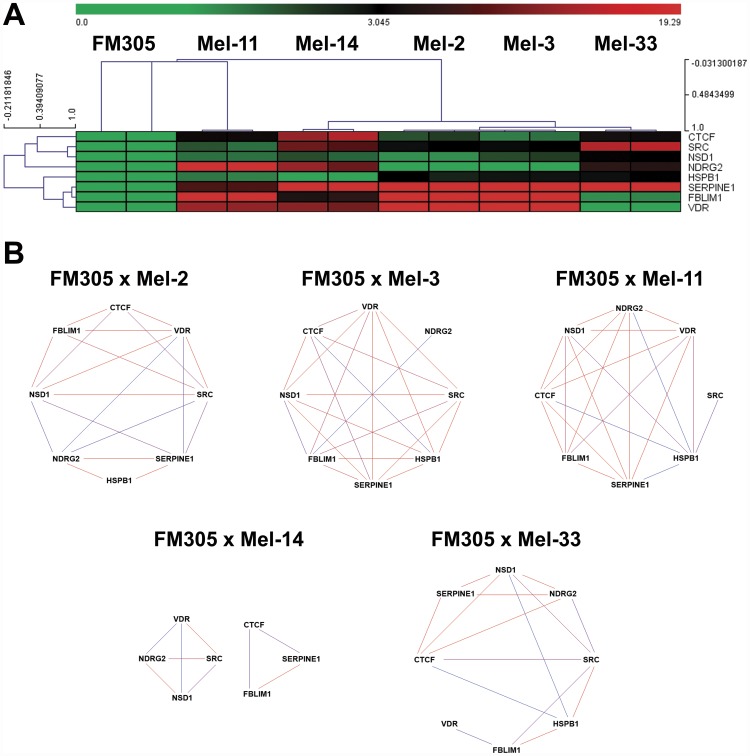
Hierarchical Clustering and Network Analyses Revealed Novel Gene Interactions in Melanoma Progression. (**A**) Heatmap of five patient-derived metastatic melanoma cell lines. Metastases of human melanoma cell lines were clustered according to the expression profiles of genes *CTCF*, *FBLIM1*, *HSPB1, NDRG2, NSD1, SERPINE1, SRC* and *VDR*. Heatmaps were generated by unsupervised hierarchical clustering using the Pearson correlation as metric distance and average-linkage as algorithm as well. The saturation of either color (scale from green to red) reflects the magnitude of the difference on gene expression level. (**B**) Despite the molecular heterogenety observed in melanoma cells, relevance networks pointed out gene interactions commonly observed among the metastases examined, such as *NDRG2*/*NSD1*, *VDR*/*NSD1*, *SRC*/*NSD1*, *CTCF*/*SERPINE1*, *HSPB1*/*SERPINE1* and *FBLIM1*/*SERPINE1*. FM305: human primary melanocytes from neonatal foreskin; Mel-2: right supraclavicular lymph node metastasis; Mel-3: counterpart of Mel-2 after the re-occurrence of the disease; Mel-11: brain metastasis; Mel-14: axillary lymph node metastasis, and Mel-33: inguinal lymph node metastasis.

In an effort to elucidate the possible usefulness of the novel gene expression signature as a marker of melanoma progression, we performed an unsupervised hierarchical clustering (HCL) analysis by grouping the five human metastatic melanoma cell lines examined according to their patterns of gene expression (Pearson correlation metric distance; average linkage algorithm). To do this, mRNA levels of *HSPB1*, *SERPINE1*, *FBLIM1*, *NDRG2*, *VDR*, *NSD1*, *CTCF* and *SRC* were measured after RT-qPCR approach by using the *2^-ΔΔCq^* method, and plotted in a heatmap. Notably, unsupervised HCL of patient-derived metastatic melanoma cell lines was able to cluster samples into their theoretical cell type branches (**Figure 7A**). Finally, Relevance Networks were generated allowing us to find, by an additional technique, some strong gene regulatory relationships as candidate markers in advanced melanomas. In such methodology, each pair of genes related by a correlation coefficient larger than a minimum threshold and smaller than a maximum threshold is connected by a line. Links colored in red represent genes that are positively correlated while links colored in blue represent genes negatively correlated. To illustrate some findings, the relationships between pairs of genes *NDRG2*/*NSD1, VDR*/*NSD1*, *SRC*/*NSD1* as well as *CTCF*/*SERPINE1* were deregulated in four of five metastases examined. Additionally, interactions between pairs of genes *HSPB1*/*SERPINE1* as well as *FBLIM1*/*SERPINE1* were found in three of five metastases evaluated. These results highlight the potential usefulness of the novel gene expression signature as a marker in melanoma progression (**Figure 7B**).

## Discussion

Up to date, classical chemotherapy as well as discovered biomarkers have not been significant successful neither to diagnosis melanomas or to benefit patients, with rare exceptions, since only the gene *CTLA4* and V600E mutational status of *BRAF* gene have been recognized as bona fide targets to treat metastatic disease [34], [35].

In this study, we used a mouse model of melanoma progression in which the non-tumorigenic melan-a melanocyte lineage was submitted from anchorage-dependent to anchorage-independent culture conditions [15]. We reported that melan-a melanocytes acquired features of aggressive melanoma cells after sequential detachment/re-adhesion cycles. This aggressive phenotype was characterized by a biological relationship between the complex karyotype with Robertsonian fusions and metastatic capacity [16] of 4C11+ melanoma cells *in vivo* (**Figure 1B-1C**, **Table 1**, **Figure S1**).

Genome-wide screening of melan-a, 4C, 4C11− and 4C11+ cell lines revealed a large set of differentially up- and down-regulated genes in the consecutive transitions implicated in melan-a malignant transformation (from non-tumorigenic to metastatic stage) (**Figure 2A**). It is well known that mammalian development and cell type determination result from the tightly controlled regulatory mechanisms between signaling pathways and transcription factors in progenitor cells as well as environmental influences. Interestingly, we showed that the conversion from melan-a melanocytes to 4C pre-malignant melanocytes involved the down-regulation of neuroendocrine pathways [36], [37], providing evidence that stress-driven changes in the environment can impair the neuroendocrine activity of the melanocytes in the initial stages of melan-a tumorigenic conversion. Additionally, we showed that the conversion from 4C pre-malignant melanocytes to 4C11+ metastatic melanoma cells involved, among others, the down-regulation on the expression of melanocyte stem cell-related signaling pathways, such as TGF-β [38] and Notch [39] (**Figure 2B, 2D**). This finding is in agreement with the data reported earlier that melan-a melanocytes and 4C11+ metastatic melanoma cells display an epithelial morphology, whereas 4C pre-malignant melanocytes and 4C11− non-metastatic melanoma cells exhibit a mesenchymal morphology with enhanced expression of the gene *Chd1*, and the presence of the repressive histone mark H3K27 trimethylation plus the active mark H3K4 trimethylation [11]. Taken together, these findings would indicate a less differentiated chromatin state. In terms of human cancer biology, the stem cell chromatin state may enhance the likelihood for DNA hypermethylation on tumor suppressor-coding genes [40]. Based on the shared genetic background of the cells as well as commonly perturbed pathways over-represented in 4C pre-malignant melanocytes in relation to melan-a cells, and under-represented in 4C11+ metastatic melanoma in relation to 4C11− non-metastatic melanoma cells (**Figure 2D**), our rationale was that epigenetic reprogramming on gene expression occurring earlier in melan-a malignant transformation would be useful to predict a phenotype associated with metastasis.

Using genome-wide expression arrays we identified patterns of gene expression following 5-aza-2′-deoxycytidine (5AzaCdR) exposure. Genome-wide association studies between untreated cell lines and their 5AzaCdR-treated counterpart cells allowed us to identify candidate down-regulated genes whose expression could be modulated, directly or indirectly, by 5AzaCdR treatment at specific stages of melanoma progression (**Figure 3B-3D**). A gene expression signature was discovered and validated by RT-qPCR in melan-a, 4C, 4C11− and 4C11+ mouse cell lines (**Figure 4A-4C**). Moreover, candidate metastasis-related biomarkers were validated in early-passage cultures of patient-derived metastatic melanoma cell lines as well as primary melanocytes untreated and/or previously treated with 5AzaCdR in combination to the HDAC inhibitor Trichostatin A (TSA) (**Figure 5B-5E**, **Figure 6**).

A signature comprised by the genes *Xist*/*XIST*, *Hspb1*/*HSPB1*, *Serpine1*/*SERPINE1*, *Fblim1*/*FBLIM1*, *CTCF*, *NDRG2*, *NSD1*, *SRC* and *VDR* was selected for further validation by RT-qPCR approach. *XIST* (X inactive-specific transcript) is the master regulator of X-chromosome inactivation to equalize gene products between male and female mammals, by inactivating one of the X-chromosomes during the early embryonic development in females (Lyon hypothesis, 1962) [41]. Apart from this, DNA copy number changes of *XIST* gene was reported in colorectal carcinomas [22]. The positive correlation between *HSPB1* (heat shock 27 KDa protein 1) and *SERPINE1* (serpin peptidase inhibitor, clade E, member 1) overexpression induced reversal of the invasive and metastatic phenotype of melanoma cells *in vitro* [20], [21]. The positive correlation between *FBLIM1* (filamin binding LIM protein 1) and *SRC* (v-src sarcoma viral oncogene homolog [avian]) overexpression resulted in *anoikis* resistance contributing to tumorigenicity of established carcinoma cells [42]. Additionally, SRC activation was implicated with primary resistance to PLX4032 in patient-derived melanoma cells [33]. The protein encoded by the *CTCF* (CCCTC-binding factor) gene contributes to the organization of DNA into loops of transcriptionally repressive heterochromatin or into active euchromatin, which facilitates blocks or which connects distal enhancers and proximal promoters [12]. In terms of response to cancer therapy, the insulator protein CTCF mediated the down-regulation of telomerase in response to sulforaphane exposure, a compound that has histone deacetylase inhibition activity, in human breast cancer cells [32]. *NSD1* (nuclear receptor binding SET domain protein 1) gene silencing was associated with tamoxifen resistance in breast tumors [31]. The decrease on the expression of *VDR* (vitamin D receptor) gene was linked to progression of pigmented skin lesions as well as shorter patients' melanoma overall survival [30]. Finally, down-regulation of the *NDRG2* (NDRG family member 2) gene was reported to inhibit the metastatic potential of melanoma cells *in vitro* and *in vivo* [27], [28].

Even though much has been discussed about the discrepancies between mouse and human mechanisms of carcinogenesis, network analysis showed, for example, the interaction between pairs of genes *HSPB1*/*SERPINE1* as well as *FBLIM1*/*SERPINE1* in some of the human metastases examined (**Figure 7B**). Moreover, up-regulation on the expression of *Serpine1*/*SERPINE1* gene upon 5AzaCdR plus TSA treatment was found in 4C11+ mouse metastatic melanoma cells as well as Mel-2 and Mel-3 patient-derived metastases (**Figure 4C**, **Figure 5D**). In addition, melan-a mouse melanocyte lineage and FM305 human primary melanocytes shared the up-regulation on the expression of *HSPB1*, *SERPINE1* and *FBLIM1* genes when 5AzaCdR associated with TSA was given (**Figure 4B-4D**, **Figure 5C-5E**).

Hierarchical clustering of human metastatic melanoma cells was able to group samples into their theoretical cell type branches (**Figure 7A**). Despite the fact that it could be a marker of molecular heterogeneity, network analysis pointed out gene interactions commonly perturbed among the metastases evaluated such as *NDRG2*/*NSD1*, *VDR*/*NSD1*, *SRC*/*NSD1*, *CTCF*/*SERPINE1*, *HSPB1*/*SERPINE1* and *FBLIM1*/*SERPINE1* (**Figure 7B**). Interestingly, deregulation on the expression of epigenetic machinery components such as *CTCF* and the *NSD1* histone methyltransferase-coding genes was noted in human metastases (**Figure 6C-6D**, **Figure 7**). An epigenetic signature was also found deregulated in 4C11+ mouse metastatic melanoma in relation to 4C11− non-metastatic melanoma cells (**Figure 2E**). Together, these results could suggest the involvement of epigenetic abnormalities in melanoma progression. However, the regulatory mechanisms have yet to be determined. To illustrate some potential contribution of our findings in the context of melanoma progression, the interaction between the pair of genes *CTCF*/*SERPINE1* (**Figure 7B**) might impair *P53*-mediated senescence in melanomas even in the absence of direct genetic or epigenetic lesions. It is justified since *SERPINE1* gene is a critical downstream target of *P53* gene in the induction of replicative senescence [43]. Additionally, the *P53* gene promoter was reported to be regulated by the CTCF transcription factor in transformed cell lines [44]. In fact, evidence is emerging that epigenetic changes might play a significant role in tumor cell plasticity and the metastatic phenotype of melanoma cells [10].

Notably, FM305 human primary melanocytes were able to alter their morphology (**Figure 5A**) and gene expression (**Figure 5C-5E**, **Figure 6**) in response to 5AzaCdR plus TSA exposure. To our knowledge, this is the first report suggesting an epigenetic flexibility in normal primary melanocytes in response to alterations in their environment promoted by the exposure to epigenetic modifier drugs. If this ‘epigenetic flexibility’ can translate in plasticity and changes on cellular behavior will need to be carefully evaluated in the future.

Finally, accumulating evidences implicate the molecular coupling of differentiation program patterns and melanoma stem cells in the establishment of drug tolerance and response to melanoma therapy. In addition, resistance to apoptosis and the chromatin state of genes act as hallmarks of drug resistance [1], [45]–[48]. To identify mechanisms underlying successful melanoma therapy, some points need to be carefully discussed: i. This study was performed by using a mouse model of melanoma progression in which sequential detachment/re-adhesion cycles of non-tumorigenic melan-a melanocyte lineage caused cells to adopt 3D cluster morphology, a progressive *anoikis*-resistance phenotype [15], [49] and the acquisition of malignant phenotype and a metastatic behavior of 4C11+ melanoma cell line as well (**Figure 1**); ii. Melanoma stem cell markers are dynamically regulated in each transition associated with melan-a tumorigenic conversion (**Figure S3**). Noteworthy, Cheli and coworkers (2011) reported that the inhibition of microphthalmia-associated transcription factor (*MITF*), the master regulator of melanocyte lineage commitment and differentiation, increased the tumorigenic potential of human melanoma cells whereas up-regulated stem cell markers and enhanced the number of melanoma-initiating cells in human melanoma spheroids. Thus, authors concluded that MITF acts as the key molecular switch between human melanoma initiating cells and their differentiated progeny [48]. Melan-a, 4C, 4C11− and 4C11+ cell lines vary in their differentiation program patterns (**Figure S3**; Camila Ferreira de Souza, manuscript in preparation). Despite this fact, it is so premature to conclude that the spheroid cells found after the anchorage blockage of 4C pre-malignant melanocytes are cancer stem cells. Further studies need to be carefully conducted to address this issue. Such characterization might carry important clinical implications as cancer stem cells show increased resistance to chemotherapy; iii. The chromatin modifying enzymes such as increased JARID1A and/or increased histone deacetylases (HDACs) can reversibly mediate the acquisition of a drug tolerant state in melanomas [46]. Importantly, we previously reported that many histone marks differ among melan-a, 4C, 4C11− and 4C11+ cell lines, which could be associated with aberrant gene expression [11] (and data not shown). The notion that epigenetics is associated with widespread gene expression changes in cancer is not new. Some studies have pointed out that transcriptional factors, epigenetic complexes and pluripotency markers cooperate to modulate the expression of *XIST* gene. In addition, the presence of histone acetyltransferases (HATs) are related to the expression of *VDR* gene [50]–[53]. However, the relationship between them at a given stage of disease appears to be complex and certainly merits a careful exploration. Curiously, Do and coworkers (2008) showed an enhancement in Dnmt3a RNA levels after TSA treatment in female neurosphere cells suggesting that *Dnmt3a* gene expression is controlled by histone modification. Importantly, depletion of Dnmt3a mRNA was sufficient to inhibit the *de novo* methylation of the *Xist* region after TSA treatment, although Xist RNA level was slightly reduced. Together, these findings indicate that Dnmt3a has a direct effect on *Xist* methylation acting as a negative regulator of *Xist* gene as well [51]. We previously showed that the expression of Dnmt1 and Dnmt3a at protein level was detected in melan-a melanocytes. Besides that, the up-regulation of Dnmt3a protein was only found for 4C11+ metastatic melanoma cell line [11]. Interestingly, a slight decrease in the relative intensity of *Xist* gene expression was noted when TSA was only given to melan-a melanocytes and 4C11+ metastatic cells (**Figure 4A**). Thus, it is reasonable to hypothesize that no one molecular mechanism accounts for the properties of DNA methyltransferase and histone deacetylase inhibitors, even at a specific gene locus. It could explain, for example, others apparent opposite results, such as: i) The treatment of the 4C pre-malignant melanocytes and 4C11− non-metastatic melanoma cell line with 5AzaCdR plus TSA resulted in the highest up-regulation of *Xist* expression whereas a more robust up-regulation of *Xist* gene expression in 4C11+ metastatic melanoma cell line was observed when 5AzaCdR alone was given (**Figure 4A**); ii) The treatment of melan-a melanocytes as well as 4C11− non-metastatic and 4C11+ metastatic melanoma cell lines with 5AzaCdR in combination to TSA resulted in the highest up-regulation of *Hspb1* gene expression whereas no significant change was observed at pre-malignant stage (4C melanocyte lineage) except a slight increase when 5AzaCdR was done (**Figure 4B**); iii) *VDR* gene expression was up-regulated in FM305 primary melanocytes after combined 5AzaCdR and TSA treatment whereas a slight decrease on *VDR* gene expression was observed when the combinatorial treatment protocol was given to Mel-2 and Mel-3 patient-derived metastatic melanoma cells (**Figure 6B**). Integrating the regulatory information can support positive or negative control of gene expression through synergism and antagonism. Therefore, a major effort needs to be done to find multiple regulatory signals, including epigenetic complexes, differentially deregulated at several phases of melanoma progression. It could help find drugs that preferentially target cancer cells by exploring the effects of 5AzaCdR and/or TSA treatment and molecular heterogeneity in melanoma prevention.

To summing up, the current study demonstrates the utility of our murine melanoma progression model to successfully identify candidate biomarkers commonly perturbed in metastases. Furthermore, it provides a platform for testing new agents in combination therapies. Finally, the molecular markers deregulated earlier in melanoma development should be validated in the future in a large cohort of human specimens as a prognostic signature.

## Materials and Methods

### Model of Study

This work was done using an *in vitro* mouse melanoma model in which the deregulation of cellular adhesion configured the carcinogenic stimulus to melan-a (ma) melanocyte lineage. This experimental model developed by our research group was previously described [14], [15]. It does not use physical, chemical or environmental carcinogens or genetic manipulations, and can easily be reproduced to study mechanisms of melanoma genesis in a spectrum of distinct stages of tumor progression [15].

Melan-a, that is the first known established lineage of non-tumorigenic mouse melanocytes, is a spontaneous immortal line of pigmented melanocytes derived from normal epidermal melanoblasts from embryos of inbred C57BL mice. Melan-a cells grow in adherent condition and require 12-*O*-tetradecanoyl phorbol 13-acetate (PMA) to grow [14]. Recently, our group has showed no structural chromosomal abnormalities in melan-a cell line based on karyotype analysis [17]. Thus, melan-a melanocytes provide an excellent parallel non-tumorigenic lineage for studies of the molecular basis of melanoma pathophysiology.

Shortly, four sequential anchorage blockade cycles of melan-a melanocytes for 96 hours culminate with the establishment of the melan-a derived subline corresponding to a non-tumorigenic, but pre-malignant melanocyte lineage, which was called 4C. Apart from this intermediate phenotype associated with melanomas, distinct melanoma cell lines (4C11− and 4C11+, among others) were established by limiting dilution of spheroids found after a new anchorage blockade cycle of the 4C pre-malignant melanocyte lineage. To perform repetitive deadhesion cycles, melan-a melanocytes were plated at 1% agarose-coated plates, as previously described [15]. We have characterized the 4C as a pre-malignant melanocyte lineage since it presents a higher *anoikis* resistance [49] and an aberrant differentiation program (Camila Ferreira de Souza, manuscript in preparation) relative to its parental melan-a cell line. Therefore, we are using a time-dependent model of melanoma progression that was developed at five different time-points to carefully analyze the molecular basis of the tumorigenic conversion of melan-a melanocytes subjected to a sustained stress condition, which was promoted by consecutive detachment/re-adhesion cycles.

### Cell Culture

Mouse melan-a melanocyte lineage was established by Prof. Dorothy C. Bennett (Division of Biomedical Sciences, St. George's, University of London, UK) [14] and kindly provided by Prof. Michel Rabinovitch (Departamento de Parasitologia, Universidade Federal de São Paulo, Brazil). Melan-a melanocytes were grown in RPMI 1640 pH 6.9 medium (Gibco, Carlsbad, CA) supplemented with 10% fetal bovine serum (FBS; Gibco BRL, Grand Island, NY), 200 nM PMA (Sigma-Aldrich, Saint Louis, MO) and antibiotics (Gibco) at 37°C with 5% CO_2_ in air. Cell sublines derivatives from melan-a melanocytes after sequential detachment/re-adhesion cycles (4C, 4C11− and 4C11+) were grown under the same condition as the parental melan-a melanocyte lineage, except for PMA, since they lost the requirement of this factor to grow.

Human primary melanocytes from neonatal foreskin were isolated after informed consent signed by patients according to the Ethics Committee from Hospital Universitário (HU-USP, CEP 943/09) and kindly provided by Prof. Silvya S. Maria-Engler (Departamento de Análises Clínicas e Toxicológicas, Universidade de São Paulo, Brazil). These cells were maintained in basal medium for melanocytes 254CF supplemented with Human Melanocyte Growth Supplement (HMGS) and 0.2 mM calcium chloride (Gibco). Five short-term cultures of human melanoma cell lines representing lymph node and systemic metastases (Mel-2, Mel-3, Mel-11, Mel-14 and Mel-33) were patient-derived and maintained in RPMI 1640 pH 7.2 medium (Gibco) supplemented with 15% FBS (Gibco BRL), 2 mM L-glutamine, 1 mM sodium pyruvate and antibiotics (Gibco). These cell lines, freshly derived from surgery after informed consent signed by patients according to human subjects' research protocol approved by the Ethics Committee of the Fundação Antônio Prudente, Hospital A.C. Camargo (CEP 096/98, 31/03/98), were a kindly gift from Dr. Débora Castanheira Pereira da Silva (Hospital A.C. Camargo).

### Mice Studies

All *in vivo* studies were carried out in accordance with the protocol approved by the Institutional Animal Care and Ethics Committee of the Universidade Federal de São Paulo (CEP 1509/07 and 0389/09 from P.X. and C.F.S., respectively). Female syngeneic C57Bl/6 mice were obtained at 6-8 weeks of age from Biotério Central (Universidade Federal de São Paulo, Brazil). Animals were maintained on a 12 hours light/dark cycle and were given free access to food in accordance to the International Guiding Principles for Biomedical Research Involving Animals (CIOMS; Genebra, 1985).

Melan-a, 4C, 4C11− and 4C11+ cell lines were harvested after trypsin treatment of subconfluent monolayers, counted and suspended in phosphate-buffered saline (PBS). To perform the *in vivo* tumorigenesis assay, 2.5x10^5^ 4C11− and 4C11+ melanoma cells per mouse were inoculated subcutaneously. Animals were checked daily to monitor the presence of palpable tumors during 100 days. The growth tumor curves were determined by measuring the tumor volume (mm^3^) as follows: *d^2^*×*D/2*, in which *d* corresponds to the smallest tumor diameter and *D* to the largest one. To perform the *in vivo* experimental metastasis assay, 2.5x10^5^ 4C11− and 4C11+ melanoma cells per mouse were inoculated intravenously into lateral tail vein. Mice were sacrificed, lungs were surgically excised, and tumor multiplicities were evaluated by measuring the presence and the number of metastatic pulmonary foci. In both experiments, non-tumorigenic melan-a and 4C melanocyte lineages were used as negative controls. Each experimental group consisted of five animals. Statistical significance (*P*<0.05) was determined using One-way ANOVA test. Mice survival curves after intravenously inoculation of 4C11− and 4C11+ melanoma cells were calculated by the Kaplan-Meier method, with the statistical significance (*P*<0.05) evaluated by the Log-rank test. All statistics were carried out using GraphPad Prism software version 5 for Windows (GraphPad, San Diego, CA).

### Spectral Karyotyping Analysis (SKY)

In order to investigate structural chromosomal abnormalities and rearrangements as indicators of karyotype evolution in melanomas, SKY was performed using the ASI (Applied Spectral Imaging, Vista, CA) kit for mice. Chromosome preparations were hybridized and stained in accordance to manufacturer's instructions. Imaging of the signals was carried out using the Spectra Cube (ASI) system mounted on an Axioplan 2 microscope (Carl Zeiss) with a 63x/1.4 oil objective and the Case Data Manager 4.0 software (ASI). The chromosomes were counterstained with 4,6-diamino-2-phenylindole (DAPI) in an anti-fade solution (ASI). Twenty metaphases were evaluated per cell line examined. Statistical significance (*P*<0.05) was calculated by the Kruskal-Wallis test followed by the post-hoc Dunn's. All statistics were carried out using GraphPad Prism software version 4 for Windows (GraphPad).

### Treatment with Epigenetic Chemical Inhibitors

To analyze the DNA demethylation effects induced by 5-aza-2′-deoxycytidine (5AzaCdR; Calbiochem, Merck, Darmstadt, Germany) on a genome-wide scale and in specific genes, mouse cell lines (melan-a, 4C, 4C11− and 4C11+) in log phase were incubated in medium supplemented with 10 µM of the chemical compound for 48 hours. To analyze the histone deacetylase effects induced by Trichostatin A (TSA; Calbiochem) on gene-specific expression, mouse cell lines were incubated in medium supplemented with 40 nM of the chemical compound for 16 hours. Finally, to analyze the synergistic effects between 5AzaCdR and TSA on gene-specific expression, mouse and human cell lines were incubated in medium supplemented with the both compounds, at the concentrations and time indicated. In all of them, inhibitor-treated cell lines were compared against their control cell counterparts which were cultured in a paired way but in inhibitor(s)-free culture medium. These treatment conditions do not significantly induce cell toxicity since they were not able to impair cell proliferation, as previously determined by us after performing methyl thiazol tetrazolium (MTT) assays [11]. In addition, Trypan Blue stain 0.4% was used to monitor cell viability in a Countess Automated Cell Counter (Invitrogen, Carlsbad, CA).

### Digestion of Genomic DNA with MspI and HpaII

To evaluate the relative amount of global DNA methylation, 2 µg of genomic DNA extracted from melan-a, 4C, 4C11− and 4C11+ monolayers, as well as their 5AzaCdR-treated counterpart cells, were digested or not with 2 µL of enzymes MspI or HpaII in separeted reactions (Fast Digest, Fermentas, MD). The DNA was digested for 16 hours at 37°C. Afterwards, 1 µL of each enzyme was added, and the reactions were maintained for 1 hour at 37°C. Then, samples were resolved onto 0.8% agarose gel electrophoresis. The intensity of the bands corresponding to intact genomic DNA, genomic DNA digested with MspI, and genomic DNA digested with HpaII in each sample was determined using the ImageJ software. Percentage of methylation was measured as follows: relative global DNA methylation content =  (HpaII-MspI) ×100/ genomic DNA. Percentage of demethylation was calculated as follows: 100-percentage of methylation.

### RNA Extraction and Microarray-Based Gene Expression Studies

Mouse cell lines (melan-a, 4C, 4C11− and 4C11+) were grown and treated as previously described. For DNA demethylation and high-throughput microarray-based gene expression studies, cells were harvested by trypsinization at 48 hours. Total cell RNA which was extracted from 5AzaCdR-treated and control cell monolayers using TRIzol reagent (Invitrogen), was purified using RNEasy MinElute Cleanup spin columns (QIAGEN, Dusseldorf, Germany), according to manufacturer's instructions. Only RNA samples with an absorbance A_260_/A_280_ nm ratio greater than 2.0 were used for microarray hybridization. Briefly, 2 µg from total RNA were amplified by an *in vitro* transcription reaction and biotin-labeled. 3′-IVT Expression Arrays (GeneChip Mouse Genome 430_2.0, format 49; Affymetrix Inc., Santa Clara, CA) which were hybridized under standard conditions for 16 hours at 45°C and 60 rpm, were washed, stained with streptavidin-phycoerythrin and scanned using an Affymetrix GeneChip Scanner 3000 7G. Gene expression experiments using Mouse 430_2.0 Arrays were done following the Affymetrix Expression Analysis Technical Manual. Hybridization reactions and scanning were carried out at Associação Fundo de Incentivo à Psicofarmacologia (AFIP, Brazil).

### Microarray and Pathway Analyses

In the chosen arrays, there are 45,101 probe sets which are designed to investigate approximately 34,000 mouse transcripts. We used GCOS software version 1.4 (Affymetrix) to extract expression values (raw array data) generated from Mouse 430_2.0 Array experiments, according to standard procedures by Affymetrix Systems. To identify differentially expressed genes in the transitions from melan-a to 4C, 4C to 4C11−, and 4C11− to 4C11+ cell lines, class-comparison experiments were performed, and transcripts were selected as statistically significant by the two-class unpaired SAM analysis with 700 random permutations (FDR<0.05). The experiments were carried out in three biological replicates. To estimate differences on gene responsiveness upon 5AzaCdR treatment in each cell line, 5AzaCdR-treated cells were compared against their previously untreated counterpart cells. Transcripts were selected as statistically significant by the two-class paired SAM analysis with 500 random permutations (FDR<0.05). The experiments were carried out in two biological replicates.

The raw array data (.CEL files) were first quantile normalized with background correction using RMAExpress version 1.0.5 [54]. Next, the normalized expression values (log_2_ scale) were filtered due to strict quality criteria. Filtering and statistics were carried out using MultiExperiment Viewer (MeV) version 4.6 from The Institute of Genomic Research (TIGR; Rockville, MD) [55]. This filtering reduces the number of probes to 36,080 but increases the consistency of the measurements as shown by the removal of genes that did not vary much in expression over the conditions of the experiment. This variance filter was performed after quality control filters, such as lower and percentage cutoffs, were imposed. This methodology insures that the genes that were checked for variance also contained a level of quality data. Afterwards, genes showing at least a two-fold enrichment and a median false discovery rate (FDR) and a *Q*-value threshold of 5% after SAM statistical analysis [56], were chosen. For all comparisons, we removed probes that corresponded to hypothetic proteins, riken cDNAs, cDNA sequences and transcribed loci because all of them are *in silico* predicted or not well characterized just yet. This approach yielded a more accurate identification of differences on global gene expression profiles, making the final data sets more reliable for future validation. The differentially expressed genes identified here were submitted to an unsupervised hierarchical clustering analysis (HCL) [57] using the average linkage algorithm and the Pearson correlation metric distance to arrange genes and samples according to similarity in patterns of gene expression.

Pathway analysis was generated by using the PANTHER software. The binomial test was applied to determine whether there was a statistical representation of genes (*P*<0.05) [58].

### Accession Numbers

The raw microarray data are accessible from the ArrayExpress (E-MEXP-2512 and E-MEXP-2517).

### Two-Step Real-Time RT-PCR

We next carried out studies to better evaluate the potential impact, directly or indirectly, of DNA methylation and/or histone deacetylation on the expression of specific genes that were selected based on microarray results. To do this, mouse cell lines (melan-a, 4C, 4C11− and 4C11+) were grown and treated as described above. Total cell RNA was extracted from 5AzaCdR and/or TSA-treated and untreated (control) cell monolayers using TRIzol reagent (Invitrogen). One microgram of RNA samples was reverse amplified for cDNA synthesis with integrated removal of genomic DNA contamination using QuantiTect Reverse Transcription Kit (QIAGEN), according to manufacturer's instructions.

The quality of the cDNA templates was checked by amplifying the endogenous reference gene *Actb*. With the aim of determining amplification efficiencies, we compared dilution series of reference and target genes from a reference cDNA template. Serial dilutions were amplified in two-step RT-qPCR and the *C_q_* values obtained were used to construct standard curves for *Actb, Fblim1*, *Hspb1, Serpine1* and *Xist* genes. The amplification efficiency of each gene was determined as follows: *E = 10^(-1/S)^ – 1*, in which *E* corresponds to the efficiency and *S* to the slope of the standard curve. Next, equal amounts of each cDNA synthesized (200 ng/reaction) were quantified by real-time PCR in a Corbett Rotor-Gene 6000 Detection System version 1.7 using a SYBR green PCR master mix (QIAGEN) and a final concentration of specific forward and reverse primers at 400 nM. PCR cycling conditions were set to 5 minutes at 95°C followed by 40 cycles of 5 seconds at 95°C and 10 seconds at 60°C. A signal for non-template control (NTC) was evaluated as a control reaction since it enables detection of contamination. Transcripts presenting *C_q_* values ≥ 35 were considered non-expressed. Because of the target(s) and the endogenous reference gene were amplified with comparable efficiencies (90%≤*E*≤110%), the relative quantification (RQ) of the amplicons was calculated according to *2^-ΔΔCq^* method. Mouse *Actb* was used as a single reference gene for normalizing cellular mRNA data because of its more stable expression among the previously untreated and treated cell lines than *Gapdh* gene (data not shown).

To analyze the differences in the relative expression levels of genes among the sample groups representing distinct phases of melanocyte malignant transformation (melan-a, 4C, 4C11− and 4C11+), melan-a melanocyte lineage was taken as a reference sample and its average value was set arbitrarily to 1. For experiments involving treatment groups, untreated cell lines were taken as reference samples, and their average values were set arbitrarily to 1.

### Human Studies

Human studies were performed with the aim of discussing the potential application of our mouse findings in a way more closely related to the pathogenesis of human melanomas. To do this, total mRNA was isolated from FM305 human primary melanocytes and metastatic melanoma cell lines (Mel-2, Mel-3, Mel-11, Mel-14 and Mel-33), and analyzed by two-step RT-qPCR, as described for mouse experiments. In this case, we selected a panel comprised by the genes *CTCF*, *FBLIM1*, *HSPB1*, *NDRG2*, *NSD1*, *SERPINE1*, *SRC* and *VDR* to address their potential use as biomarkers in identifying aggressive melanoma cells. To evaluate the differences in the relative expression levels of genes between the two sample groups (primary melanocytes and metastatic melanomas), the human primary melanocyte was used as a reference sample, and its average value was set arbitrarily to 1. For experiments involving treatment groups, untreated cell lines were taken as reference samples, and their average values were set arbitrarily to 1. Unsupervised hierarchical clustering (Pearson correlation metric distance; average linkage algorithm) and relevance network prediction [59] of the genes validated in human primary melanocytes and five early-passage metastatic melanoma cell lines studied by RT-qPCR was performed using MeV software.

Additionally, a conventional RT-PCR approach was used to assess the expression of *XIST* in two female human metastatic melanoma cell lines (Mel-11 and Mel-33) using a recombinant Platinum Taq DNA Polymerase (Invitrogen) and a final concentration of specific forward and reverse primers at 10 µM. PCR cycling conditions were set to 2 minutes at 94°C followed by 25 cycles of 30 seconds at 94°C, 30 seconds at 60°C, 30 seconds at 72°C and 7 minutes at 72°C.

### Primer Design for RT-qPCR

To avoid amplification of genomic DNA, we designed mouse (Integrated DNA Technologies Inc., Coralville, IA) and human (Sigma-Aldrich) primer pairs flanking introns using IDT PrimerQuest and Primer3 web interface. All primer sequences are listed (**Table S5**).

### Statistical Analysis

Statistical analysis of RT-qPCR results was performed by Student's *t* test or One Way ANOVA test followed by the post-hoc Tukey. All statistics were carried out using GraphPad Prism software version 5 for Windows (GraphPad). Error bars represent SEM. The significance level was established at *P*<0.05. *P<0.05, **P<0.01, ***P<0.001.

### International Guidelines

The Minimum Information About a Microarray Experiment (MIAME) as well as The Minimum Information for Publication of Quantitative Real-Time PCR Experiments (MIQE) guidelines were used for the better experimental design and interpretation of microarray and qPCR results, respectively [60], [61].

### URLs

ArrayExpress can be found at http://www.ebi.ac.uk/arrayexpress/. IDT PrimerQuest can be found at http://www.idtdna.com/home/home.aspx. MeV can be found at http://www.tm4.org/mev/. PANTHER software can be found at http://www.pantherdb.org/tools. Primer3 web interface can be found at http://frodo.wi.mit.edu/primer3/input.htm. RMAExpress can be found at http://rmaexpress.bmbolstad.com.

## Supporting Information

Figure S1 **Karyotype Evolutionary Description.** Overall chromosomal rearrangements were shown in the representative images of the metaphases derived from karyotypes of (A) 4C11− non-metastatic, and (B) 4C11+ metastatic melanoma cell lines. *Left panel*: the raw image of a metaphase. *Central panel*: the classified image of the metaphase. *Right panel*: the inverted DAPI-banded image of the metaphase. *Larger panel*: the karyotype table of the metaphase.
(TIF)

Figure S2 **Hierarchical Clustering of Samples and Differentially Expressed Genes Upon 5-aza-2′-deoxycytidine Exposure Across the Melanoma Progression Spectrum.**
(TIF)

Figure S3 **Melanoma Stem Cell Markers Dynamically Regulated during melan-a Malignant Transformation.** Green: Low expression; Red: High expression.
(PPT)

Table S1 **Transcripts Up-Regulated in melan-a Melanocytes Following 5-aza-2′-deoxycytidine Treatment Identified by Genome-Wide Screening.**
(DOC)

Table S2 **Transcripts Up-Regulated in 4C Pre-Malignant Melanocytes Following 5-aza-2′-deoxycytidine Treatment Identified by Genome-Wide Screening.**
(DOC)

Table S3 **Transcripts Up-Regulated in 4C11− Non-Metastatic Melanoma Cells Following 5-aza-2′-deoxycytidine Treatment Identified by Genome-Wide Screening.**
(DOC)

Table S4 **Transcripts Up-Regulated in 4C11+ Metastatic Melanoma Cells Following 5-aza-2′-deoxycytidine Treatment Identified by Genome-Wide Screening.**
(DOC)

Table S5 **Primers Used for Amplification by RT-qPCR.**
(DOC)
