# Supplementary figures and images for: Mining Gene Expression Signature for the Detection of Pre-Malignant Melanocytes and Early Melanomas with Risk for Metastasis
Source: PLoS One. 2012 Sep 11;7(9):e44800. doi: 10.1371/journal.pone.0044800 (PMC3439384; doi:10.1371/journal.pone.0044800)

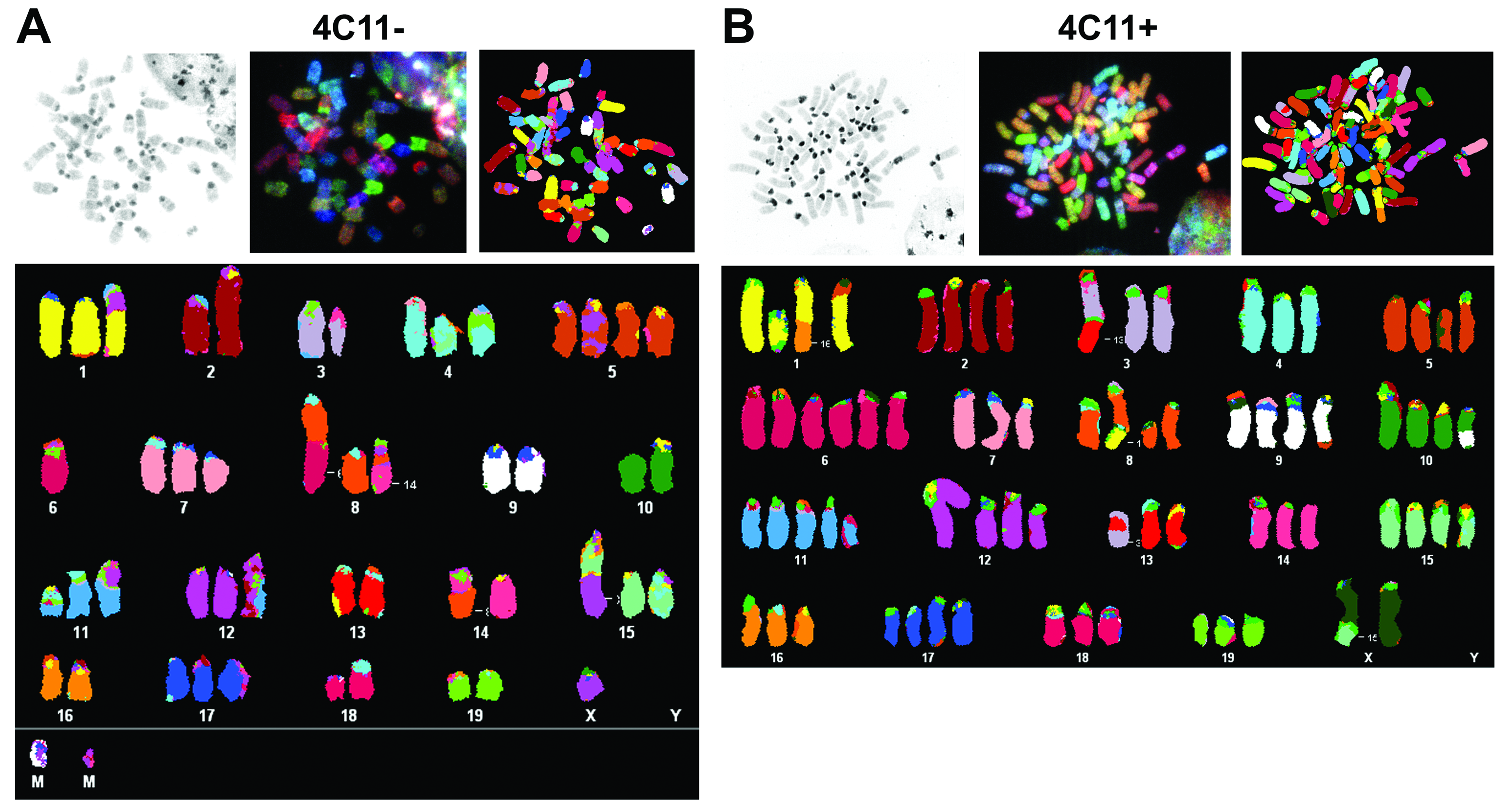

Supplement: Figure S1 — Karyotype Evolutionary Description. Overall chromosomal rearrangements were shown in the representative images of the metaphases derived from karyotypes of (A) 4C11− non-metastatic, and (B) 4C11+ metastatic melanoma cell lines. Left panel: the raw image of a metaphase. Central panel: the classified image of the metaphase. Right panel: the inverted DAPI-banded image of the metaphase. Larger panel: the karyotype table of the metaphase. (TIF) [file pone.0044800.s001.tif]

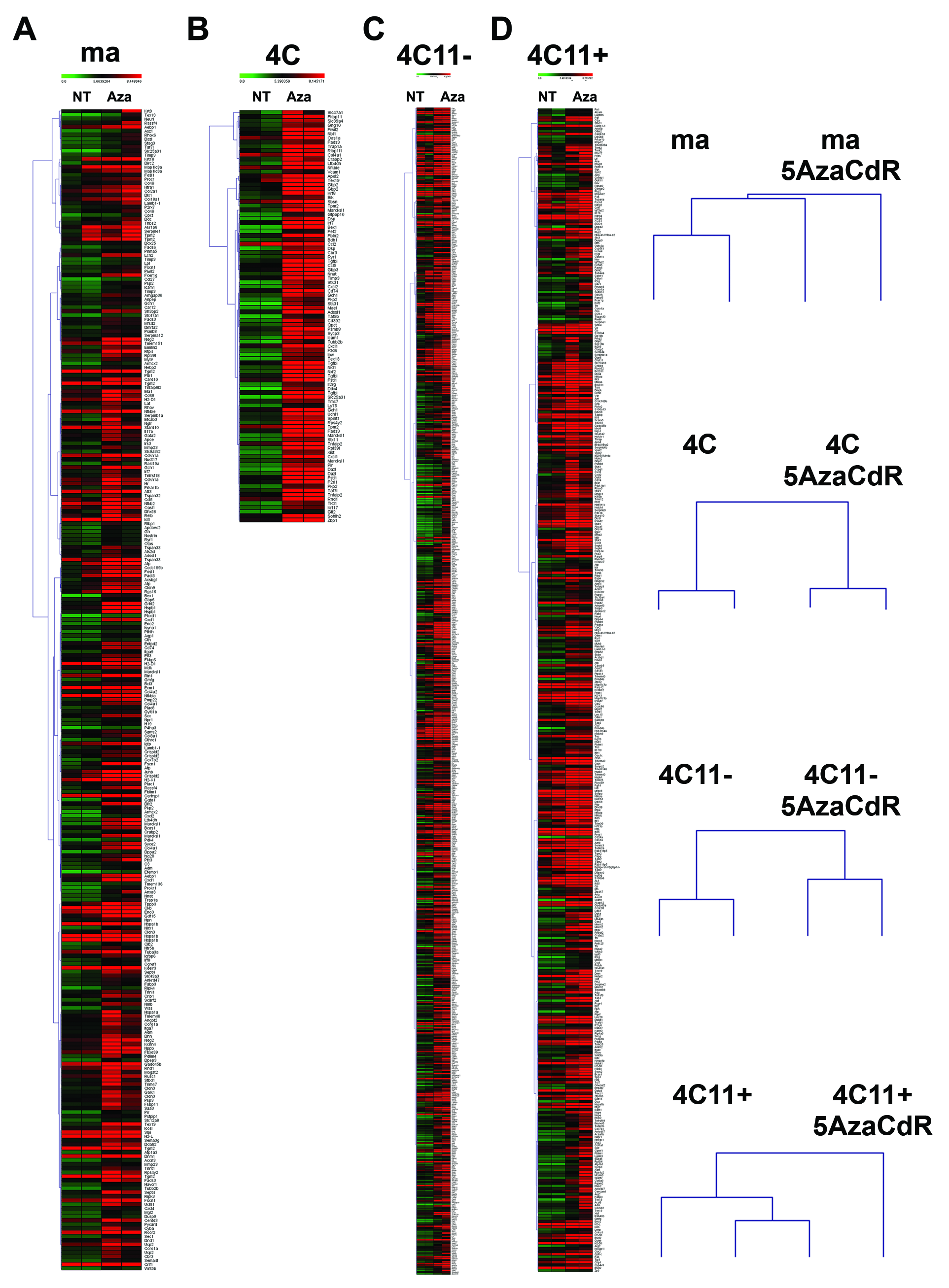

Supplement: Figure S2 — Hierarchical Clustering of Samples and Differentially Expressed Genes Upon 5-aza-2′-deoxycytidine Exposure Across the Melanoma Progression Spectrum. (TIF) [file pone.0044800.s002.tif]
