# Supplementary material for: Mining Gene Expression Signature for the Detection of Pre-Malignant Melanocytes and Early Melanomas with Risk for Metastasis
Source: PLoS One. 2012 Sep 11;7(9):e44800. doi: 10.1371/journal.pone.0044800 (PMC3439384; doi:10.1371/journal.pone.0044800)
Supplement: Figure S3 — Melanoma Stem Cell Markers Dynamically Regulated during melan-a Malignant Transformation. Green: Low expression; Red: High expression. (PPT) [file pone.0044800.s003.ppt]

## Slide 1
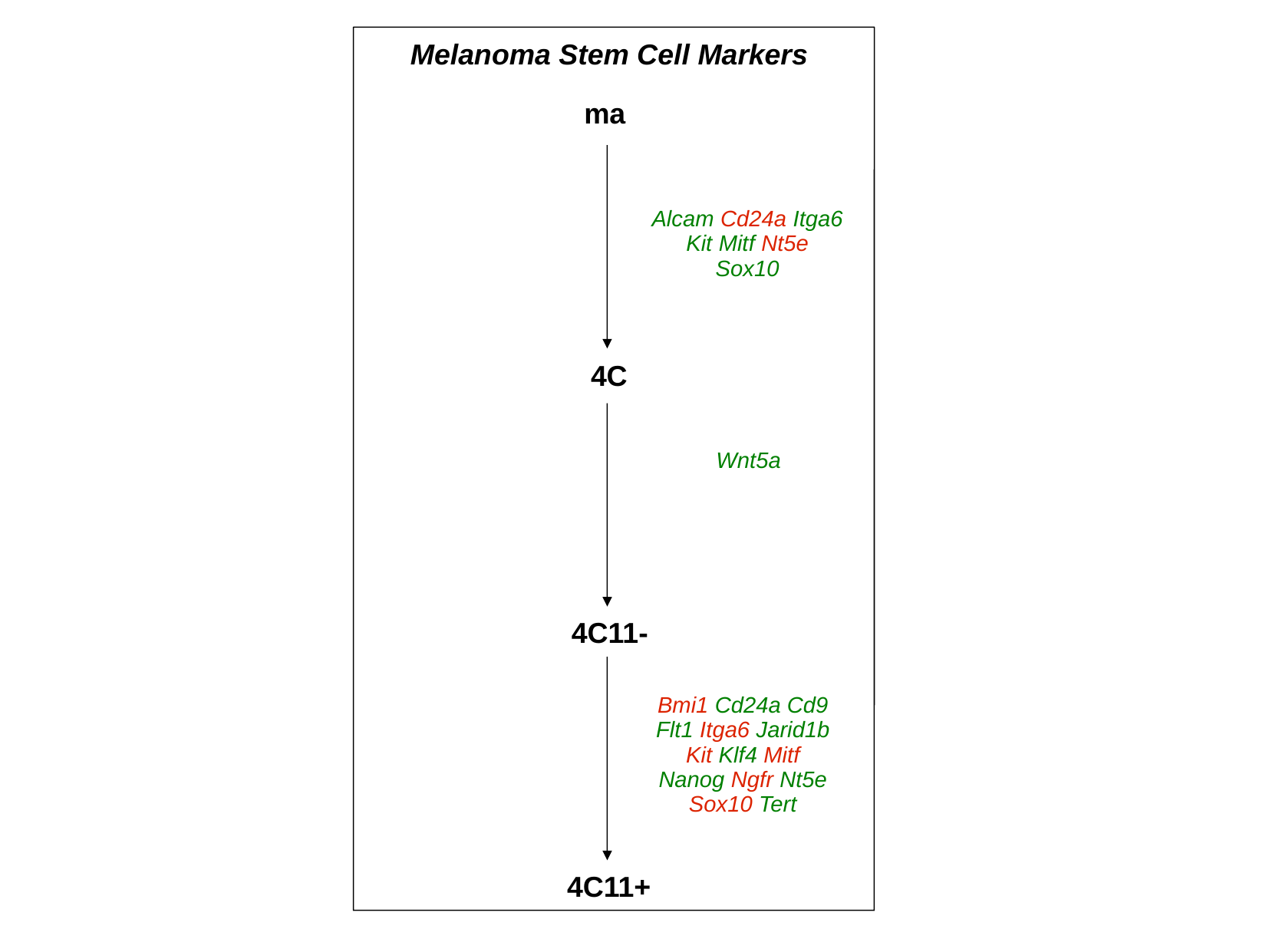

Melanoma Stem Cell Markers
ma
Alcam Cd24a Itga6
Kit Mitf Nt5e
Sox10
4C
Wnt5a
4C11-
Bmi1 Cd24a Cd9
Flt1 Itga6 Jarid1b
Kit Klf4 Mitf
Nanog Ngfr Nt5e
Sox10 Tert
4C11+
