# Supplementary material for: Mining Gene Expression Signature for the Detection of Pre-Malignant Melanocytes and Early Melanomas with Risk for Metastasis
Source: PLoS One. 2012 Sep 11;7(9):e44800. doi: 10.1371/journal.pone.0044800 (PMC3439384; doi:10.1371/journal.pone.0044800)
Supplement: Table S1 — Transcripts Up-Regulated in melan-a Melanocytes Following 5-aza-2′-deoxycytidine Treatment Identified by Genome-Wide Screening. (DOC) [file pone.0044800.s004.doc]

**Table S1. Transcripts Up-Regulated in melan-a Melanocytes Following 5-aza-2'-deoxycytidine Treatment Identified by Genome-Wide Screening.**

| **Probe Set_ID** | **Gene** | **Fold-Enrichment** | ***Q*-Value (%)** |
| --- | --- | --- | --- |
| 1439983_a_at | *Accn3* | 2.893648 | 2.9567993 |
| 1422428_at | *Acsbg1* | 3.0759332 | 3.3774319 |
| 1447839_x_at | *Adm* | 2.5361555 | 3.929698 |
| 1416077_at | *Adm* | 2.5685446 | 3.9862034 |
| 1449383_at | *Adssl1* | 2.2026439 | 3.3936598 |
| 1450637_a_at | *Aebp1* | 2.7564473 | 2.820427 |
| 1422514_at | *Aebp1* | 3.006666 | 4.7834444 |
| 1416645_a_at | *Afp* | 4.4416065 | 4.484479 |
| 1436881_x_at | *Afp* | 2.3350327 | 4.575999 |
| 1416646_at | *Afp* | 3.1939049 | 4.5494714 |
| 1448894_at | *Akr1b8* | 2.3407686 | 2.6853168 |
| 1433769_at | *Als2cl* | 2.543777 | 3.297411 |
| 1448831_at | *Angpt2* | 2.5655072 | 4.1432686 |
| 1448839_at | *Ankrd47* | 2.6988058 | 4.526919 |
| 1421424_a_at | *Anpep* | 2.1095448 | 3.3774319 |
| 1460330_at | *Anxa3* | 10.99696 | 2.3602521 |
| 1417889_at | *Apobec2* | 3.6581442 | 2.2997327 |
| 1432466_a_at | *Apoe* | 2.8934515 | 2.5921843 |
| 1416203_at | *Aqp1* | 2.638828 | 3.3218362 |
| 1436171_at | *Arhgap30* | 3.3696046 | 4.322389 |
| 1415780_a_at | *Armcx2* | 3.2578669 | 3.7897005 |
| 1456739_x_at | *Armcx2* | 9.037577 | 0.0 |
| 1424939_at | *Asz1* | 2.5878086 | 3.5732899 |
| 1449363_at | *Atf3* | 3.3125243 | 4.335448 |
| 1427481_a_at | *Atp1a3* | 2.749807 | 3.4495993 |
| 1428792_at | *Bcas1* | 2.8004506 | 1.2812797 |
| 1418133_at | *Bcl3* | 2.3019848 | 2.5921843 |
| 1448595_a_at | *Bex1* | 16.697065 | 0.0 |
| 1423954_at | *C3* | 2.3688266 | 4.191102 |
| 1428485_at | *Car12* | 2.1211863 | 2.9119992 |
| 1449491_at | *Card10* | 3.702219 | 2.135466 |
| 1415975_at | *Carhsp1* | 2.1008177 | 2.8382778 |
| 1427912_at | *Cbr3* | 5.300837 | 1.3798397 |
| 1418778_at | *Ccdc109b* | 3.2190356 | 4.250691 |
| 1434962_x_at | *Ccl27* | 2.3266687 | 4.3669086 |
| 1418126_at | *Ccl5* | 3.6058893 | 4.484479 |
| 1460415_a_at | *Cd40* | 2.0332718 | 4.526919 |
| 1439221_s_at | *Cd40* | 2.0509813 | 3.540378 |
| 1449164_at | *Cd68* | 3.0699453 | 4.335448 |
| 1425519_a_at | *Cd74* | 3.3824682 | 1.6609181 |
| 1421679_a_at | *Cdkn1a* | 3.8047066 | 4.0336585 |
| 1424638_at | *Cdkn1a* | 4.1118865 | 2.8382778 |
| 1419833_s_at | *Centd3* | 2.8438501 | 4.318387 |
| 1424529_s_at | *Cgref1* | 2.3873365 | 4.1432686 |
| 1422852_at | *Cib2* | 2.8373888 | 3.8600578 |
| 1455106_a_at | *Ckb* | 2.9802878 | 4.335448 |
| 1460569_x_at | *Cldn3* | 3.7537167 | 3.5095923 |
| 1426332_a_at | *Cldn3* | 3.350466 | 3.8622308 |
| 1451701_x_at | *Cldn3* | 4.1746325 | 3.0715609 |
| 1439427_at | *Cldn9* | 3.6342723 | 3.5172384 |
| 1426955_at | *Col18a1* | 2.1141372 | 3.605611 |
| 1450567_a_at | *Col2a1* | 2.1973429 | 3.7897005 |
| 1426348_at | *Col4a1* | 2.7216234 | 3.5591102 |
| 1452035_at | *Col4a1* | 6.2398715 | 0.0 |
| 1424051_at | *Col4a2* | 5.505225 | 2.6379287 |
| 1418440_at | *Col8a1* | 3.007401 | 1.908289 |
| 1416246_a_at | *Coro1a* | 3.9721942 | 3.0232441 |
| 1455269_a_at | *Coro1a* | 4.123018 | 3.4232664 |
| 1429687_at | *Cox7b2* | 2.7617135 | 3.816578 |
| 1451191_at | *Crabp2* | 2.5771043 | 3.8995469 |
| 1416326_at | *Crip1* | 3.2501938 | 3.3218362 |
| 1460458_at | *Crispld2* | 2.1148508 | 4.390399 |
| 1434758_at | *Crispld2* | 3.2292535 | 3.8995469 |
| 1437056_x_at | *Crispld2* | 3.7528026 | 1.9497734 |
| 1418476_at | *Crlf1* | 2.944061 | 3.3218362 |
| 1426243_at | *Cth* | 2.4729714 | 2.9119992 |
| 1452968_at | *Cthrc1* | 2.478155 | 4.220686 |
| 1457644_s_at | *Cxcl1* | 5.4976115 | 0.0 |
| 1419209_at | *Cxcl1* | 15.851117 | 0.0 |
| 1449984_at | *Cxcl2* | 3.670615 | 1.7247996 |
| 1448995_at | *Cxcl4* | 2.849596 | 3.7719917 |
| 1454268_a_at | *Cyba* | 6.910153 | 1.5201623 |
| 1449502_at | *Dazl* | 5.1593995 | 2.9119992 |
| 1416457_at | *Ddah2* | 2.7807345 | 3.5697346 |
| 1426215_at | *Ddc* | 2.2203112 | 4.46852 |
| 1418263_at | *Ddx25* | 2.0193276 | 4.318387 |
| 1434959_at | *Dhh* | 2.8662026 | 4.484479 |
| 1451426_at | *Dhx58* | 4.341473 | 1.7586192 |
| 1454654_at | *Dirc2* | 2.3349326 | 4.10627 |
| 1449470_at | *Dlx1* | 2.2602878 | 3.6758025 |
| 1448877_at | *Dlx2* | 4.830429 | 0.0 |
| 1441107_at | *Dmrta2* | 3.162505 | 2.1875508 |
| 1455367_at | *Dnd1* | 2.5776875 | 3.5172384 |
| 1460365_a_at | *Dnm1* | 2.1610165 | 4.370026 |
| 1429035_at | *Dpep3* | 3.5835154 | 4.356351 |
| 1453223_s_at | *Dppa2* | 3.2106535 | 4.3751016 |
| 1433845_x_at | *Dusp9* | 2.5372689 | 4.442827 |
| 1448613_at | *Ecm1* | 2.563604 | 3.4830904 |
| 1453766_a_at | *Efcab3* | 11.352463 | 3.7764034 |
| 1427183_at | *Efemp1* | 2.904781 | 1.2998489 |
| 1423693_at | *Ela1* | 4.6643634 | 3.587583 |
| 1416916_at | *Elf3* | 2.0961435 | 4.6743755 |
| 1435264_at | *Emilin2* | 5.8261666 | 0.0 |
| 1418829_a_at | *Eno2* | 2.0277617 | 3.25299 |
| 1417951_at | *Eno3* | 3.2088406 | 4.526919 |
| 1418259_a_at | *Entpd2* | 2.5692663 | 3.8995469 |
| 1416023_at | *Fabp3* | 2.678906 | 4.335448 |
| 1435910_at | *Fads3* | 2.2733555 | 3.706181 |
| 1418773_at | *Fads3* | 4.698631 | 1.8303995 |
| 1443904_at | *Fads6* | 2.774599 | 3.86593 |
| 1418569_at | *Fblim1* | 2.345645 | 4.076799 |
| 1443698_at | *Fbxo39* | 3.7493374 | 3.5732899 |
| 1418340_at | *Fcer1g* | 4.8614326 | 1.4236441 |
| 1417267_s_at | *Fkbp11* | 6.2897716 | 4.076799 |
| 1425101_a_at | *Fkbp6* | 3.3698528 | 3.7370658 |
| 1417488_at | *Fosl1* | 2.2597623 | 4.294994 |
| 1417487_at | *Fosl1* | 3.6870172 | 4.526919 |
| 1448378_at | *Fscn1* | 2.046695 | 4.1432686 |
| 1416515_at | *Fscn1* | 3.1941361 | 3.5591102 |
| 1416514_a_at | *Fscn1* | 28.795673 | 0.0 |
| 1450971_at | *Gadd45b* | 3.4097924 | 3.755475 |
| 1417177_at | *Galk1* | 4.8068104 | 3.8995469 |
| 1428816_a_at | *Gata2* | 2.1430247 | 4.1432686 |
| 1425156_at | *Gbp6* | 2.0325587 | 3.880799 |
| 1429692_s_at | *Gch1* | 2.2692556 | 3.7897005 |
| 1420499_at | *Gch1* | 5.3170676 | 4.1716084 |
| 1418949_at | *Gdf15* | 8.000587 | 3.4232664 |
| 1418483_a_at | *Ggta1* | 6.2990294 | 0.0 |
| 1460613_x_at | *Gh* | 2.3890364 | 2.874666 |
| 1419194_s_at | *Gmfg* | 3.8393087 | 1.6609181 |
| 1427046_at | *Grhl2* | 8.327005 | 0.0 |
| 1434007_at | *Gyltl1b* | 2.1035848 | 4.484479 |
| 1448194_a_at | *H19* | 2.5282161 | 3.6525168 |
| 1451683_x_at | *H2-D1* | 2.8314104 | 3.8023014 |
| 1425545_x_at | *H2-D1* | 2.3225133 | 3.6525168 |
| 1425336_x_at | *H2-K1* | 3.4132366 | 1.546372 |
| 1451931_x_at | *H2-L* | 2.392342 | 3.794559 |
| 1434716_at | *Havcr1* | 2.7813861 | 3.706181 |
| 1449271_a_at | *Hebp2* | 2.779837 | 4.191102 |
| 1420712_a_at | *Hpn* | 2.4433243 | 4.3193283 |
| 1435950_at | *Hr* | 4.116203 | 4.1432686 |
| 1452388_at | *Hspa1a* | 8.595713 | 4.1432686 |
| 1452318_a_at | *Hspa1b* | 2.5893981 | 3.755475 |
| 1427127_x_at | *Hspa1b* | 3.1445036 | 4.318387 |
| 1427126_at | *Hspa1b* | 3.199185 | 3.645918 |
| 1422943_a_at | *Hspb1* | 6.4447713 | 0.0 |
| 1425964_x_at | *Hspb1* | 10.049072 | 0.0 |
| 1422196_at | *Htr5b* | 3.7120917 | 4.526919 |
| 1416749_at | *Htra1* | 2.6067672 | 3.2733424 |
| 1424067_at | *Icam1* | 3.2468092 | 3.6909292 |
| 1419212_at | *Icosl* | 2.1115708 | 4.335448 |
| 1416630_at | *Id3* | 2.7961948 | 3.5697346 |
| 1417933_at | *Igfbp6* | 2.3441684 | 4.370026 |
| 1417141_at | *Igtp* | 2.05508 | 3.880799 |
| 1431693_a_at | *Il17b* | 3.504053 | 3.0715609 |
| 1417244_a_at | *Irf7* | 3.6531198 | 2.9698536 |
| 1416714_at | *Irf8* | 2.2372441 | 4.370026 |
| 1437672_at | *Irs3* | 2.268543 | 4.5110145 |
| 1419569_a_at | *Isg20* | 2.3491836 | 2.874666 |
| 1415977_at | *Isyna1* | 2.3268216 | 3.5520625 |
| 1418393_a_at | *Itga7* | 2.3068063 | 4.250691 |
| 1450029_s_at | *Itga9* | 3.1125166 | 1.3798397 |
| 1415899_at | *Junb* | 3.2634304 | 3.2262437 |
| 1435945_a_at | *Kcnn4* | 3.3150187 | 4.4977074 |
| 1418538_at | *Kdelr3* | 2.5054529 | 4.4194865 |
| 1448169_at | *Krt18* | 3.5450437 | 2.5921843 |
| 1423691_x_at | *Krt8* | 4.4794283 | 2.7681968 |
| 1424113_at | *Lamb1-1* | 2.388159 | 3.8576162 |
| 1424114_s_at | *Lamb1-1* | 2.8760734 | 1.9931017 |
| 1460651_at | *Lat* | 2.5749004 | 3.755475 |
| 1427747_a_at | *Lcn2* | 3.480229 | 2.8027992 |
| 1415904_at | *Lpl* | 2.0823493 | 4.370026 |
| 1417777_at | *Ltb4dh* | 14.901344 | 0.0 |
| 1451290_at | *Map1lc3a* | 2.3787103 | 4.1432686 |
| 1447883_x_at | *Map1lc3a* | 2.7386587 | 4.46852 |
| 1435415_x_at | *Marcksl1* | 2.8608959 | 3.4232664 |
| 1415922_s_at | *Marcksl1* | 5.3658967 | 0.0 |
| 1437226_x_at | *Marcksl1* | 30.146202 | 0.0 |
| 1416006_at | *Mdk* | 2.2754076 | 3.4495993 |
| 1428223_at | *Mfsd2* | 2.033915 | 3.304353 |
| 1438467_at | *Mgl2* | 2.962701 | 3.6525168 |
| 1417281_a_at | *Mmp23* | 2.0657978 | 4.10627 |
| 1417282_at | *Mmp23* | 2.085062 | 4.1432686 |
| 1455099_at | *Mogat2* | 3.712499 | 3.8600578 |
| 1452670_at | *Myl9* | 5.1993566 | 2.5625594 |
| 1433720_s_at | *Ndg2* | 6.7757473 | 3.706181 |
| 1436990_s_at | *Ndg2* | 9.46767 | 0.0 |
| 1456854_at | *Neurl* | 2.5067055 | 3.830962 |
| 1425902_a_at | *Nfkb2* | 2.0591857 | 3.929698 |
| 1449731_s_at | *Nfkbia* | 2.0748308 | 3.587583 |
| 1458299_s_at | *Nfkbie* | 2.415242 | 4.1432686 |
| 1454903_at | *Ngfr* | 2.5036814 | 4.087729 |
| 1433661_at | *Nlrx1* | 3.287965 | 3.9568932 |
| 1419405_at | *Nmb* | 2.8290749 | 3.7027807 |
| 1423506_a_at | *Nnat* | 2.5800912 | 4.452674 |
| 1441075_at | *Nostrin* | 2.093277 | 4.566015 |
| 1450791_at | *Nppb* | 11.818547 | 3.9685655 |
| 1449160_at | *Npr1* | 2.6969523 | 3.4495993 |
| 1452995_at | *Nudt17* | 2.6318436 | 4.442827 |
| 1424339_at | *Oasl1* | 4.0446496 | 4.9144974 |
| 1426193_at | *Otos* | 2.1058033 | 3.587583 |
| 1419853_a_at | *P2rx7* | 2.235625 | 3.706181 |
| 1446951_at | *P4ha3* | 2.053207 | 3.8600578 |
| 1419767_at | *Padi3* | 4.217595 | 4.705027 |
| 1417273_at | *Pdk4* | 2.1531327 | 3.3936598 |
| 1417928_at | *Pdlim4* | 7.43276 | 3.0300534 |
| 1429001_at | *Pir* | 4.4393177 | 1.2998489 |
| 1449170_at | *Piwil2* | 4.6552143 | 1.5735013 |
| 1449799_s_at | *Pkp2* | 4.6565437 | 4.9144974 |
| 1429183_at | *Pkp2* | 3.1520312 | 1.9931017 |
| 1418831_at | *Pkp3* | 2.3407416 | 3.830962 |
| 1417553_at | *Plac1* | 2.278039 | 3.1869392 |
| 1451335_at | *Plac8* | 2.3004577 | 3.880799 |
| 1437893_at | *Plb1* | 2.3560958 | 2.717866 |
| 1437842_at | *Plcxd1* | 2.6479874 | 4.7205043 |
| 1417133_at | *Pmp22* | 7.739436 | 0.0 |
| 1439207_at | *Pnma5* | 2.138245 | 4.6591988 |
| 1434325_x_at | *Prkar1b* | 3.5113661 | 3.929698 |
| 1420664_s_at | *Procr* | 2.4720562 | 4.390399 |
| 1456543_at | *Prokr1* | 6.2471943 | 1.8303995 |
| 1422962_a_at | *Psmb8* | 2.3777163 | 3.645918 |
| 1424560_at | *Pstpip1* | 2.1095262 | 4.270932 |
| 1427527_a_at | *Pthlh* | 2.3279917 | 3.5520625 |
| 1418666_at | *Ptx3* | 7.3468833 | 0.0 |
| 1417346_at | *Pycard* | 4.3144712 | 3.4232664 |
| 1426622_a_at | *Qpct* | 2.234081 | 3.1869392 |
| 1427975_at | *Rasl10a* | 2.3518965 | 4.526919 |
| 1439622_at | *Rassf4* | 4.7520804 | 3.1869392 |
| 1444009_at | *Rassf4* | 5.6554494 | 2.4240427 |
| 1417302_at | *Rcor2* | 2.135706 | 3.755475 |
| 1417856_at | *Relb* | 3.6473162 | 1.4466062 |
| 1426037_a_at | *Rgs16* | 3.6885252 | 4.370026 |
| 1424976_at | *Rhov* | 3.3396244 | 3.8023014 |
| 1419018_at | *Rhox6* | 3.42681 | 3.880799 |
| 1424507_at | *Rin1* | 2.1669753 | 4.575999 |
| 1448449_at | *Ripk3* | 3.4736211 | 4.076799 |
| 1418488_s_at | *Ripk4* | 2.7981277 | 4.270932 |
| 1418310_a_at | *Rlbp1* | 3.0762644 | 2.9731352 |
| 1455197_at | *Rnd1* | 4.062662 | 4.5110145 |
| 1423327_at | *Rpl39l* | 9.478391 | 0.0 |
| 1452730_at | *Rps4y2* | 16.4636 | 0.0 |
| 1418580_at | *Rtp4* | 4.602354 | 0.0 |
| 1434743_x_at | *Rusc1* | 3.938555 | 4.270932 |
| 1427306_at | *Ryr1* | 2.067748 | 4.442827 |
| 1450826_a_at | *Saa3* | 2.4267468 | 3.9280107 |
| 1434740_at | *Scarf2* | 2.9282162 | 3.0403247 |
| 1428983_at | *Scx* | 2.7919865 | 3.4232664 |
| 1455784_at | *Sec1* | 3.3074868 | 2.5921843 |
| 1435361_at | *Sema3g* | 2.9500234 | 2.6853168 |
| 1439768_x_at | *Sema4f* | 4.4437013 | 1.4948263 |
| 1455422_x_at | *Sept4* | 8.270304 | 1.2998489 |
| 1448729_a_at | *Sept4* | 7.267432 | 1.7937915 |
| 1421092_at | *Serpina12* | 2.1796281 | 3.0403247 |
| 1416318_at | *Serpinb1a* | 3.5250423 | 3.3494034 |
| 1419149_at | *Serpine1* | 2.3903754 | 3.304353 |
| 1429029_at | *Sgms2* | 2.1556158 | 3.9280107 |
| 1448328_at | *Sh3bp2* | 2.162376 | 2.9698536 |
| 1420334_at | *Slc12a8* | 2.2518294 | 4.575999 |
| 1453133_at | *Slc25a31* | 8.594621 | 3.1359992 |
| 1422788_at | *Slc43a3* | 3.6979284 | 3.2262437 |
| 1418395_at | *Slc47a1* | 2.1903367 | 3.2031991 |
| 1439368_a_at | *Slc9a3r2* | 2.4359086 | 3.6909292 |
| 1448377_at | *Slpi* | 3.5049493 | 4.3751016 |
| 1460229_at | *Stag3* | 2.5133848 | 4.087729 |
| 1448956_at | *Stard10* | 2.729389 | 4.4194865 |
| 1434442_at | *Stbd1* | 4.8186717 | 4.087729 |
| 1429270_a_at | *Syce2* | 6.356512 | 2.6379287 |
| 1420433_at | *Taf7l* | 3.5147853 | 4.087729 |
| 1450555_at | *Tex13* | 8.916894 | 4.442827 |
| 1417482_at | *Tex19* | 39.345474 | 0.0 |
| 1437277_x_at | *Tgm2* | 2.3360982 | 4.484479 |
| 1455900_x_at | *Tgm2* | 2.1280818 | 4.390399 |
| 1433428_x_at | *Tgm2* | 2.3124783 | 2.5625594 |
| 1417500_a_at | *Tgm2* | 2.4879034 | 3.1804814 |
| 1422571_at | *Thbs2* | 2.6331732 | 3.25299 |
| 1419088_at | *Timp3* | 2.2151499 | 4.250691 |
| 1419089_at | *Timp3* | 3.3194087 | 4.390399 |
| 1449335_at | *Timp3* | 3.6108608 | 4.3669086 |
| 1457701_at | *Tmem136* | 2.126397 | 2.9731352 |
| 1433802_at | *Tmem151* | 2.280042 | 3.9568932 |
| 1424966_at | *Tmem40* | 5.7552257 | 3.755475 |
| 1452948_at | *Tnfaip8l2* | 2.0807586 | 3.7027807 |
| 1422303_a_at | *Tnfrsf18* | 3.148593 | 3.3754144 |
| 1450813_a_at | *Tnni1* | 2.721004 | 3.5172384 |
| 1419606_a_at | *Tnnt1* | 2.5998666 | 3.631157 |
| 1425028_a_at | *Tpm2* | 2.1043296 | 4.3193283 |
| 1419738_a_at | *Tpm2* | 2.060905 | 4.356351 |
| 1416713_at | *Tppp3* | 4.692691 | 3.9280107 |
| 1460226_at | *Trap1a* | 4.9921393 | 4.1236587 |
| 1426784_at | *Trim47* | 4.6452093 | 4.270932 |
| 1418398_a_at | *Tspan32* | 4.245654 | 3.3494034 |
| 1425157_x_at | *Tspan33* | 2.1076193 | 3.7897005 |
| 1455618_x_at | *Tspan33* | 2.4424202 | 3.5520625 |
| 1448296_x_at | *Tuba3a* | 3.2009814 | 3.929698 |
| 1452679_at | *Tubb2b* | 3.6214876 | 1.2998489 |
| 1448260_at | *Uchl1* | 13.535618 | 2.6379287 |
| 1448188_at | *Ucp2* | 2.6976566 | 3.3774319 |
| 1459740_s_at | *Ucp2* | 4.1698103 | 3.5591102 |
| 1419631_at | *Was* | 2.6818125 | 3.645918 |
| 1439373_x_at | *Wnt5b* | 2.0107906 | 3.8600578 |

Table S1 includes 290 probe sets representing 250 genes that were selected as statistically significant by the pairwise two-class SAM analysis (FDR and *Q*-values <0.05 and up-regulation after 5AzaCdR treatment more than 2-fold times). Fold-enrichments were calculated by comparing the expression values of treated cells to those of untreated cells.
