# Supplementary material for: Mining Gene Expression Signature for the Detection of Pre-Malignant Melanocytes and Early Melanomas with Risk for Metastasis
Source: PLoS One. 2012 Sep 11;7(9):e44800. doi: 10.1371/journal.pone.0044800 (PMC3439384; doi:10.1371/journal.pone.0044800)
Supplement: Table S2 — Transcripts Up-Regulated in 4C Pre-Malignant Melanocytes Following 5-aza-2′-deoxycytidine Treatment Identified by Genome-Wide Screening. (DOC) [file pone.0044800.s005.doc]

**Table S2. Transcripts Up-Regulated in 4C Pre-Malignant Melanocytes Following 5-aza-2'-deoxycytidine Treatment Identified by Genome-Wide Screening.**

| **Probe Set_ID** | **Gene** | **Fold-Enrichment** | ***Q*-Value (%)** |
| --- | --- | --- | --- |
| 1449383_at | *Adssl1* | 12.461346 | 4.7704067 |
| 1441054_at | *Apol2* | 5.003414 | 4.483661 |
| 1452257_at | *Bdh1* | 8.057388 | 2.3577874 |
| 1448595_a_at | *Bex1* | 176.88379 | 0.0 |
| 1420362_a_at | *Bik* | 5.476066 | 2.4203835 |
| 1427912_at | *Cbr3* | 34.44618 | 0.0 |
| 1420380_at | *Ccl2* | 27.935955 | 0.0 |
| 1418126_at | *Ccl5* | 15.932784 | 4.520716 |
| 1448919_at | *Cd302* | 17.54199 | 4.7483215 |
| 1425519_a_at | *Cd74* | 9.574924 | 4.520716 |
| 1452035_at | *Col4a1* | 6.8463306 | 2.9472342 |
| 1451191_at | *Crabp2* | 8.1325655 | 4.3760533 |
| 1457644_s_at | *Cxcl1* | 30.066854 | 2.5324383 |
| 1419209_at | *Cxcl1* | 149.53209 | 0.0 |
| 1449984_at | *Cxcl2* | 18.553127 | 2.0410695 |
| 1419542_at | *Dazl* | 125.73479 | 0.0 |
| 1449502_at | *Dazl* | 109.923294 | 0.0 |
| 1427242_at | *Ddx4* | 35.46157 | 0.0 |
| 1435493_at | *Dsp* | 18.202955 | 2.2418306 |
| 1435494_s_at | *Dsp* | 30.763813 | 0.0 |
| 1448931_at | *F2rl1* | 12.526919 | 2.7350333 |
| 1435910_at | *Fads3* | 4.4067407 | 4.7483215 |
| 1418773_at | *Fads3* | 8.052102 | 1.9260798 |
| 1423407_a_at | *Fbln2* | 10.835912 | 1.6677032 |
| 1417267_s_at | *Fkbp11* | 10.330634 | 4.090862 |
| 1416221_at | *Fstl1* | 18.336039 | 0.0 |
| 1448259_at | *Fstl1* | 19.431177 | 0.0 |
| 1417301_at | *Fzd6* | 10.490907 | 2.051275 |
| 1435906_x_at | *Gbp2* | 16.99515 | 2.58022 |
| 1418240_at | *Gbp2* | 18.092278 | 1.7532264 |
| 1418392_a_at | *Gbp3* | 14.312284 | 2.5965505 |
| 1429692_s_at | *Gch1* | 11.20726 | 1.935165 |
| 1420499_at | *Gch1* | 12.8995285 | 2.2791944 |
| 1450649_at | *Gng10* | 6.0961337 | 5.0276346 |
| 1436713_s_at | *Gtl2* | 4.857271 | 4.411344 |
| 1436543_at | *Gtpbp10* | 9.101105 | 2.0719948 |
| 1424067_at | *Icam1* | 67.76507 | 0.0 |
| 1416295_a_at | *Il2rg* | 10.48444 | 1.9819082 |
| 1440557_at | *Ipw* | 20.905668 | 2.681405 |
| 1417244_a_at | *Irf7* | 8.569688 | 1.9915291 |
| 1423227_at | *Krt17* | 9.640851 | 3.1079924 |
| 1423691_x_at | *Krt8* | 33.67855 | 0.0 |
| 1417777_at | *Ltb4dh* | 48.708492 | 0.0 |
| 1449328_at | *Ly75* | 6.37434 | 4.2734895 |
| 1436837_at | *Mael* | 31.93613 | 1.8993287 |
| 1415922_s_at | *Marcksl1* | 35.73514 | 2.1367447 |
| 1435415_x_at | *Marcksl1* | 6.4279 | 2.5965505 |
| 1437226_x_at | *Marcksl1* | 113.737686 | 0.0 |
| 1448428_at | *Nbl1* | 6.266026 | 1.9723798 |
| 1458299_s_at | *Nfkbie* | 6.483548 | 4.0221076 |
| 1416808_at | *Nid1* | 23.5079 | 2.681405 |
| 1423506_a_at | *Nnat* | 15.946353 | 2.205672 |
| 1420585_a_at | *Nxf2* | 16.637838 | 1.9260798 |
| 1424775_at | *Oas1a* | 9.185659 | 3.038926 |
| 1421566_at | *Pet2* | 9.887197 | 2.2791944 |
| 1429001_at | *Pir* | 15.701852 | 0.0 |
| 1449170_at | *Piwil2* | 19.454203 | 2.681405 |
| 1449799_s_at | *Pkp2* | 11.065875 | 2.5092049 |
| 1429183_at | *Pkp2* | 9.017127 | 2.1367447 |
| 1422962_a_at | *Psmb8* | 17.615192 | 2.681405 |
| 1426622_a_at | *Qpct* | 11.079199 | 2.5965505 |
| 1433906_at | *Rlbp1l1* | 13.738483 | 2.205672 |
| 1455197_at | *Rnd1* | 4.478206 | 4.883988 |
| 1423327_at | *Rpl39l* | 48.22105 | 0.0 |
| 1452730_at | *Rps4y2* | 54.45017 | 0.0 |
| 1427306_at | *Ryr1* | 9.204363 | 2.5092049 |
| 1439630_x_at | *Sbsn* | 4.7341843 | 4.883988 |
| 1453133_at | *Slc25a31* | 75.12739 | 0.0 |
| 1451139_at | *Slc39a4* | 10.504621 | 4.307139 |
| 1418395_at | *Slc47a1* | 9.69374 | 4.411344 |
| 1429817_at | *Sohlh2* | 6.314742 | 2.3852034 |
| 1416627_at | *Spint1* | 6.3804107 | 4.883988 |
| 1425317_x_at | *Stk31* | 21.138683 | 4.0221076 |
| 1454138_a_at | *Stk31* | 29.2553 | 1.7093958 |
| 1453228_at | *Stx11* | 14.220181 | 1.799364 |
| 1449534_at | *Sycp3* | 10.43648 | 2.8099656 |
| 1420433_at | *Taf7l* | 35.77552 | 0.0 |
| 1434960_at | *Taf9b* | 12.743989 | 4.7483215 |
| 1450555_at | *Tex13* | 15.761087 | 2.0719948 |
| 1417482_at | *Tex19* | 14.956181 | 2.5640936 |
| 1448123_s_at | *Tgfbi* | 21.028816 | 2.0719948 |
| 1415871_at | *Tgfbi* | 16.413416 | 2.0719948 |
| 1437463_x_at | *Tgfbi* | 23.867704 | 2.3178248 |
| 1456250_x_at | *Tgfbi* | 27.008135 | 0.0 |
| 1419089_at | *Timp3* | 16.123083 | 2.205672 |
| 1420064_s_at | *Tktl1* | 13.339749 | 0.0 |
| 1456981_at | *Tmc7* | 5.536365 | 4.307139 |
| 1416273_at | *Tnfaip2* | 7.1691737 | 2.3048034 |
| 1438855_x_at | *Tnfaip2* | 19.696869 | 0.0 |
| 1449577_x_at | *Tpm2* | 12.1145735 | 2.0719948 |
| 1419738_a_at | *Tpm2* | 10.061769 | 2.1147165 |
| 1460226_at | *Trap1a* | 29.931398 | 0.0 |
| 1452679_at | *Tubb2b* | 13.976318 | 1.9915291 |
| 1448260_at | *Uchl1* | 36.426575 | 0.0 |
| 1448162_at | *Vcam1* | 4.669705 | 4.9909368 |
| 1427262_at | *Xist* | 14.562756 | 0.0 |
| 1429947_a_at | *Zbp1* | 14.168666 | 0.0 |

Table S2 includes 97 probe sets representing 81 genes that were selected as statistically significant by the pairwise two-class SAM analysis (FDR and *Q*-values <0.05 and up-regulation after 5AzaCdR treatment more than 2-fold times). Fold-enrichments were calculated by comparing the expression values of treated cells to those of untreated cells.
