# Supplementary material for: Mining Gene Expression Signature for the Detection of Pre-Malignant Melanocytes and Early Melanomas with Risk for Metastasis
Source: PLoS One. 2012 Sep 11;7(9):e44800. doi: 10.1371/journal.pone.0044800 (PMC3439384; doi:10.1371/journal.pone.0044800)
Supplement: Table S3 — Transcripts Up-Regulated in 4C11− Non-Metastatic Melanoma Cells Following 5-aza-2′-deoxycytidine Treatment Identified by Genome-Wide Screening. (DOC) [file pone.0044800.s006.doc]

**Table S3. Transcripts Up-Regulated in 4C11- Non-Metastatic Melanoma Cells Following 5-aza-2'-deoxycytidine Treatment Identified by Genome-Wide Screening.**

| **Probe Set_ID** | **Gene** | **Fold-Enrichment** | ***Q*-Value (%)** |
| --- | --- | --- | --- |
| 1427371_at | *Abca8a* | 2.4808087 | 1.4908508 |
| 1419758_at | *Abcb1a* | 3.4568202 | 3.6017284 |
| 1428988_at | *Abcc3* | 2.0928304 | 4.2244697 |
| 1423570_at | *Abcg1* | 2.8444583 | 0.0 |
| 1419103_a_at | *Abhd6* | 2.5073442 | 3.7422473 |
| 1417094_at | *Acot7* | 2.1519012 | 3.2433412 |
| 1441975_at | *Acpp* | 2.1540966 | 3.2433412 |
| 1422428_at | *Acsbg1* | 2.387029 | 0.2916882 |
| 1422340_a_at | *Actg2* | 2.1218817 | 3.5340536 |
| 1416871_at | *Adam8* | 3.0378761 | 1.4291695 |
| 1435495_at | *Adora1* | 2.1921036 | 4.6506495 |
| 1423420_at | *Adrb1* | 3.1578894 | 0.0 |
| 1449383_at | *Adssl1* | 4.7856226 | 0.0 |
| 1424126_at | *Alas1* | 2.6190417 | 2.09057 |
| 1448789_at | *Aldh1a3* | 2.073018 | 0.92246395 |
| 1433769_at | *Als2cl* | 3.5077786 | 0.0 |
| 1439758_at | *Als2cr12* | 5.0733247 | 0.0 |
| 1417920_at | *Amn* | 4.3147492 | 0.0 |
| 1422573_at | *Ampd3* | 3.9192502 | 1.1254462 |
| 1417130_s_at | *Angptl4* | 8.477723 | 0.0 |
| 1448839_at | *Ankrd47* | 2.3362951 | 2.7721715 |
| 1460330_at | *Anxa3* | 15.626672 | 0.0 |
| 1417470_at | *Apobec3* | 2.2212796 | 0.8631242 |
| 1416677_at | *Apoh* | 3.6361399 | 1.3272862 |
| 1441054_at | *Apol2* | 3.2035859 | 0.0 |
| 1453080_at | *Apol3* | 4.0561748 | 0.5054597 |
| 1450460_at | *Aqp3* | 2.4619415 | 0.0 |
| 1436171_at | *Arhgap30* | 2.973736 | 0.7809219 |
| 1448660_at | *Arhgdig* | 2.6039069 | 0.4919808 |
| 1451340_at | *Arid5a* | 2.058143 | 3.5376544 |
| 1415780_a_at | *Armcx2* | 2.7513068 | 1.2587994 |
| 1456739_x_at | *Armcx2* | 5.7740073 | 0.0 |
| 1452474_a_at | *Art3* | 3.1880207 | 1.9066125 |
| 1419747_at | *Asgr2* | 2.86871 | 0.30494678 |
| 1449363_at | *Atf3* | 2.892088 | 3.402282 |
| 1452308_a_at | *Atp1a2* | 2.2462132 | 0.98835427 |
| 1443823_s_at | *Atp1a2* | 3.5336473 | 0.52712226 |
| 1427481_a_at | *Atp1a3* | 2.2255697 | 1.6799344 |
| 1434350_at | *Axud1* | 2.8753338 | 0.5020212 |
| 1418736_at | *B3galnt1* | 2.4456 | 3.6219444 |
| 1426329_s_at | *Baalc* | 2.5025346 | 1.1328505 |
| 1424951_at | *Baiap2l1* | 2.0743325 | 1.1328505 |
| 1428792_at | *Bcas1* | 2.1730146 | 3.6847382 |
| 1418133_at | *Bcl3* | 5.5027795 | 0.0 |
| 1452257_at | *Bdh1* | 3.2401328 | 0.3024472 |
| 1448595_a_at | *Bex1* | 91.199646 | 0.0 |
| 1449836_x_at | *Bik* | 2.4346151 | 2.34115 |
| 1420363_at | *Bik* | 2.2261868 | 0.98835427 |
| 1420362_a_at | *Bik* | 6.1663284 | 0.0 |
| 1421392_a_at | *Birc3* | 4.204936 | 0.0 |
| 1450759_at | *Bmp6* | 2.6044137 | 0.98835427 |
| 1449873_at | *Bmp8a* | 2.1890802 | 1.4637445 |
| 1440706_at | *Bmp8b* | 3.0488787 | 0.0 |
| 1450342_at | *Bmp8b* | 5.8104305 | 0.0 |
| 1417040_a_at | *Bok* | 2.2044864 | 1.4142305 |
| 1424921_at | *Brd4 | Bst2* | 2.842378 | 0.8977752 |
| 1435502_a_at | *Brdt* | 4.2033696 | 4.7396994 |
| 1450178_at | *Brdt* | 2.0773888 | 0.8661634 |
| 1449453_at | *Bst1* | 2.562605 | 2.8173776 |
| 1416051_at | *C2* | 2.096988 | 3.4188495 |
| 1423954_at | *C3* | 11.946939 | 0.0 |
| 1422815_at | *C9* | 2.34935 | 4.3510437 |
| 1426263_at | *Cadm4* | 4.609919 | 0.28493094 |
| 1433971_at | *Camta1* | 2.7871058 | 0.0 |
| 1433972_at | *Camta1* | 3.3481672 | 0.0 |
| 1428485_at | *Car12* | 2.0565977 | 1.3344867 |
| 1421001_a_at | *Car6* | 3.1803536 | 1.3442098 |
| 1449491_at | *Card10* | 6.6807494 | 0.52712226 |
| 1427912_at | *Cbr3* | 19.816095 | 0.0 |
| 1420039_s_at | *Cbx7* | 2.0042713 | 4.1404324 |
| 1418778_at | *Ccdc109b* | 2.1493802 | 3.5376544 |
| 1427205_x_at | *Ccdc46* | 2.1375694 | 2.8173776 |
| 1420380_at | *Ccl2* | 13.456784 | 0.0 |
| 1418126_at | *Ccl5* | 12.186187 | 0.0 |
| 1421228_at | *Ccl7* | 8.452437 | 0.0 |
| 1425658_at | *Cd109* | 2.3093526 | 2.7721715 |
| 1448919_at | *Cd302* | 5.649321 | 4.7396994 |
| 1460415_a_at | *Cd40* | 2.7552352 | 0.0 |
| 1449473_s_at | *Cd40* | 3.411531 | 0.0 |
| 1439221_s_at | *Cd40* | 3.7983868 | 0.0 |
| 1425519_a_at | *Cd74* | 12.10972 | 0.0 |
| 1424376_at | *Cdc42ep1* | 2.2873669 | 1.229952 |
| 1421679_a_at | *Cdkn1a* | 2.74927 | 0.68330663 |
| 1424638_at | *Cdkn1a* | 2.8522198 | 0.31809103 |
| 1449582_at | *Cdx1* | 2.3601077 | 1.3178056 |
| 1419833_s_at | *Centd3* | 3.560723 | 0.0 |
| 1435155_at | *Cgn* | 2.2349887 | 2.34115 |
| 1424529_s_at | *Cgref1* | 4.929906 | 0.0 |
| 1424528_at | *Cgref1* | 4.3228197 | 0.0 |
| 1451537_at | *Chi3l1* | 2.8704145 | 1.2484474 |
| 1420682_at | *Chrnb1* | 3.1169116 | 3.4188495 |
| 1428902_at | *Chst11* | 2.165712 | 1.2368232 |
| 1460569_x_at | *Cldn3* | 2.8244915 | 1.6610284 |
| 1434651_a_at | *Cldn3* | 2.0894098 | 1.4908508 |
| 1451701_x_at | *Cldn3* | 3.445206 | 0.79565626 |
| 1439427_at | *Cldn9* | 6.7959476 | 0.7809219 |
| 1441083_at | *Clptm1* | 2.0374842 | 1.7598677 |
| 1417917_at | *Cnn1* | 2.8673632 | 3.6034467 |
| 1426348_at | *Col4a1* | 3.609858 | 0.0 |
| 1452035_at | *Col4a1* | 6.321018 | 0.0 |
| 1424051_at | *Col4a2* | 3.17779 | 0.705068 |
| 1419703_at | *Col5a3* | 2.4086514 | 1.4908508 |
| 1434667_at | *Col8a2* | 2.4030643 | 1.3272862 |
| 1419527_at | *Comp* | 2.3574307 | 1.2368232 |
| 1460510_a_at | *Coq10b* | 2.1973093 | 0.76473695 |
| 1416246_a_at | *Coro1a* | 2.1842456 | 3.6034467 |
| 1455269_a_at | *Coro1a* | 2.6405802 | 0.5054597 |
| 1452367_at | *Coro2a* | 3.0798364 | 0.8601063 |
| 1453009_at | *Cpm* | 2.1285937 | 3.0157475 |
| 1429413_at | *Cpm* | 2.8315551 | 1.1328505 |
| 1417745_at | *Cpn1* | 2.3382645 | 3.402282 |
| 1451191_at | *Crabp2* | 5.9932723 | 0.0 |
| 1416326_at | *Crip1* | 3.0949533 | 0.49862918 |
| 1460458_at | *Crispld2* | 2.368423 | 1.8334688 |
| 1434758_at | *Crispld2* | 2.2326257 | 1.0178913 |
| 1437056_x_at | *Crispld2* | 2.7415793 | 0.76473695 |
| 1418476_at | *Crlf1* | 3.8273623 | 2.3092976 |
| 1449903_at | *Crtam* | 2.3295786 | 1.524209 |
| 1420686_at | *Cryba4* | 2.828554 | 1.6610284 |
| 1419872_at | *Csf1r* | 3.0239727 | 0.0 |
| 1450944_at | *Cspg4* | 2.0274916 | 2.09057 |
| 1423341_at | *Cspg4* | 3.0749152 | 0.0 |
| 1427797_s_at | *Ctse* | 3.454914 | 0.52712226 |
| 1418365_at | *Ctsh* | 2.6040199 | 1.8565313 |
| 1448591_at | *Ctss* | 2.9700801 | 3.2837124 |
| 1415803_at | *Cx3cl1* | 2.1399872 | 3.6017284 |
| 1457644_s_at | *Cxcl1* | 11.700752 | 0.0 |
| 1419209_at | *Cxcl1* | 65.0169 | 0.0 |
| 1418457_at | *Cxcl14* | 2.9443772 | 1.1133186 |
| 1454268_a_at | *Cyba* | 10.645645 | 0.0 |
| 1423630_at | *Cygb* | 2.856881 | 0.7473126 |
| 1429617_at | *Cyld* | 2.075711 | 1.3515955 |
| 1417590_at | *Cyp27a1* | 2.007625 | 1.4908508 |
| 1451453_at | *Dapk2* | 2.17544 | 4.752367 |
| 1419542_at | *Dazl* | 55.986816 | 0.7006056 |
| 1449502_at | *Dazl* | 42.59225 | 0.5347617 |
| 1424590_at | *Ddx19b* | 2.8915322 | 3.5593464 |
| 1427242_at | *Ddx4* | 16.671907 | 2.3092976 |
| 1436562_at | *Ddx58* | 2.3547742 | 3.6847382 |
| 1424303_at | *Depdc7* | 2.661451 | 0.98835427 |
| 1422678_at | *Dgat2* | 2.3252497 | 2.6080134 |
| 1422677_at | *Dgat2* | 2.1335607 | 2.1421514 |
| 1434959_at | *Dhh* | 2.0140495 | 3.2433412 |
| 1420768_a_at | *Dhx58* | 3.364989 | 3.2894902 |
| 1451426_at | *Dhx58* | 3.3980293 | 1.2626883 |
| 1454654_at | *Dirc2* | 7.6423154 | 2.1421514 |
| 1417787_at | *Dkkl1* | 2.1887333 | 3.5376544 |
| 1449939_s_at | *Dlk1* | 2.518273 | 1.2097888 |
| 1448877_at | *Dlx2* | 4.0747046 | 0.68330663 |
| 1421056_at | *Dnase1l3* | 3.0788133 | 1.2428988 |
| 1421057_at | *Dnase1l3* | 2.953682 | 0.66684145 |
| 1419674_a_at | *Dpep1* | 2.2481472 | 0.82685846 |
| 1429035_at | *Dpep3* | 2.8286626 | 3.4188495 |
| 1429597_at | *Dppa4* | 2.8154273 | 0.0 |
| 1435493_at | *Dsp* | 8.147672 | 0.0 |
| 1435494_s_at | *Dsp* | 16.523996 | 0.0 |
| 1431422_a_at | *Dusp14* | 2.7474697 | 2.5413578 |
| 1450698_at | *Dusp2* | 3.385022 | 0.0 |
| 1454737_at | *Dusp9* | 2.0275638 | 3.2894902 |
| 1416361_a_at | *Dync1i1* | 2.1205413 | 1.6799344 |
| 1453159_at | *Efhc1* | 2.376086 | 0.49862918 |
| 1448507_at | *Efhd1* | 3.075167 | 0.0 |
| 1417065_at | *Egr1* | 3.3749669 | 0.5054597 |
| 1423693_at | *Ela1* | 2.607902 | 3.5376544 |
| 1416916_at | *Elf3* | 2.397945 | 0.91559696 |
| 1435264_at | *Emilin2* | 4.2967434 | 0.69619924 |
| 1427484_at | *Eml5* | 2.0099704 | 4.2244697 |
| 1421097_at | *Endog* | 2.1883907 | 3.670333 |
| 1438317_a_at | *Endog* | 2.4656906 | 0.8764503 |
| 1418829_a_at | *Eno2* | 2.273091 | 0.8631242 |
| 1419276_at | *Enpp1* | 2.1527169 | 1.8334688 |
| 1435172_at | *Eomes* | 2.2879655 | 1.9826092 |
| 1426001_at | *Eomes* | 2.4287467 | 1.2587994 |
| 1434606_at | *Erbb3* | 2.3288286 | 0.91559696 |
| 1423232_at | *Etv4* | 2.2605603 | 1.8565313 |
| 1443381_at | *Etv4* | 2.5065622 | 0.29401243 |
| 1420998_at | *Etv5* | 2.0228655 | 4.752367 |
| 1435631_x_at | *Exoc6* | 2.027364 | 1.1975192 |
| 1423305_at | *Extl1* | 2.1273477 | 2.1421514 |
| 1448931_at | *F2rl1* | 8.546999 | 0.0 |
| 1416023_at | *Fabp3* | 2.4755082 | 3.4718556 |
| 1435910_at | *Fads3* | 3.533605 | 0.0 |
| 1418773_at | *Fads3* | 7.0018134 | 0.0 |
| 1460251_at | *Fas* | 2.125344 | 4.1404324 |
| 1449141_at | *Fblim1* | 3.763554 | 0.0 |
| 1418569_at | *Fblim1* | 4.519617 | 0.0 |
| 1423407_a_at | *Fbln2* | 7.231983 | 0.0 |
| 1449088_at | *Fbp2* | 2.5536475 | 0.8631242 |
| 1421746_a_at | *Fbxo17* | 2.8016477 | 2.1421514 |
| 1443698_at | *Fbxo39* | 3.5713081 | 1.4291695 |
| 1419322_at | *Fgd6* | 2.0533223 | 3.6847382 |
| 1435551_at | *Fhod3* | 2.6242678 | 2.7721715 |
| 1435482_at | *Fibcd1* | 2.1598446 | 1.2097888 |
| 1417267_s_at | *Fkbp11* | 4.412128 | 0.7978067 |
| 1425101_a_at | *Fkbp6* | 2.4906013 | 1.3515955 |
| 1447812_x_at | *Flnc* | 2.365098 | 0.84339565 |
| 1449073_at | *Flnc* | 2.5504334 | 0.6708829 |
| 1434739_at | *Fmr1nb* | 5.0049095 | 0.0 |
| 1417487_at | *Fosl1* | 9.063485 | 0.0 |
| 1417488_at | *Fosl1* | 9.0552025 | 0.0 |
| 1451752_at | *Foxk1* | 3.3309262 | 0.0 |
| 1435956_at | *Frmd5* | 2.3611455 | 0.4919808 |
| 1448378_at | *Fscn1* | 2.8183205 | 0.29998827 |
| 1416516_at | *Fscn1* | 2.6677442 | 0.32085702 |
| 1416514_a_at | *Fscn1* | 6.4228945 | 0.0 |
| 1416515_at | *Fscn1* | 3.1688082 | 0.0 |
| 1448259_at | *Fstl1* | 7.9372215 | 0.0 |
| 1416221_at | *Fstl1* | 8.01273 | 0.0 |
| 1422803_at | *Fstl3* | 5.041493 | 0.0 |
| 1455843_at | *Fut4* | 2.237698 | 3.6219444 |
| 1450135_at | *Fzd3* | 2.2492096 | 1.524209 |
| 1421157_at | *Fzd3* | 2.4880717 | 0.8601063 |
| 1449730_s_at | *Fzd3* | 2.7126355 | 0.8631242 |
| 1417301_at | *Fzd6* | 3.5863588 | 4.6506495 |
| 1449773_s_at | *Gadd45b* | 2.1092954 | 1.6799344 |
| 1450971_at | *Gadd45b* | 2.067663 | 1.4142305 |
| 1417177_at | *Galk1* | 5.85958 | 0.3153723 |
| 1417588_at | *Galnt3* | 2.0055058 | 1.8334688 |
| 1428816_a_at | *Gata2* | 2.8320873 | 0.0 |
| 1450333_a_at | *Gata2* | 3.266851 | 0.0 |
| 1418240_at | *Gbp2* | 6.1285167 | 0.8764503 |
| 1435906_x_at | *Gbp2* | 5.592326 | 0.8631242 |
| 1418392_a_at | *Gbp3* | 4.685016 | 1.9826092 |
| 1425156_at | *Gbp6* | 2.7427192 | 2.8659074 |
| 1420337_at | *Gbx2* | 2.311973 | 0.6708829 |
| 1429692_s_at | *Gch1* | 5.361692 | 0.0 |
| 1420499_at | *Gch1* | 8.679253 | 0.0 |
| 1435750_at | *Gchfr* | 2.6411195 | 4.523226 |
| 1424150_at | *Gdpd5* | 2.312581 | 3.6847382 |
| 1426063_a_at | *Gem* | 15.983963 | 0.7809219 |
| 1418753_at | *Gfpt2* | 2.5724819 | 0.5054597 |
| 1424927_at | *Glipr1* | 2.3575013 | 3.4188495 |
| 1435245_at | *Gls2* | 2.029441 | 2.8173776 |
| 1419194_s_at | *Gmfg* | 3.0425105 | 0.8065259 |
| 1419470_at | *Gnb4* | 2.1000347 | 4.523226 |
| 1450649_at | *Gng10* | 4.1945934 | 1.8137541 |
| 1419721_at | *Gpr109a* | 2.206462 | 3.5376544 |
| 1439489_at | *Gpr120* | 3.1366246 | 0.9065985 |
| 1444233_at | *Gpr132* | 2.3478 | 1.3154566 |
| 1417894_at | *Gpr97* | 2.6900792 | 0.71647686 |
| 1437486_at | *Gprc5a* | 2.4625096 | 1.3515955 |
| 1417836_at | *Gpx7* | 2.5506284 | 3.4188495 |
| 1434725_at | *Gramd1c* | 4.8040643 | 0.0 |
| 1427046_at | *Grhl2* | 8.748955 | 0.0 |
| 1425891_a_at | *Grtp1* | 2.036611 | 1.4291695 |
| 1428767_at | *Gsdmdc1* | 2.1699588 | 0.8506872 |
| 1418186_at | *Gstt1* | 2.5175428 | 2.6080134 |
| 1421358_at | *H2-M3* | 3.649328 | 0.0 |
| 1419297_at | *H2-Oa* | 2.1166997 | 1.3515955 |
| 1424171_a_at | *Hagh* | 2.0906744 | 3.2344759 |
| 1419196_at | *Hamp1* | 4.237106 | 3.5593464 |
| 1436643_x_at | *Hamp2* | 2.4543312 | 0.88485754 |
| 1416997_a_at | *Hap1* | 2.0317724 | 0.88485754 |
| 1438037_at | *Herc5* | 3.0600584 | 4.1404324 |
| 1453757_at | *Herc5* | 3.0888932 | 3.6847382 |
| 1432026_a_at | *Herc5* | 3.244923 | 1.0086167 |
| 1449226_at | *Hic1* | 2.0193331 | 3.402282 |
| 1458802_at | *Hivep3* | 2.2189004 | 2.6080134 |
| 1416184_s_at | *Hmga1* | 2.9094868 | 0.0 |
| 1428242_at | *Hmha1* | 2.1458457 | 2.254912 |
| 1451776_s_at | *Hod* | 2.966825 | 0.9065985 |
| 1428662_a_at | *Hod* | 2.950955 | 0.29401243 |
| 1452317_at | *Hoxb9* | 2.1310856 | 2.3092976 |
| 1422239_at | *Hoxd13* | 2.1164129 | 2.284433 |
| 1440626_at | *Hoxd13* | 3.3205547 | 1.9066125 |
| 1451611_at | *Hrasls3* | 2.1021397 | 1.8137541 |
| 1445597_s_at | *Hrasls3* | 2.0738924 | 1.4142305 |
| 1416761_at | *Hsd11b2* | 2.0818002 | 3.5376544 |
| 1428640_at | *Hsf2bp* | 2.4288018 | 1.1133186 |
| 1442130_at | *Hsh2d* | 3.7059114 | 0.71647686 |
| 1452388_at | *Hspa1a* | 5.170445 | 0.8661634 |
| 1422943_a_at | *Hspb1* | 5.7106037 | 0.0 |
| 1425964_x_at | *Hspb1* | 6.1038194 | 0.0 |
| 1422196_at | *Htr5b* | 2.4490943 | 0.0 |
| 1431049_at | *Ica1l* | 2.2071278 | 1.8565313 |
| 1424067_at | *Icam1* | 31.931234 | 0.0 |
| 1445897_s_at | *Ifi35* | 2.1369727 | 4.6506495 |
| 1424617_at | *Ifi35* | 2.2839956 | 2.120607 |
| 1423555_a_at | *Ifi44* | 3.520703 | 3.670333 |
| 1417292_at | *Ifi47* | 3.3107927 | 2.1421514 |
| 1427216_at | *Ifnz* | 2.6567292 | 4.1404324 |
| 1417141_at | *Igtp* | 3.106451 | 0.98835427 |
| 1421291_at | *Il18rap* | 2.3984647 | 1.6799344 |
| 1419532_at | *Il1r2* | 2.1097198 | 4.7396994 |
| 1425145_at | *Il1rl1* | 2.410639 | 4.523226 |
| 1422317_a_at | *Il1rl1* | 2.7185652 | 2.3583634 |
| 1416296_at | *Il2rg* | 3.3479607 | 0.2703191 |
| 1416295_a_at | *Il2rg* | 11.5238495 | 0.28493094 |
| 1450297_at | *Il6* | 5.0947022 | 2.1421514 |
| 1418110_a_at | *Inpp5d* | 3.027828 | 0.7809219 |
| 1458595_at | *Intu* | 2.9198844 | 1.6610284 |
| 1436507_at | *Irak2* | 2.0373292 | 1.0225929 |
| 1448436_a_at | *Irf1* | 2.076037 | 3.3147643 |
| 1460231_at | *Irf5* | 3.2674806 | 1.229952 |
| 1418301_at | *Irf6* | 3.0604064 | 0.0 |
| 1417244_a_at | *Irf7* | 6.3098927 | 0.2916882 |
| 1448452_at | *Irf8* | 2.2319224 | 3.8976216 |
| 1416714_at | *Irf8* | 2.7478492 | 2.1421514 |
| 1419569_a_at | *Isg20* | 4.072976 | 0.0 |
| 1415977_at | *Isyna1* | 4.3237696 | 0.0 |
| 1450029_s_at | *Itga9* | 2.0482588 | 4.7396994 |
| 1426431_at | *Jag2* | 2.9040585 | 0.27332267 |
| 1415899_at | *Junb* | 6.3542347 | 0.0 |
| 1435945_a_at | *Kcnn4* | 3.4520347 | 0.7473126 |
| 1421038_a_at | *Kcnn4* | 3.000834 | 0.28059742 |
| 1455266_at | *Kif5c* | 3.148826 | 2.3092976 |
| 1416029_at | *Klf10* | 2.417543 | 3.5593464 |
| 1448932_at | *Krt16* | 4.136733 | 0.0 |
| 1423227_at | *Krt17* | 17.306936 | 0.0 |
| 1423691_x_at | *Krt8* | 15.968355 | 0.0 |
| 1419619_at | *Krt80* | 2.3231525 | 0.76473695 |
| 1418449_at | *Lad1* | 2.1443017 | 3.6847382 |
| 1418153_at | *Lama1* | 5.1430645 | 0.0 |
| 1424114_s_at | *Lamb1-1* | 3.3032842 | 0.0 |
| 1417812_a_at | *Lamb3* | 2.9268222 | 2.7721715 |
| 1421279_at | *Lamc2* | 2.3434544 | 1.4613291 |
| 1425396_a_at | *Lck* | 2.2346766 | 2.09057 |
| 1427747_a_at | *Lcn2* | 4.021423 | 0.0 |
| 1417638_at | *Lefty1* | 2.1518357 | 0.98835427 |
| 1426808_at | *Lgals3* | 2.1339552 | 4.93483 |
| 1421207_at | *Lif* | 5.0390396 | 0.7511157 |
| 1444003_at | *Lincr* | 2.177127 | 2.9105732 |
| 1457026_at | *Liph* | 2.3130953 | 1.2097888 |
| 1450134_at | *Loxl4* | 2.1104693 | 1.2626883 |
| 1421153_at | *Loxl4* | 2.4770453 | 0.27433872 |
| 1449440_at | *Lpin3* | 2.7899828 | 2.9105732 |
| 1448487_at | *Lrrfip1* | 2.206886 | 0.7809219 |
| 1417777_at | *Ltb4dh* | 23.075512 | 0.0 |
| 1418061_at | *Ltbp2* | 2.4374852 | 1.3178056 |
| 1436837_at | *Mael* | 13.662597 | 0.8065259 |
| 1418936_at | *Maff* | 3.7790012 | 0.82225204 |
| 1434364_at | *Map3k14* | 2.4702775 | 0.88485754 |
| 1449283_a_at | *Mapk12* | 2.3802814 | 4.3510437 |
| 1435415_x_at | *Marcksl1* | 5.542196 | 0.0 |
| 1415922_s_at | *Marcksl1* | 28.916834 | 0.0 |
| 1437226_x_at | *Marcksl1* | 194.59848 | 0.0 |
| 1428223_at | *Mfsd2* | 2.2551045 | 1.2921674 |
| 1455531_at | *Mfsd4* | 2.3077345 | 1.2097888 |
| 1450391_a_at | *Mgll* | 2.2401154 | 3.6034467 |
| 1417281_a_at | *Mmp23* | 2.2984107 | 0.5054597 |
| 1416298_at | *Mmp9* | 2.6603181 | 1.0225929 |
| 1448291_at | *Mmp9* | 3.153074 | 0.0 |
| 1455099_at | *Mogat2* | 2.4726634 | 3.2894902 |
| 1419418_a_at | *Morc1* | 3.0782928 | 0.0 |
| 1419340_at | *Mov10l1* | 4.051933 | 0.81543773 |
| 1427076_at | *Mpeg1* | 3.5343246 | 1.9826092 |
| 1449590_a_at | *Mras* | 2.2807434 | 0.8764503 |
| 1428942_at | *Mt2* | 4.821757 | 0.0 |
| 1454904_at | *Mtm1* | 3.2799842 | 3.402282 |
| 1427025_at | *Mtmr7* | 2.12306 | 2.9105732 |
| 1447831_s_at | *Mtmr7* | 4.0628734 | 0.0 |
| 1451905_a_at | *Mx1* | 2.8489213 | 3.5340536 |
| 1419676_at | *Mx2* | 2.6461103 | 1.4637445 |
| 1434777_at | *Mycl1* | 2.052743 | 1.4637445 |
| 1455694_at | *Nbeal2* | 2.0694025 | 0.88485754 |
| 1448428_at | *Nbl1* | 3.1239812 | 0.0 |
| 1433720_s_at | *Ndg2* | 2.4590385 | 1.524209 |
| 1436990_s_at | *Ndg2* | 3.477019 | 0.0 |
| 1436188_a_at | *Ndrg4* | 3.1923885 | 1.9066125 |
| 1426615_s_at | *Ndrg4* | 3.5344567 | 1.3515955 |
| 1439205_at | *Nfatc2* | 3.2136927 | 2.6080134 |
| 1417520_at | *Nfe2l3* | 2.5982518 | 3.4188495 |
| 1453614_a_at | *Nfe2l3* | 2.241436 | 1.4908508 |
| 1429128_x_at | *Nfkb2* | 2.237112 | 1.229952 |
| 1425902_a_at | *Nfkb2* | 3.3898687 | 0.0 |
| 1420088_at | *Nfkbia* | 3.2022336 | 1.2484474 |
| 1438157_s_at | *Nfkbia* | 3.3683705 | 0.8661634 |
| 1449731_s_at | *Nfkbia* | 3.8906014 | 0.76473695 |
| 1420089_at | *Nfkbia* | 3.253596 | 0.5054597 |
| 1448306_at | *Nfkbia* | 4.6035123 | 0.0 |
| 1421266_s_at | *Nfkbib* | 2.3759365 | 2.3583634 |
| 1458299_s_at | *Nfkbie* | 5.762414 | 0.0 |
| 1431843_a_at | *Nfkbie* | 4.5497046 | 0.0 |
| 1417483_at | *Nfkbiz* | 2.4519777 | 4.3510437 |
| 1448728_a_at | *Nfkbiz* | 2.3808017 | 2.1421514 |
| 1448978_at | *Ngef* | 3.2820966 | 0.0 |
| 1454903_at | *Ngfr* | 2.8883483 | 0.5054597 |
| 1454114_a_at | *Nhedc1* | 2.2365062 | 1.229952 |
| 1430233_a_at | *Nhedc1* | 2.716931 | 0.7211445 |
| 1422567_at | *Niban* | 2.4890912 | 0.30877456 |
| 1416808_at | *Nid1* | 16.232115 | 0.27433872 |
| 1449566_at | *Nkx2-5* | 2.1398888 | 0.8661634 |
| 1433661_at | *Nlrx1* | 3.1073139 | 0.27433872 |
| 1423506_a_at | *Nnat* | 8.163159 | 0.0 |
| 1416474_at | *Nope* | 3.2698812 | 1.2921674 |
| 1441075_at | *Nostrin* | 2.0335789 | 1.3272862 |
| 1452107_s_at | *Npnt* | 2.5371094 | 1.2428988 |
| 1452106_at | *Npnt* | 2.428943 | 0.30494678 |
| 1450791_at | *Nppb* | 8.491775 | 0.0 |
| 1434877_at | *Nptx1* | 2.8685777 | 0.82685846 |
| 1438796_at | *Nr4a3* | 2.2448068 | 0.7511157 |
| 1417985_at | *Nrarp* | 2.1391132 | 1.3442098 |
| 1429049_at | *Nuak2* | 3.2356243 | 0.5347617 |
| 1420585_a_at | *Nxf2* | 8.129396 | 1.0225929 |
| 1424775_at | *Oas1a* | 4.1262946 | 0.0 |
| 1418686_at | *Oas1c* | 2.292008 | 0.76473695 |
| 1425065_at | *Oas2* | 2.758203 | 0.9839616 |
| 1425374_at | *Oas3* | 5.982164 | 0.0 |
| 1424339_at | *Oasl1* | 3.4754198 | 4.752367 |
| 1424359_at | *Oplah* | 2.9970107 | 0.0 |
| 1456495_s_at | *Osbpl6* | 2.648608 | 0.691848 |
| 1418675_at | *Osmr* | 4.5229845 | 0.0 |
| 1418674_at | *Osmr* | 30.15093 | 0.0 |
| 1418272_at | *Oxct2a* | 2.4930394 | 3.4718556 |
| 1422218_at | *P2rx7* | 2.6722686 | 3.6847382 |
| 1424733_at | *P2ry14* | 2.095301 | 1.1902761 |
| 1419767_at | *Padi3* | 3.4197524 | 3.4188495 |
| 1420979_at | *Pak1* | 2.6361353 | 2.3583634 |
| 1426774_at | *Parp12* | 2.736602 | 3.5340536 |
| 1451564_at | *Parp14* | 3.015587 | 4.0998397 |
| 1416897_at | *Parp9* | 2.4474893 | 3.2837124 |
| 1419271_at | *Pax6* | 2.3765726 | 2.8173776 |
| 1424793_a_at | *Pbp2* | 2.0576985 | 0.9839616 |
| 1450414_at | *Pdgfb* | 2.3113034 | 3.3147643 |
| 1450413_at | *Pdgfb* | 2.3384721 | 0.98835427 |
| 1450962_at | *Pdha2* | 2.228751 | 2.8173776 |
| 1417273_at | *Pdk4* | 3.5355961 | 0.8661634 |
| 1417928_at | *Pdlim4* | 10.912248 | 0.0 |
| 1417959_at | *Pdlim7* | 2.5149398 | 4.7396994 |
| 1416271_at | *Perp* | 5.1505537 | 3.4188495 |
| 1421566_at | *Pet2* | 8.003298 | 0.28493094 |
| 1417837_at | *Phlda2* | 2.8877697 | 2.284433 |
| 1429001_at | *Pir* | 4.3471346 | 0.0 |
| 1418809_at | *Pira2* | 2.5016358 | 0.83622795 |
| 1449170_at | *Piwil2* | 4.215129 | 0.0 |
| 1449586_at | *Pkp1* | 2.1398454 | 1.8334688 |
| 1429183_at | *Pkp2* | 3.3388362 | 0.0 |
| 1449799_s_at | *Pkp2* | 6.179411 | 0.0 |
| 1418831_at | *Pkp3* | 2.2555516 | 0.92246395 |
| 1451335_at | *Plac8* | 3.3596663 | 0.0 |
| 1452521_a_at | *Plaur* | 2.5568426 | 0.30877456 |
| 1437893_at | *Plb1* | 2.0721745 | 3.2344759 |
| 1448432_at | *Plcd1* | 2.0675538 | 4.0998397 |
| 1416675_s_at | *Plcd1* | 2.13144 | 3.670333 |
| 1449424_at | *Plek2* | 2.4810734 | 1.9826092 |
| 1427005_at | *Plk2* | 2.0785732 | 2.8173776 |
| 1418090_at | *Plvap* | 2.244548 | 0.5054597 |
| 1451475_at | *Plxnd1* | 2.496792 | 1.4291695 |
| 1429667_at | *Pou4f1* | 2.4871073 | 3.4718556 |
| 1453127_at | *Ppm1j* | 2.341901 | 0.7118693 |
| 1444480_at | *Prkag3* | 2.1020555 | 3.2433412 |
| 1434325_x_at | *Prkar1b* | 6.8420277 | 0.0 |
| 1418085_at | *Prkcz* | 2.0043774 | 3.0157475 |
| 1420664_s_at | *Procr* | 3.5896513 | 0.0 |
| 1456543_at | *Prokr1* | 8.3693 | 0.0 |
| 1422530_at | *Prph* | 2.651814 | 1.6799344 |
| 1420352_at | *Prss22* | 3.2165 | 0.0 |
| 1460437_at | *Pscd4* | 2.5713398 | 0.0 |
| 1422962_a_at | *Psmb8* | 8.143138 | 0.0 |
| 1424560_at | *Pstpip1* | 2.0561745 | 3.6219444 |
| 1432162_s_at | *Ptar1* | 2.0264544 | 1.3178056 |
| 1449449_at | *Ptges* | 2.3663988 | 0.84339565 |
| 1439747_at | *Ptges* | 3.0386 | 0.7006056 |
| 1449450_at | *Ptges* | 4.2586007 | 0.0 |
| 1427313_at | *Ptgir* | 2.869969 | 3.7422473 |
| 1448816_at | *Ptgis* | 2.5094047 | 0.0 |
| 1423414_at | *Ptgs1* | 2.0471728 | 3.2344759 |
| 1436448_a_at | *Ptgs1* | 2.0775125 | 2.6080134 |
| 1417262_at | *Ptgs2* | 2.5027812 | 0.7211445 |
| 1452589_at | *Ptk7* | 2.3648453 | 1.2921674 |
| 1418181_at | *Ptp4a3* | 2.093093 | 0.7511157 |
| 1418539_a_at | *Ptpre* | 2.2280128 | 0.705068 |
| 1418666_at | *Ptx3* | 3.141068 | 2.254912 |
| 1426622_a_at | *Qpct* | 4.315749 | 0.0 |
| 1417481_at | *Ramp1* | 2.1751623 | 0.91559696 |
| 1428443_a_at | *Rap1gap* | 2.0691059 | 3.5340536 |
| 1417333_at | *Rasa4* | 2.472246 | 2.6080134 |
| 1427975_at | *Rasl10a* | 2.6752613 | 0.5233838 |
| 1444009_at | *Rassf4* | 4.0016546 | 0.0 |
| 1422637_at | *Rassf5* | 3.404904 | 0.0 |
| 1422638_s_at | *Rassf5* | 3.3233082 | 0.0 |
| 1433683_at | *Rbm35b* | 2.5489748 | 3.6847382 |
| 1417856_at | *Relb* | 3.891691 | 0.7511157 |
| 1436359_at | *Ret* | 2.0712779 | 2.34115 |
| 1455265_a_at | *Rgs16* | 3.2174466 | 1.2484474 |
| 1451452_a_at | *Rgs16* | 2.3603053 | 1.2097888 |
| 1426037_a_at | *Rgs16* | 2.9698699 | 0.6708829 |
| 1442819_at | *Rhbdl2* | 3.039746 | 0.8977752 |
| 1417049_at | *Rhd* | 2.783116 | 0.82685846 |
| 1424976_at | *Rhov* | 2.3907874 | 0.5347617 |
| 1419018_at | *Rhox6* | 2.387927 | 3.0157475 |
| 1448449_at | *Ripk3* | 12.31259 | 0.0 |
| 1420459_at | *Ripply3* | 2.3266835 | 4.7396994 |
| 1433906_at | *Rlbp1l1* | 4.2800264 | 0.68330663 |
| 1455197_at | *Rnd1* | 3.4677997 | 0.0 |
| 1429399_at | *Rnf125* | 2.6136553 | 2.9105732 |
| 1425124_at | *Rnf183* | 2.4728386 | 0.0 |
| 1455500_at | *Rnf213* | 2.1217985 | 3.5340536 |
| 1448996_at | *Rom1* | 2.275275 | 3.4718556 |
| 1423327_at | *Rpl39l* | 11.336532 | 0.0 |
| 1452730_at | *Rps4y2* | 35.093166 | 0.0 |
| 1422562_at | *Rrad* | 3.4852705 | 0.0 |
| 1418580_at | *Rtp4* | 2.8604202 | 3.6219444 |
| 1434743_x_at | *Rusc1* | 2.0926914 | 4.3510437 |
| 1427306_at | *Ryr1* | 7.322548 | 0.0 |
| 1421856_at | *S100a3* | 2.0822453 | 4.752367 |
| 1458308_at | *Sbno2* | 2.2120333 | 2.254912 |
| 1441840_x_at | *Sbno2* | 2.0030456 | 1.2073148 |
| 1459897_a_at | *Sbsn* | 4.1814017 | 0.0 |
| 1439630_x_at | *Sbsn* | 4.267864 | 0.0 |
| 1459898_at | *Sbsn* | 3.1109245 | 0.0 |
| 1434740_at | *Scarf2* | 2.331069 | 2.1421514 |
| 1458813_at | *Scn5a* | 2.3835359 | 0.8764503 |
| 1423009_at | *Sec1* | 3.4861078 | 0.6708829 |
| 1455784_at | *Sec1* | 5.3119774 | 0.0 |
| 1425002_at | *Sectm1a* | 2.99057 | 0.0 |
| 1448110_at | *Sema4a* | 2.09578 | 1.4908508 |
| 1439768_x_at | *Sema4f* | 3.1485062 | 1.2484474 |
| 1439833_at | *Sept3* | 2.0444634 | 3.0157475 |
| 1455422_x_at | *Sept4* | 2.4470918 | 1.8334688 |
| 1448729_a_at | *Sept4* | 2.5465534 | 1.4908508 |
| 1421092_at | *Serpina12* | 2.6529121 | 0.7118693 |
| 1424923_at | *Serpina3g* | 2.3808842 | 1.2626883 |
| 1422804_at | *Serpinb6b* | 2.3033884 | 4.752367 |
| 1418422_at | *Serpinb9g* | 10.23484 | 0.0 |
| 1419149_at | *Serpine1* | 4.2949944 | 0.0 |
| 1448443_at | *Serpini1* | 4.4014463 | 0.5054597 |
| 1416702_at | *Serpini1* | 7.39661 | 0.0 |
| 1447901_x_at | *Sfi1* | 2.370848 | 1.4142305 |
| 1452195_s_at | *Sfi1* | 2.0996592 | 0.9065985 |
| 1426787_at | *Sfi1* | 2.730958 | 0.76473695 |
| 1429029_at | *Sgms2* | 2.3961031 | 1.9826092 |
| 1448328_at | *Sh3bp2* | 3.1251445 | 0.8977752 |
| 1432103_a_at | *Sh3gl3* | 2.0999508 | 3.6034467 |
| 1449163_at | *Sigirr* | 2.0178533 | 3.2837124 |
| 1452214_at | *Skil* | 2.0117648 | 3.5593464 |
| 1422054_a_at | *Skil* | 7.2705617 | 0.28493094 |
| 1425294_at | *Slamf8* | 3.1917174 | 2.8173776 |
| 1433578_at | *Slc10a4* | 3.3340862 | 4.2244697 |
| 1420361_at | *Slc11a1* | 4.9168363 | 0.0 |
| 1420697_at | *Slc15a3* | 3.749252 | 1.0086167 |
| 1436368_at | *Slc16a10* | 2.5140886 | 0.7211445 |
| 1430629_at | *Slc16a14* | 2.0695179 | 0.97960776 |
| 1424735_at | *Slc25a25* | 2.010989 | 1.2097888 |
| 1453133_at | *Slc25a31* | 24.044622 | 0.52712226 |
| 1416639_at | *Slc2a5* | 2.01165 | 3.4718556 |
| 1434015_at | *Slc2a6* | 2.000606 | 1.9826092 |
| 1422786_at | *Slc30a1* | 2.2566266 | 4.0998397 |
| 1436164_at | *Slc30a1* | 2.111156 | 1.1975192 |
| 1428642_at | *Slc35d3* | 2.764435 | 0.8601063 |
| 1428111_at | *Slc38a4* | 2.3886724 | 4.0998397 |
| 1448889_at | *Slc38a4* | 2.2705007 | 0.8977752 |
| 1451139_at | *Slc39a4* | 6.290667 | 0.29401243 |
| 1426663_s_at | *Slc45a3* | 2.427961 | 0.858106 |
| 1418395_at | *Slc47a1* | 4.647232 | 0.0 |
| 1439368_a_at | *Slc9a3r2* | 3.281946 | 1.2626883 |
| 1427102_at | *Slfn4* | 4.8866553 | 2.8173776 |
| 1436472_at | *Slfn9* | 2.4776278 | 0.2703191 |
| 1448377_at | *Slpi* | 2.8030922 | 0.0 |
| 1415935_at | *Smoc2* | 2.8204622 | 1.9066125 |
| 1424268_at | *Smox* | 2.3324995 | 0.2703191 |
| 1428396_at | *Smurf1* | 2.0948918 | 1.7598677 |
| 1438470_at | *Socs2* | 4.005065 | 1.7598677 |
| 1429817_at | *Sohlh2* | 3.673308 | 1.1254462 |
| 1416564_at | *Sox7* | 2.0234776 | 4.523226 |
| 1451596_a_at | *Sphk1* | 2.320019 | 0.7511157 |
| 1460407_at | *Spib* | 3.8544943 | 3.670333 |
| 1454710_at | *Spink2* | 2.816161 | 0.0 |
| 1416627_at | *Spint1* | 5.212034 | 0.6708829 |
| 1417426_at | *Srgn* | 3.2636743 | 4.3510437 |
| 1417616_at | *St6galnac2* | 2.3460271 | 1.0086167 |
| 1424581_at | *Stac2* | 3.2705147 | 1.3154566 |
| 1460229_at | *Stag3* | 4.1052637 | 0.28493094 |
| 1418546_a_at | *Stambpl1* | 2.2086518 | 1.4908508 |
| 1448956_at | *Stard10* | 2.5708 | 4.752367 |
| 1450033_a_at | *Stat1* | 2.4836133 | 1.2626883 |
| 1425317_x_at | *Stk31* | 8.271307 | 2.9105732 |
| 1422723_at | *Stra6* | 3.7128973 | 0.0 |
| 1457780_at | *Stx11* | 2.754928 | 0.92246395 |
| 1453228_at | *Stx11* | 14.830583 | 0.7978067 |
| 1417335_at | *Sult2b1* | 2.2333734 | 0.97960776 |
| 1453715_at | *Sv2c* | 2.0941575 | 0.88485754 |
| 1429270_a_at | *Syce2* | 13.120039 | 0.0 |
| 1444122_at | *Sycp2* | 2.147693 | 0.8065259 |
| 1449534_at | *Sycp3* | 5.348801 | 0.29401243 |
| 1420958_at | *Sys1* | 2.2678092 | 0.9065985 |
| 1460081_at | *Syt7* | 2.4026103 | 3.3147643 |
| 1441927_at | *Syt7* | 2.190283 | 1.2428988 |
| 1416579_a_at | *Tacstd1* | 4.1619673 | 0.0 |
| 1420433_at | *Taf7l* | 16.754772 | 0.0 |
| 1434960_at | *Taf9b* | 6.7038503 | 3.5593464 |
| 1421812_at | *Tapbp* | 2.158478 | 2.8173776 |
| 1449361_at | *Tbx21* | 3.8806741 | 0.0 |
| 1424531_a_at | *Tcea3* | 2.787445 | 1.0178913 |
| 1449592_at | *Tcf15* | 2.669628 | 1.8137541 |
| 1435205_at | *Tcfap2e* | 2.5411568 | 0.68330663 |
| 1434425_at | *Tchh* | 2.5091949 | 0.30877456 |
| 1426716_at | *Tdrd7* | 2.0155575 | 1.2921674 |
| 1453357_at | *Tdrd9* | 2.9565167 | 0.0 |
| 1453366_at | *Tdrkh* | 2.6140845 | 0.27433872 |
| 1419729_at | *Tex11* | 4.7640405 | 1.9826092 |
| 1421183_at | *Tex12* | 6.4426494 | 1.4637445 |
| 1432159_a_at | *Tex13* | 3.0058455 | 0.27433872 |
| 1450555_at | *Tex13* | 14.016258 | 0.0 |
| 1417482_at | *Tex19* | 29.179243 | 0.0 |
| 1451790_a_at | *Tfpi* | 2.2110386 | 1.6610284 |
| 1451791_at | *Tfpi* | 2.0288417 | 1.229952 |
| 1418547_at | *Tfpi2* | 9.468564 | 2.7721715 |
| 1415871_at | *Tgfbi* | 9.8708725 | 0.0 |
| 1448123_s_at | *Tgfbi* | 13.184513 | 0.0 |
| 1437463_x_at | *Tgfbi* | 10.665405 | 0.0 |
| 1456250_x_at | *Tgfbi* | 18.4498 | 0.0 |
| 1451416_a_at | *Tgm1* | 2.6642075 | 1.2368232 |
| 1460227_at | *Timp1* | 2.0641727 | 4.752367 |
| 1419088_at | *Timp3* | 9.575317 | 0.0 |
| 1449334_at | *Timp3* | 8.528434 | 0.0 |
| 1419089_at | *Timp3* | 13.935693 | 0.0 |
| 1449335_at | *Timp3* | 20.610916 | 0.0 |
| 1449567_at | *Tktl1* | 2.7983582 | 1.8334688 |
| 1420064_s_at | *Tktl1* | 4.5665073 | 1.4637445 |
| 1449049_at | *Tlr1* | 2.4881444 | 3.6017284 |
| 1419132_at | *Tlr2* | 2.0157444 | 1.1975192 |
| 1456981_at | *Tmc7* | 2.851589 | 1.1975192 |
| 1455148_at | *Tmem130* | 2.594811 | 0.0 |
| 1433579_at | *Tmem30b* | 4.265249 | 1.3344867 |
| 1451546_s_at | *Tmem40* | 2.8328524 | 1.6799344 |
| 1441917_s_at | *Tmem40* | 3.3464885 | 0.0 |
| 1424966_at | *Tmem40* | 3.8696685 | 0.0 |
| 1417895_a_at | *Tmem54* | 2.662704 | 0.27433872 |
| 1452825_at | *Tmem59l* | 3.2049747 | 0.30494678 |
| 1453285_at | *Tmem88* | 2.5881367 | 2.8659074 |
| 1422705_at | *Tmepai* | 2.6521957 | 0.3024472 |
| 1419607_at | *Tnf* | 2.1597168 | 0.76473695 |
| 1416273_at | *Tnfaip2* | 7.1429005 | 0.0 |
| 1438855_x_at | *Tnfaip2* | 22.76194 | 0.0 |
| 1433699_at | *Tnfaip3* | 3.1142042 | 2.1421514 |
| 1418571_at | *Tnfrsf12a* | 2.387215 | 0.81543773 |
| 1418572_x_at | *Tnfrsf12a* | 2.4779415 | 0.7211445 |
| 1419307_at | *Tnfrsf13c* | 4.967258 | 0.28059742 |
| 1418099_at | *Tnfrsf1b* | 4.309948 | 0.0 |
| 1426095_a_at | *Tnfrsf22* | 2.2954044 | 4.6506495 |
| 1422101_at | *Tnfrsf23* | 2.7545836 | 0.0 |
| 1460469_at | *Tnfrsf9* | 3.0183892 | 0.0 |
| 1428034_a_at | *Tnfrsf9* | 6.924344 | 0.0 |
| 1422924_at | *Tnfsf9* | 2.0137365 | 3.5593464 |
| 1427689_a_at | *Tnip1* | 2.280462 | 0.69619924 |
| 1450813_a_at | *Tnni1* | 3.3309424 | 0.8631242 |
| 1422536_at | *Tnni3* | 2.3408792 | 0.8506872 |
| 1419606_a_at | *Tnnt1* | 2.2854776 | 0.705068 |
| 1424967_x_at | *Tnnt2* | 2.127564 | 2.1421514 |
| 1450798_at | *Tnxb* | 2.2118974 | 0.7978067 |
| 1421998_at | *Tor3a* | 2.3405256 | 4.523226 |
| 1418412_at | *Tpd52l1* | 2.8289788 | 0.31269965 |
| 1449577_x_at | *Tpm2* | 13.428707 | 0.0 |
| 1419739_at | *Tpm2* | 5.6447763 | 0.0 |
| 1419738_a_at | *Tpm2* | 13.139089 | 0.0 |
| 1425028_a_at | *Tpm2* | 15.823369 | 0.0 |
| 1418097_a_at | *Tpte2* | 2.118817 | 1.2921674 |
| 1445452_at | *Traf1* | 3.24416 | 0.88485754 |
| 1423602_at | *Traf1* | 5.3897495 | 0.0 |
| 1460226_at | *Trap1a* | 16.475235 | 0.0 |
| 1450672_a_at | *Trex1* | 2.9316645 | 0.5233838 |
| 1459994_x_at | *Trfr2* | 2.2885602 | 0.81543773 |
| 1437432_a_at | *Trim12* | 2.9395201 | 1.1254462 |
| 1451860_a_at | *Trim30* | 3.0059514 | 3.402282 |
| 1436919_at | *Trp53i11* | 2.0511684 | 2.3092976 |
| 1416935_at | *Trpv2* | 2.1902473 | 4.3510437 |
| 1417545_at | *Trpv4* | 2.079209 | 1.3178056 |
| 1418643_at | *Tspan13* | 3.0437498 | 4.3510437 |
| 1425157_x_at | *Tspan33* | 2.1599367 | 0.83622795 |
| 1455618_x_at | *Tspan33* | 2.6909282 | 0.83622795 |
| 1451609_at | *Tspan33* | 2.420747 | 0.8631242 |
| 1451608_a_at | *Tspan33* | 2.4679291 | 0.8631242 |
| 1448296_x_at | *Tuba3a* | 5.4968247 | 0.0 |
| 1417373_a_at | *Tuba4a* | 2.4789982 | 4.0998397 |
| 1417374_at | *Tuba4a* | 2.626764 | 3.6847382 |
| 1452679_at | *Tubb2b* | 7.0457406 | 1.8565313 |
| 1428689_at | *Tysnd1* | 2.058101 | 1.2484474 |
| 1448260_at | *Uchl1* | 15.544653 | 0.0 |
| 1448188_at | *Ucp2* | 2.511649 | 1.1133186 |
| 1459740_s_at | *Ucp2* | 3.2583585 | 0.0 |
| 1448562_at | *Upp1* | 2.911986 | 0.66684145 |
| 1448162_at | *Vcam1* | 3.7459292 | 1.2626883 |
| 1421694_a_at | *Vcan* | 3.2144804 | 4.93483 |
| 1425377_at | *Wnt1* | 2.1818748 | 1.4637445 |
| 1460657_at | *Wnt10a* | 3.7506785 | 1.2626883 |
| 1426091_a_at | *Wnt10b* | 2.045403 | 3.2433412 |
| 1439373_x_at | *Wnt5b* | 2.5890262 | 0.3153723 |
| 1420892_at | *Wnt7b* | 3.4265046 | 1.2097888 |
| 1420891_at | *Wnt7b* | 3.8636358 | 1.0086167 |
| 1436936_s_at | *Xist* | 3.8400028 | 1.3154566 |
| 1427262_at | *Xist* | 10.347891 | 0.0 |
| 1419604_at | *Zbp1* | 6.4220324 | 0.0 |
| 1429947_a_at | *Zbp1* | 7.265048 | 0.0 |
| 1427348_at | *Zc3h12a* | 4.0520186 | 0.0 |
| 1425305_at | *Zfp295* | 2.231108 | 3.0157475 |
| 1449354_at | *Zrsr1* | 2.1534767 | 1.3154566 |
| 1435705_at | *Zscan18* | 2.1928527 | 1.6799344 |

Table S3 includes 688 probe sets representing 569 genes that were selected as statistically significant by the pairwise two-class SAM analysis (FDR and *Q*-values <0.05 and up-regulation after 5AzaCdR treatment more than 2-fold times). Fold-enrichments were calculated by comparing the expression values of treated cells to those of untreated cells.
