# Supplementary material for: Mining Gene Expression Signature for the Detection of Pre-Malignant Melanocytes and Early Melanomas with Risk for Metastasis
Source: PLoS One. 2012 Sep 11;7(9):e44800. doi: 10.1371/journal.pone.0044800 (PMC3439384; doi:10.1371/journal.pone.0044800)
Supplement: Table S4 — Transcripts Up-Regulated in 4C11+ Metastatic Melanoma Cells Following 5-aza-2′-deoxycytidine Treatment Identified by Genome-Wide Screening. (DOC) [file pone.0044800.s007.doc]

**Table S4. Transcripts Up-Regulated in 4C11+ Metastatic Melanoma Cells Following 5-aza-2'-deoxycytidine Treatment Identified by Genome-Wide Screening.**

| **Probe Set_ID** | **Gene** | **Fold-Enrichment** | ***Q*-Value (%)** |
| --- | --- | --- | --- |
| 1421840_at | *Abca1* | 2.047802 | 1.3825744 |
| 1424451_at | *Acaa1b* | 5.7999873 | 1.6755732 |
| 1422428_at | *Acsbg1* | 2.0967462 | 1.2945591 |
| 1451257_at | *Acsl6* | 7.07758 | 2.9829028 |
| 1418677_at | *Actn3* | 2.5640824 | 0.72626305 |
| 1417976_at | *Ada* | 2.3490608 | 1.1675808 |
| 1447839_x_at | *Adm* | 4.120579 | 4.429064 |
| 1416077_at | *Adm* | 7.1085443 | 4.3779044 |
| 1416646_at | *Afp* | 2.1678421 | 1.5692887 |
| 1436879_x_at | *Afp* | 4.4352856 | 0.0 |
| 1416645_a_at | *Afp* | 8.52528 | 0.0 |
| 1437813_at | *Aim1l* | 2.0739586 | 4.429064 |
| 1419241_a_at | *Aire* | 2.405974 | 1.4064807 |
| 1422184_a_at | *Ak1* | 2.3984985 | 1.3670834 |
| 1419706_a_at | *Akap12* | 3.665098 | 0.0 |
| 1443086_at | *Alcam* | 2.1276138 | 1.6755732 |
| 1457021_x_at | *Amhr2* | 2.009938 | 1.4909841 |
| 1438937_x_at | *Ang* | 3.4178185 | 4.003383 |
| 1438936_s_at | *Ang* | 3.90156 | 0.0 |
| 1456629_at | *Ankrd47* | 3.2507586 | 4.4745793 |
| 1448839_at | *Ankrd47* | 6.2867165 | 1.2569321 |
| 1417889_at | *Apobec2* | 6.188316 | 0.47018188 |
| 1416371_at | *Apod* | 2.5280528 | 2.316256 |
| 1418847_at | *Arg2* | 3.9280932 | 1.2271341 |
| 1438841_s_at | *Arg2* | 4.208193 | 1.3407934 |
| 1424250_a_at | *Arhgef3* | 2.459092 | 1.4064807 |
| 1458238_at | *Arid5b* | 2.0050395 | 1.5219816 |
| 1439036_a_at | *Atp1b1* | 10.17358 | 1.1675808 |
| 1434350_at | *Axud1* | 2.0279944 | 1.3670834 |
| 1428792_at | *Bcas1* | 3.7020268 | 0.0 |
| 1435448_at | *Bcl2l11* | 2.0638072 | 1.2514071 |
| 1456005_a_at | *Bcl2l11* | 3.411174 | 0.0 |
| 1449880_s_at | *Bglap-rs1 | Bglap1 | Bglap2* | 4.17424 | 1.2578577 |
| 1442749_at | *Braf* | 2.0114284 | 4.825341 |
| 1424921_at | *Brd4 | Bst2* | 2.076948 | 3.7648911 |
| 1434969_at | *Brunol5* | 5.6035357 | 1.2378049 |
| 1420632_a_at | *Bscl2* | 2.531776 | 2.7113822 |
| 1437345_a_at | *Bscl2* | 3.3653178 | 1.5692887 |
| 1416250_at | *Btg2* | 2.9986238 | 3.338227 |
| 1448272_at | *Btg2* | 3.0898814 | 1.6829268 |
| 1448656_at | *Cacnb3* | 2.3607867 | 0.41081548 |
| 1459457_at | *Camk2d* | 2.1487591 | 1.4909841 |
| 1450355_a_at | *Capg* | 2.311848 | 4.4085174 |
| 1449145_a_at | *Cav1* | 4.3533626 | 1.4064807 |
| 1418509_at | *Cbr2* | 2.7735722 | 2.4041812 |
| 1423844_s_at | *Cbs* | 2.5077085 | 0.51158154 |
| 1418778_at | *Ccdc109b* | 2.911877 | 0.0 |
| 1441965_at | *Ccdc38* | 2.2254395 | 1.2514071 |
| 1424186_at | *Ccdc80* | 4.853045 | 0.0 |
| 1420127_s_at | *Ccpg1* | 2.027918 | 4.0982556 |
| 1449131_s_at | *Cd1d1* | 2.6730633 | 0.0 |
| 1448182_a_at | *Cd24a* | 2.2747922 | 0.9760976 |
| 1425519_a_at | *Cd74* | 3.008483 | 3.0716357 |
| 1427357_at | *Cda* | 2.1848073 | 1.2578577 |
| 1417649_at | *Cdkn1c* | 3.9372802 | 0.4900088 |
| 1423233_at | *Cebpd* | 2.8089588 | 0.0 |
| 1435345_at | *Ceecam1* | 2.6974134 | 4.0342364 |
| 1418925_at | *Celsr1* | 2.0137496 | 3.3806906 |
| 1427137_at | *Ces5* | 2.0569172 | 2.259485 |
| 1424529_s_at | *Cgref1* | 2.5048811 | 2.1443267 |
| 1424528_at | *Cgref1* | 3.0049915 | 1.3670834 |
| 1420682_at | *Chrnb1* | 3.8695147 | 3.5600376 |
| 1428902_at | *Chst11* | 3.356041 | 1.3556911 |
| 1422852_at | *Cib2* | 4.0677786 | 0.0 |
| 1440802_at | *Clasp2* | 3.144226 | 3.7648911 |
| 1439427_at | *Cldn9* | 5.4236984 | 0.0 |
| 1427964_at | *Cmtm8* | 2.1871028 | 2.259485 |
| 1455399_at | *Cnksr1* | 3.1372807 | 2.259485 |
| 1418980_a_at | *Cnp* | 2.1082633 | 1.800918 |
| 1423669_at | *Col1a1* | 2.8261478 | 2.316256 |
| 1460734_at | *Col9a3* | 3.4716964 | 3.891936 |
| 1416246_a_at | *Coro1a* | 3.6599677 | 1.800918 |
| 1455269_a_at | *Coro1a* | 3.936563 | 0.0 |
| 1435275_at | *Cox6b2* | 6.698814 | 2.1443267 |
| 1418709_at | *Cox7a1* | 3.5911937 | 4.7200074 |
| 1455393_at | *Cp* | 2.007721 | 3.5088475 |
| 1417494_a_at | *Cp* | 2.0215096 | 3.5088475 |
| 1441326_at | *Cp* | 2.3544648 | 2.4492676 |
| 1435281_at | *Cpt1c* | 2.6008604 | 2.900908 |
| 1426251_at | *Cpz* | 4.676181 | 1.6214246 |
| 1451191_at | *Crabp2* | 3.875428 | 1.2514071 |
| 1416326_at | *Crip1* | 2.9085627 | 1.1168164 |
| 1418476_at | *Crlf1* | 2.1942596 | 0.0 |
| 1451019_at | *Ctsf* | 3.3021479 | 0.0 |
| 1443814_x_at | *Ctsh* | 3.011902 | 0.0 |
| 1418365_at | *Ctsh* | 3.1452007 | 0.0 |
| 1435435_at | *Cttnbp2* | 2.186289 | 3.3806906 |
| 1457644_s_at | *Cxcl1* | 14.01383 | 0.0 |
| 1419209_at | *Cxcl1* | 16.327282 | 0.0 |
| 1449984_at | *Cxcl2* | 50.037624 | 0.0 |
| 1417507_at | *Cyb561* | 5.2395134 | 0.0 |
| 1450752_at | *Cyct* | 4.223101 | 1.6946138 |
| 1417071_s_at | *Cyp4v3* | 2.2438827 | 2.316256 |
| 1442340_x_at | *Cyr61* | 2.0307856 | 1.4064807 |
| 1457823_at | *Cyr61* | 2.4400287 | 0.0 |
| 1426215_at | *Ddc* | 8.202206 | 0.0 |
| 1456890_at | *Ddx58* | 2.1590536 | 1.1245364 |
| 1436562_at | *Ddx58* | 2.6694727 | 0.45189703 |
| 1426731_at | *Des* | 3.1129684 | 2.4492676 |
| 1418578_at | *Dgka* | 2.9833786 | 0.43266734 |
| 1451426_at | *Dhx58* | 4.0458584 | 0.0 |
| 1457275_at | *Dmn* | 6.334165 | 0.75316167 |
| 1434944_at | *Dmpk* | 2.0608249 | 1.6714 |
| 1459791_at | *Dnajc1* | 2.3903954 | 4.4085174 |
| 1428330_at | *Dopey2* | 2.4009488 | 3.3386421 |
| 1453223_s_at | *Dppa2* | 3.5544424 | 0.0 |
| 1429597_at | *Dppa4* | 2.2755508 | 1.3825744 |
| 1439825_at | *Dtx3l* | 3.376836 | 0.73612183 |
| 1454737_at | *Dusp9* | 2.1048217 | 0.6952262 |
| 1444606_at | *Efna2* | 2.0127254 | 0.7042551 |
| 1450744_at | *Ell2* | 2.1498027 | 0.8093678 |
| 1418829_a_at | *Eno2* | 2.4927914 | 3.9611359 |
| 1423005_a_at | *Espn* | 2.719875 | 4.0342364 |
| 1428867_at | *Exoc3l2* | 2.2532864 | 1.2945591 |
| 1424595_at | *F11r* | 2.0022678 | 1.2378049 |
| 1416023_at | *Fabp3* | 11.846227 | 1.552318 |
| 1443904_at | *Fads6* | 2.054038 | 2.4492676 |
| 1460251_at | *Fas* | 2.209384 | 2.4492676 |
| 1418569_at | *Fblim1* | 4.74632 | 1.4064807 |
| 1449141_at | *Fblim1* | 3.6265597 | 0.0 |
| 1427004_at | *Fbxo2* | 3.193308 | 0.0 |
| 1448747_at | *Fbxo32* | 2.663552 | 0.0 |
| 1443698_at | *Fbxo39* | 4.3436995 | 0.0 |
| 1418340_at | *Fcer1g* | 2.713535 | 0.9760976 |
| 1425225_at | *Fcgr4* | 2.475228 | 0.0 |
| 1449555_a_at | *Fetub* | 2.0323749 | 3.9611359 |
| 1424050_s_at | *Fgfr1* | 2.1502914 | 1.6012099 |
| 1449429_at | *Fkbp1b* | 2.4690335 | 0.4900088 |
| 1456084_x_at | *Fmod* | 2.561882 | 0.71666485 |
| 1437218_at | *Fn1* | 2.579361 | 2.4492676 |
| 1423100_at | *Fos* | 2.7443464 | 4.3779044 |
| 1452123_s_at | *Frmd4b* | 2.6255455 | 0.0 |
| 1438169_a_at | *Frmd4b* | 6.431347 | 0.0 |
| 1416514_a_at | *Fscn1* | 8.8577 | 0.0 |
| 1417301_at | *Fzd6* | 2.2652936 | 1.5204012 |
| 1449519_at | *Gadd45a* | 2.5289962 | 0.7230352 |
| 1449773_s_at | *Gadd45b* | 2.026053 | 3.9327831 |
| 1418240_at | *Gbp2* | 4.5975494 | 2.900908 |
| 1418392_a_at | *Gbp3* | 4.075394 | 3.338227 |
| 1425156_at | *Gbp6* | 2.7010062 | 1.3407934 |
| 1424698_s_at | *Gca* | 3.440799 | 4.0982556 |
| 1435750_at | *Gchfr* | 3.193514 | 1.3670834 |
| 1418949_at | *Gdf15* | 4.370698 | 4.3945947 |
| 1424927_at | *Glipr1* | 6.878322 | 2.4041812 |
| 1419194_s_at | *Gmfg* | 3.492458 | 2.900908 |
| 1430238_at | *Got1l1* | 3.5074174 | 2.4041812 |
| 1422977_at | *Gp1bb* | 2.222533 | 4.0342364 |
| 1417673_at | *Grb14* | 2.2852337 | 0.80536103 |
| 1427046_at | *Grhl2* | 2.7090378 | 1.280968 |
| 1416368_at | *Gsta4* | 2.6796086 | 0.0 |
| 1418186_at | *Gstt1* | 2.5498407 | 1.4064807 |
| 1425545_x_at | *H2-D1* | 2.7716844 | 2.9312239 |
| 1451683_x_at | *H2-D1* | 3.0188138 | 2.3085222 |
| 1426324_at | *H2-D1* | 2.5513396 | 0.67784554 |
| 1425336_x_at | *H2-K1* | 3.954913 | 0.51158154 |
| 1451931_x_at | *H2-L* | 2.7794747 | 3.5088475 |
| 1430357_at | *H3f3b* | 3.6273246 | 1.4909841 |
| 1452757_s_at | *Hba-a1 | Hba-a2* | 2.9129093 | 0.47018188 |
| 1428361_x_at | *Hba-a1 | Hba-a2* | 3.1946242 | 0.0 |
| 1428405_at | *Hcfc1r1* | 2.002055 | 4.003383 |
| 1449271_a_at | *Hebp2* | 2.0149188 | 4.0019255 |
| 1416101_a_at | *Hist1h1c* | 2.0178862 | 1.2110392 |
| 1423858_a_at | *Hmgcs2* | 3.1898847 | 3.7375762 |
| 1420712_a_at | *Hpn* | 2.260944 | 0.7935752 |
| 1452318_a_at | *Hspa1b* | 3.9553747 | 3.0716357 |
| 1417101_at | *Hspa2* | 2.1061482 | 4.429064 |
| 1422943_a_at | *Hspb1* | 6.3450785 | 0.0 |
| 1425964_x_at | *Hspb1* | 6.960383 | 0.0 |
| 1424067_at | *Icam1* | 4.6533246 | 2.0444891 |
| 1452231_x_at | *Ifi203* | 5.7036886 | 1.1817162 |
| 1452349_x_at | *Ifi205 | Mnda* | 3.83657 | 4.3945947 |
| 1424617_at | *Ifi35* | 2.8761094 | 1.6001599 |
| 1445897_s_at | *Ifi35* | 3.7811513 | 0.0 |
| 1459151_x_at | *Ifi35* | 3.374223 | 0.0 |
| 1426276_at | *Ifih1* | 3.8011878 | 0.0 |
| 1450783_at | *Ifit1* | 8.04082 | 0.0 |
| 1449025_at | *Ifit3* | 3.9831953 | 0.0 |
| 1437103_at | *Igf2bp2* | 2.4566362 | 1.761909 |
| 1439764_s_at | *Igf2bp2* | 3.7111888 | 0.0 |
| 1451407_at | *Igsf5* | 2.652329 | 1.2418544 |
| 1417141_at | *Igtp* | 5.9364057 | 0.0 |
| 1417793_at | *Iigp2* | 4.8648176 | 0.0 |
| 1431693_a_at | *Il17b* | 4.4883714 | 0.0 |
| 1429893_at | *Il17rd* | 2.177274 | 0.48417538 |
| 1417932_at | *Il18* | 2.2110188 | 1.4064807 |
| 1416296_at | *Il2rg* | 2.1348405 | 2.900908 |
| 1416295_a_at | *Il2rg* | 5.23875 | 0.4192859 |
| 1418301_at | *Irf6* | 4.463116 | 0.70731705 |
| 1419569_a_at | *Isg20* | 4.30724 | 0.0 |
| 1421322_a_at | *Isgf3g* | 2.933903 | 1.9509945 |
| 1418450_at | *Islr* | 2.7532475 | 0.5052265 |
| 1447541_s_at | *Itgae* | 2.3100026 | 1.5204012 |
| 1450678_at | *Itgb2* | 3.1470788 | 0.47018188 |
| 1448694_at | *Jun* | 2.0795367 | 0.42587155 |
| 1415899_at | *Junb* | 2.1227372 | 1.2945591 |
| 1417680_at | *Kcna5* | 3.0561001 | 0.40875107 |
| 1435945_a_at | *Kcnn4* | 2.0482562 | 1.4064807 |
| 1418538_at | *Kdelr3* | 3.3189209 | 0.0 |
| 1447853_x_at | *Kif13a* | 2.1388788 | 1.2945591 |
| 1415855_at | *Kitl* | 2.5450063 | 1.7184817 |
| 1417394_at | *Klf4* | 2.6328676 | 0.0 |
| 1428221_at | *Klhdc8b* | 2.0222461 | 1.2759445 |
| 1424114_s_at | *Lamb1-1* | 2.2452304 | 1.3874876 |
| 1424113_at | *Lamb1-1* | 2.3517866 | 1.1245364 |
| 1436905_x_at | *Laptm5* | 3.8999197 | 0.43266734 |
| 1433783_at | *Ldb3* | 3.0035615 | 0.0 |
| 1426808_at | *Lgals3* | 116.82481 | 0.41081548 |
| 1421207_at | *Lif* | 2.6048338 | 0.4192859 |
| 1448409_at | *Lrmp* | 3.7329075 | 1.5493612 |
| 1439821_at | *Lrp2bp* | 2.2015545 | 1.2569321 |
| 1453214_at | *Lrrc15* | 2.6375675 | 0.0 |
| 1433858_at | *Lrrc28* | 2.0685663 | 0.41081548 |
| 1417777_at | *Ltb4dh* | 18.31892 | 0.0 |
| 1418188_a_at | *Malat1* | 2.3855295 | 0.0 |
| 1418189_s_at | *Malat1* | 2.524417 | 0.0 |
| 1451290_at | *Map1lc3a* | 2.5125558 | 1.4064807 |
| 1447883_x_at | *Map1lc3a* | 2.9255328 | 0.52141964 |
| 1437540_at | *Mcoln3* | 12.949467 | 3.5600376 |
| 1457929_at | *Mdm2* | 2.546263 | 4.6703234 |
| 1420911_a_at | *Mfge8* | 2.1141472 | 2.7113822 |
| 1452592_at | *Mgst2* | 2.6985521 | 1.7492788 |
| 1449432_a_at | *Mmel1* | 2.0775228 | 2.8457656 |
| 1437123_at | *Mmrn2* | 8.277017 | 0.0 |
| 1439518_at | *Mmrn2* | 11.180012 | 0.0 |
| 1456768_a_at | *Mmrn2* | 15.50366 | 0.0 |
| 1440091_at | *Mrg1* | 2.0740168 | 1.1675808 |
| 1434378_a_at | *Mxd4* | 3.098318 | 0.4192859 |
| 1434379_at | *Mxd4* | 2.3684008 | 0.5281913 |
| 1427115_at | *Myh3* | 3.6180289 | 0.0 |
| 1428786_at | *Nckap1l* | 2.1254342 | 4.7200074 |
| 1448154_at | *Ndrg2* | 2.0543432 | 1.6366491 |
| 1436188_a_at | *Ndrg4* | 2.0146887 | 1.584574 |
| 1426615_s_at | *Ndrg4* | 2.1548584 | 0.7783872 |
| 1456854_at | *Neurl* | 2.2091916 | 2.316256 |
| 1420088_at | *Nfkbia* | 2.0969076 | 1.2945591 |
| 1449731_s_at | *Nfkbia* | 2.4922462 | 0.4621674 |
| 1438157_s_at | *Nfkbia* | 2.8569121 | 0.0 |
| 1448306_at | *Nfkbia* | 3.158107 | 0.0 |
| 1417483_at | *Nfkbiz* | 2.3847558 | 0.0 |
| 1430233_a_at | *Nhedc1* | 5.1638756 | 2.7113822 |
| 1416473_a_at | *Nope* | 4.7062473 | 1.4064807 |
| 1416474_at | *Nope* | 5.060869 | 1.5204012 |
| 1418633_at | *Notch1* | 2.0741718 | 1.3825744 |
| 1418634_at | *Notch1* | 2.1563878 | 1.3825744 |
| 1421965_s_at | *Notch3* | 3.663146 | 0.0 |
| 1426852_x_at | *Nov* | 2.5085616 | 2.900908 |
| 1426851_a_at | *Nov* | 2.8060079 | 1.2676592 |
| 1425151_a_at | *Noxo1* | 3.0518467 | 1.2110392 |
| 1423627_at | *Nqo1* | 2.079728 | 1.5219816 |
| 1440972_at | *Nsd1* | 2.0949852 | 1.7682927 |
| 1440353_at | *Ntf5* | 2.0594926 | 1.8210776 |
| 1424775_at | *Oas1a* | 2.940188 | 0.0 |
| 1418686_at | *Oas1c* | 2.1380086 | 0.52141964 |
| 1453196_a_at | *Oasl2* | 2.8597684 | 0.0 |
| 1455679_at | *Obfc2a* | 2.9959888 | 0.0 |
| 1443632_at | *Obscn* | 3.9277453 | 0.73612183 |
| 1455796_x_at | *Olfm1* | 2.0523973 | 0.43266734 |
| 1425784_a_at | *Olfm1* | 2.2645285 | 0.44448888 |
| 1435790_at | *Olfm2* | 2.1220365 | 1.2271341 |
| 1444980_at | *Onecut2* | 2.0412087 | 1.3556911 |
| 1425214_at | *P2ry6* | 2.7105527 | 0.0 |
| 1419767_at | *Padi3* | 8.878082 | 0.0 |
| 1427228_at | *Palld* | 2.2998176 | 2.549889 |
| 1426774_at | *Parp12* | 2.407428 | 1.5156795 |
| 1451564_at | *Parp14* | 4.143378 | 0.0 |
| 1416897_at | *Parp9* | 2.4703407 | 1.2514071 |
| 1437928_at | *Pcdh12* | 2.4300544 | 1.5204012 |
| 1451527_at | *Pcolce2* | 2.4426868 | 0.5281913 |
| 1449420_at | *Pde1b* | 2.0797625 | 1.1817162 |
| 1418711_at | *Pdgfa* | 2.4331832 | 0.5052265 |
| 1421917_at | *Pdgfra* | 2.484464 | 1.3556911 |
| 1417689_a_at | *Pdzk1ip1* | 2.2111692 | 2.896432 |
| 1421566_at | *Pet2* | 2.4710202 | 1.552318 |
| 1418373_at | *Pgam2* | 4.1606307 | 3.6176124 |
| 1418471_at | *Pgf* | 2.597928 | 1.3786689 |
| 1447564_x_at | *Piwil4* | 2.1883936 | 1.2945591 |
| 1429183_at | *Pkp2* | 2.4709098 | 1.6755732 |
| 1460511_at | *Pkp2* | 2.4197628 | 0.7935752 |
| 1449799_s_at | *Pkp2* | 3.0095768 | 0.0 |
| 1438588_at | *Plagl1* | 2.0155888 | 2.900908 |
| 1436335_at | *Plch2* | 2.085552 | 4.4745793 |
| 1457252_x_at | *Pld2* | 2.1855614 | 1.2578577 |
| 1449424_at | *Plek2* | 4.9778256 | 2.9312239 |
| 1416178_a_at | *Plekhb1* | 2.1421933 | 1.5125479 |
| 1444817_at | *Plekhh2* | 2.17784 | 1.4064807 |
| 1427005_at | *Plk2* | 3.3713088 | 0.45189703 |
| 1448961_at | *Plscr2* | 3.0424585 | 3.7375762 |
| 1456424_s_at | *Pltp* | 2.7912529 | 0.75316167 |
| 1417963_at | *Pltp* | 2.8301868 | 0.0 |
| 1444288_at | *Pnpt1* | 2.0122252 | 2.549889 |
| 1434990_at | *Ppm1e* | 2.1240013 | 0.8093678 |
| 1418086_at | *Ppp1r14a* | 5.868362 | 0.0 |
| 1434325_x_at | *Prkar1b* | 2.4999273 | 1.2514071 |
| 1435162_at | *Prkg2* | 3.717996 | 1.6366491 |
| 1426246_at | *Pros1* | 2.0181031 | 2.7113822 |
| 1422962_a_at | *Psmb8* | 3.2146351 | 2.7113822 |
| 1450696_at | *Psmb9* | 2.401571 | 1.4064807 |
| 1418181_at | *Ptp4a3* | 2.1416805 | 1.6322702 |
| 1437613_s_at | *Ptpdc1* | 2.2002864 | 0.73612183 |
| 1420843_at | *Ptprf* | 2.2040608 | 0.0 |
| 1417741_at | *Pygl* | 2.7104504 | 0.7783872 |
| 1427405_s_at | *Rab11fip5* | 4.576518 | 2.549889 |
| 1434314_s_at | *Rab11fip5* | 4.422624 | 1.1817162 |
| 1433947_at | *Rab37* | 3.1768744 | 0.0 |
| 1436566_at | *Rab40b* | 3.0654376 | 4.3945947 |
| 1444671_at | *Rasal2* | 2.001867 | 4.3945947 |
| 1422638_s_at | *Rassf5* | 2.0874755 | 2.1443267 |
| 1424008_a_at | *Rbpms2* | 2.6811936 | 4.0342364 |
| 1442819_at | *Rhbdl2* | 2.4675882 | 1.4909841 |
| 1445233_at | *Rhbdl2* | 2.1092234 | 1.6946138 |
| 1424976_at | *Rhov* | 2.6708827 | 1.2759445 |
| 1423429_at | *Rhox5* | 5.4828963 | 1.7682927 |
| 1431805_a_at | *Rhpn2* | 2.5019023 | 1.2110392 |
| 1434628_a_at | *Rhpn2* | 2.7601707 | 1.2676592 |
| 1418310_a_at | *Rlbp1* | 3.4080038 | 2.1443267 |
| 1422603_at | *Rnase4* | 4.1181564 | 1.4064807 |
| 1429399_at | *Rnf125* | 2.413657 | 2.900908 |
| 1423327_at | *Rpl39l* | 7.043539 | 1.1592607 |
| 1452730_at | *Rps4y2* | 9.119623 | 3.9611359 |
| 1421008_at | *Rsad2* | 2.5177474 | 1.2945591 |
| 1436058_at | *Rsad2* | 5.154115 | 0.47018188 |
| 1421009_at | *Rsad2* | 5.4744935 | 0.0 |
| 1449319_at | *Rspo1* | 2.9502003 | 0.0 |
| 1418580_at | *Rtp4* | 7.6147237 | 0.0 |
| 1434743_x_at | *Rusc1* | 2.442337 | 0.0 |
| 1427306_at | *Ryr1* | 2.2784424 | 0.5281913 |
| 1418704_at | *S100a13* | 2.0416243 | 1.6054236 |
| 1424542_at | *S100a4* | 2.1875582 | 1.3934298 |
| 1421375_a_at | *S100a6* | 3.602805 | 1.4064807 |
| 1460603_at | *Samd9l* | 2.149848 | 0.45189703 |
| 1459897_a_at | *Sbsn* | 2.1570258 | 1.6012099 |
| 1451204_at | *Scara5* | 2.3670614 | 0.67784554 |
| 1450734_at | *Sec16b* | 2.2000012 | 4.3945947 |
| 1448729_a_at | *Sept4* | 2.4109948 | 0.44940034 |
| 1455422_x_at | *Sept4* | 2.627021 | 0.0 |
| 1434548_at | *Serinc3* | 2.0263896 | 1.1245364 |
| 1416318_at | *Serpinb1a* | 5.1635475 | 1.8210776 |
| 1422804_at | *Serpinb6b* | 2.0498128 | 2.8457656 |
| 1422601_at | *Serpinb9* | 2.065703 | 1.2418544 |
| 1419149_at | *Serpine1* | 2.9740894 | 0.43266734 |
| 1416666_at | *Serpine2* | 18.940506 | 0.0 |
| 1454877_at | *Sertad4* | 3.480708 | 3.891936 |
| 1416041_at | *Sgk* | 2.2312353 | 0.0 |
| 1417809_at | *Slc22a18* | 2.042708 | 3.3806906 |
| 1451139_at | *Slc39a4* | 4.020742 | 0.0 |
| 1452445_at | *Slc41a2* | 2.3201432 | 1.2110392 |
| 1418395_at | *Slc47a1* | 5.011918 | 1.7184817 |
| 1417788_at | *Sncg* | 4.2643166 | 0.0 |
| 1449109_at | *Socs2* | 2.300097 | 1.5464157 |
| 1438968_x_at | *Spint2* | 8.545644 | 4.825341 |
| 1449254_at | *Spp1* | 4.7469745 | 0.0 |
| 1443554_at | *Ssbp3* | 2.1418777 | 1.6755732 |
| 1448956_at | *Stard10* | 2.606143 | 0.67784554 |
| 1440481_at | *Stat1* | 3.9421058 | 4.6703234 |
| 1420915_at | *Stat1* | 2.305843 | 3.0716357 |
| 1450034_at | *Stat1* | 2.4493902 | 1.3407934 |
| 1450033_a_at | *Stat1* | 2.2571611 | 0.0 |
| 1434442_at | *Stbd1* | 2.1829784 | 1.6714 |
| 1425536_at | *Stx3* | 2.229724 | 1.1675808 |
| 1439759_x_at | *Sult6b1* | 2.15582 | 2.316256 |
| 1437387_at | *Susd5* | 3.753982 | 2.0444891 |
| 1449534_at | *Sycp3* | 15.512743 | 1.4064807 |
| 1435511_at | *Syn2* | 2.0474498 | 0.42587155 |
| 1434089_at | *Synpo* | 2.6362123 | 1.2945591 |
| 1452879_at | *Synpo2* | 2.1319582 | 0.67784554 |
| 1460081_at | *Syt7* | 2.251958 | 0.45189703 |
| 1416016_at | *Tap1* | 3.6017354 | 0.5281913 |
| 1453913_a_at | *Tap2* | 3.1686027 | 0.0 |
| 1421812_at | *Tapbp* | 2.498818 | 1.3519356 |
| 1433471_at | *Tcf7* | 2.0604115 | 1.8210776 |
| 1460204_at | *Tec* | 2.1430614 | 1.6432619 |
| 1432159_a_at | *Tex13* | 3.32389 | 3.3386421 |
| 1450555_at | *Tex13* | 19.157654 | 1.2759445 |
| 1417482_at | *Tex19* | 33.65949 | 0.0 |
| 1455900_x_at | *Tgm2* | 3.9325497 | 2.7113822 |
| 1437277_x_at | *Tgm2* | 3.966709 | 2.7113822 |
| 1433428_x_at | *Tgm2* | 3.7978659 | 2.3846033 |
| 1417500_a_at | *Tgm2* | 3.0949848 | 2.0444891 |
| 1424737_at | *Thrsp* | 3.2187247 | 2.3597353 |
| 1417896_at | *Tjp3* | 2.1181881 | 2.900908 |
| 1449049_at | *Tlr1* | 2.3496163 | 0.7746806 |
| 1441370_at | *Tmcc1* | 2.0238016 | 0.75316167 |
| 1434252_at | *Tmcc3* | 2.2418554 | 1.5204012 |
| 1424354_at | *Tmem140* | 5.786706 | 0.0 |
| 1424177_at | *Tmem38a* | 2.2506607 | 1.6366491 |
| 1451546_s_at | *Tmem40* | 2.373226 | 0.49902737 |
| 1424966_at | *Tmem40* | 3.0862904 | 0.0 |
| 1441917_s_at | *Tmem40* | 3.4137743 | 0.0 |
| 1453285_at | *Tmem88* | 2.2400503 | 1.2378049 |
| 1433699_at | *Tnfaip3* | 2.2395537 | 4.429064 |
| 1422303_a_at | *Tnfrsf18* | 2.7651021 | 2.9829028 |
| 1428034_a_at | *Tnfrsf9* | 2.2298353 | 2.1443267 |
| 1419606_a_at | *Tnnt1* | 3.4396088 | 0.0 |
| 1424967_x_at | *Tnnt2* | 2.9610035 | 3.4400892 |
| 1418726_a_at | *Tnnt2* | 3.1178489 | 2.1443267 |
| 1428346_at | *Trafd1* | 2.0322921 | 0.43266734 |
| 1437432_a_at | *Trim12* | 3.3558452 | 1.2271341 |
| 1425974_a_at | *Trim25* | 2.8769002 | 0.0 |
| 1419879_s_at | *Trim25* | 3.1122384 | 0.0 |
| 1451860_a_at | *Trim30* | 4.220062 | 0.0 |
| 1417961_a_at | *Trim30* | 3.257026 | 0.0 |
| 1455618_x_at | *Tspan33* | 2.9947953 | 0.0 |
| 1454608_x_at | *Ttr* | 5.398372 | 1.8210776 |
| 1455913_x_at | *Ttr* | 4.6179705 | 0.6722435 |
| 1459737_s_at | *Ttr* | 3.6815598 | 0.0 |
| 1417374_at | *Tuba4a* | 3.7537587 | 3.338227 |
| 1417373_a_at | *Tuba4a* | 5.260862 | 0.0 |
| 1427347_s_at | *Tubb2a* | 2.5672152 | 0.70731705 |
| 1452679_at | *Tubb2b* | 6.562374 | 1.4064807 |
| 1415996_at | *Txnip* | 4.321922 | 0.0 |
| 1450484_a_at | *Tyki* | 2.5149117 | 0.67784554 |
| 1448260_at | *Uchl1* | 2.2557044 | 0.5281913 |
| 1459740_s_at | *Ucp2* | 3.248009 | 4.0982556 |
| 1450303_at | *Vax2* | 2.1962106 | 1.2569321 |
| 1418175_at | *Vdr* | 2.2768493 | 1.4064807 |
| 1460012_at | *Wfdc3* | 2.814403 | 2.0444891 |
| 1427196_at | *Wnk4* | 2.0651846 | 1.6012099 |
| 1436978_at | *Wnt9a* | 3.379005 | 0.51158154 |
| 1427263_at | *Xist* | 6.0300527 | 1.9509945 |
| 1436936_s_at | *Xist* | 19.098831 | 0.0 |
| 1427262_at | *Xist* | 100.23933 | 0.0 |
| 1434277_a_at | *Ypel2* | 2.2381015 | 1.2569321 |
| 1454901_at | *Ypel2* | 2.3678632 | 1.1403009 |
| 1418865_at | *Zfp385* | 2.7626264 | 0.0 |
| 1419564_at | *Zfp467* | 2.5046449 | 1.1245364 |
| 1426472_at | *Zfp52* | 2.0248532 | 1.1817162 |
| 1419007_at | *Zp3* | 2.2192729 | 2.9312239 |

Table S4 includes 427 probe sets representing 359 genes that were selected as statistically significant by the pairwise two-class SAM analysis (FDR and *Q*-values <0.05 and up-regulation after 5AzaCdR treatment more than 2-fold times). Fold-enrichments were calculated by comparing the expression values of treated cells to those of untreated cells.
