# Supplementary material for: Mining Gene Expression Signature for the Detection of Pre-Malignant Melanocytes and Early Melanomas with Risk for Metastasis
Source: PLoS One. 2012 Sep 11;7(9):e44800. doi: 10.1371/journal.pone.0044800 (PMC3439384; doi:10.1371/journal.pone.0044800)
Supplement: Table S5 — Primers Used for Amplification by RT-qPCR. (DOC) [file pone.0044800.s008.doc]

**Table S5.** Primers Used for Amplification by RT-qPCR.

|  | **Gene** | **Forward (5'-3')** | **Reverse (5'-3')** | **Product Size (bp)** |
| --- | --- | --- | --- | --- |
| **Mouse** | *Actb* | ACCGTGAAAAGATGACCCAG | GTACGACCAGAGGCATACAG | 100 |
|  | *Fblim1* | ACACACACCAAAGAGGCTAGGGAA | TGTGGCTTCACAGAGACAGTGGTT | 121 |
|  | *Hspb1* | AGCTCACAGTGAAGACCAAGGAAG | AGCACCGAGAGATGTAGCCATGTT | 90 |
|  | *Serpine1* | TCAGTGGCCAATGGAAGACTCCTT | AGGGCAGTTCCACAACGTCATACT | 171 |
|  | *Xist* | TCATAGCCCCTTCCCAATAG | GTTCACTTCAGAGCCACTTG | 132 |
|  |  |  |  |  |
| **Human** | *ACTB* | GTCTTCCCCTCCATCGTG | GTACTTCAGGGTGAGGATGC | 120 |
|  | *CTCF* | ATATGAAGCCTCCAAAGCCAA | CACTTGTGTGGTCTCTCATCA | 145 |
|  | *FBLIM1* | GGATGGGAAAGATGCCTTCA | TGCAGAAGAGATGGTTGTTCA | 143 |
|  | *HSPB1* | GACGAGCATGGCTACATCT | GACAGGGAGGAGGAAACTTG | 89 |
|  | *NDRG2* | ACTGTTGAAGGTCTTGTCCTC | TCTTCCTGGCTGAAAAGATGT | 134 |
|  | *NSD1* | AAAAGAAGGTACAGGAGCAGG | TTCAAGAACTGGAGGCTCTTC | 146 |
|  | *SERPINE1* | TTCAAGCAGCTATGGGATTCA | TGCTGATCTCATCCTTGTTCC | 105 |
|  | *SRC* | GAAGAAGCTGAGGCATGAGAA | CCTTGAGAAAGTCCAGCAAAC | 113 |
|  | *VDR* | CATCATTGCCATACTGCTGGA | CACCATCATTCACACGAACTG | 98 |
|  | *XIST* | AATCTACTTGGATGGGTTGCC | GGATCGTCAAAGGGAATGGAT | 103 |
